# Exploration of the Transcriptional Landscape of ALPPS Reveals the Pathways of Accelerated Liver Regeneration

**DOI:** 10.3389/fonc.2019.01206

**Published:** 2019-11-19

**Authors:** Pieter Borger, Marcel Schneider, Lukas Frick, Magda Langiewicz, Maksim Sorokin, Anton Buzdin, Ekaterina Kachaylo, Rolf Graf, Bostjan Humar, Pierre-Alain Clavien

**Affiliations:** ^1^Laboratory of the Swiss Hepato-Pancreato-Biliary (HPB) and Transplantation Center, Department of Surgery, University Hospital Zürich, Zurich, Switzerland; ^2^OmicsWay Corp., Walnut, CA, United States; ^3^I.M. Sechenov First Moscow State Medical University, Moscow, Russia; ^4^Shemyakin-Ovchinnikov Institute of Bioorganic Chemistry, Moscow, Russia; ^5^Oncobox Ltd., Moscow, Russia

**Keywords:** two-staged hepatectomy, ALPPS, transcriptome profiling, signaling pathways, oncofinder

## Abstract

**Background and Aims:** ALPPS (associating liver partition and portal vein ligation for staged hepatectomy), a novel 2-staged hepatectomy, dramatically accelerates liver regeneration and thus enables extensive liver tumor resection. The signaling networks underlying the ALPPS-induced accelerated regeneration process are largely unknown.

**Methods:** We performed transcriptome profiling (TP) of liver tissue obtained from a mouse model of ALPPS, standard hepatectomy (68% model), and additional control surgeries (sham, PVL and Tx). We also performed TP using human liver biopsies (*n* = 5) taken from the occluded lobe and the future liver remnant (FLR) during the first step of ALPPS surgery (4–5 h apart). We used Oncofinder computational tools, which covers 378 ISPs, for unsupervised, unbiased quantification of ISP activity.

**Results:** Gene expression cluster analysis revealed an ALPPS specific signature: the IGF1R Signaling Pathway (Cell survival), the ILK Pathway (Induced cell proliferation), and the IL-10 Pathway (Stability determination) were significantly enriched, whereas the activity of the Interferon Pathway (Transcription) was reduced (*p* < 0.05). Further, the PAK- and ILK-associated ISPs were activated at an earlier time point, reflecting significant acceleration of liver regeneration (*p* < 0.001). These pathways, which were also recovered in human liver biopsies, control cell growth and proliferation, inflammatory response, and hypoxia-related processes.

**Conclusions:** ALPPS is not a straightforward addition of portal vein ligation (PVL) plus transection—it is more. The early stages of normal and accelerated liver regeneration are clearly discernible by a significantly increased and earlier activation of a small number of signaling pathways. Compounds mimicking these responses may help to improve the ALPPS method and further reduce the hospitalization time of the patient.

## Introduction

Liver regeneration is controlled by a cooperating, redundant system of biological networks performing an assortment of tasks, which together result in a coordinated response to replenish lost liver tissue. The regenerating capacity of the liver inspired liver surgeons to develop 2-staged strategies to perform extended liver resections to clean the organ from multiple tumors. In the conventional 2-staged strategies, the complete restoration of a functional liver may take up to 8–10 weeks (1, 2). Recently, a novel two-staged strategy has been introduced: ALPPS (for: *A*ssociating *L*iver *P*artition and *P*ortal vein ligation for *S*taged hepatectomy) (3). In the first stage of the ALPPS procedure, the portal vein is ligated, followed by removal of any tumor in the future liver and an *in situ* split of the parenchyma between the healthy and diseased liver (partition or transection) is performed. In the second step, the isolated, deportalized liver is removed, leaving behind a tumor-free, hypertrophic liver remnant (4–6). The main advantage of the ALPPS strategy is that the accelerated regeneration of the liver remnant, which reaches a body-sustaining size within 7–10 days, enables the prompt elimination of the major tumor load (4, 7).

The biological processes underlying liver regeneration processes are complex. They involve (thyroid) hormones (8), cytokines [IL-6 (9) and TNF (10)], growth factor responses [HGF, TGF-β, epidermal growth factor (EGF)] (11, 12), glucose- and bile acid metabolism (13, 14), and platelet-derived factors, such as serotonin (15). Further studies in knockout mouse models revealed several key switches in signal-transduction systems, which either delay or accelerate the regenerating process. NF-κB (16), nuclear receptors [FXR (14) and CAR (17)] have been described as accelerators, whereas p21 (18), Socs3 (19), and Tob1 (20) act as repressors.

Recently, we reported that that Indian hedgehog (*Ihh*), a secreted ligand important for fetal development, is a crucial mediator of the regenerative acceleration triggered by ALPPS surgery (21). Despite our increasing knowledge of the interwoven biological signaling networks underlying normal and accelerated liver regeneration, comprehensive whole genome analyses are required for full recognition of the underlying pathways. The newly developed bioinformatics tool OncoFinder enables quantitative measurement of intracellular signaling pathway (ISP) activation based on whole genome expression data (22, 23). The advantage of OncoFinder over alternative tools, such as Metacore and Ingenuity Pathway Analysis (IPA), is that it quantifies the pathway activation strength (PAS) (22–24). PAS values represent the cumulative value of perturbations in a signaling pathway and serve as reliable indicators of pathological changes to the intracellular signaling machinery. The PAS value itself serves as a robust new biomarker that can distinguish between the pathway activation profiles in different tissues (23, 25).

The aim of our present study was to assess comprehensively the ISPs underlying normal and accelerated liver regeneration in two established mouse models (26, 27), and in liver tissue obtained from humans before and after the first step of ALPPS surgery. The data designate that our murine ALPPS model—despite differences—reflects ALPPS-induced accelerated liver regeneration in humans.

## Methods

### Surgery

ALPPS surgery in C57BL/6 mice (*n* = 3 for all procedures) was performed as described earlier (26). In brief, a 90% PVL was performed, leaving a 10% functional remnant consisting of the left and a part of the right middle lobe. Then, a partial 80% transection was done through the middle lobe along the demarcation line of the occluded/non-occluded parenchyma. The left lateral lobe (LLL, 25% of liver volume) was also resected to simulate the cleaning of the liver from smaller tumors as often carried out in human ALPPS (21, 26). ALPPS surgery is associated with some initial injury (serum ALT at around 5,000 U/I 1 day post-operation), which however declines over time toward zero at day 7 post-operation. Serum HMGB1, released by necrotic cells, is not elevated at any time post-operation, indicating the absence of significant necrosis as confirmed by histology on day 2. Very similar findings are observed for PVL surgery, indicating that ALPPS does not augment injury (26). Following 68% hepatectomy, injury is negligible, with ALT <100 U/I 1 day post-operation (27). Serum bilirubin is not elevated following ALPPS, PVL, or 68% hepatectomy (26, 27). Liver weight gain is clearly evident already at 4 h post ALPPS, steeply rising to reach a plateau at 24 h (with step 2—resection of ligated parts—usually performed at day 2 in mice). PVL also induces—to a lesser extent—early liver weight gain; however, a low plateau is reached already at 8 h post PVL (21). After 68% hepatectomy, liver starts to gain weight more slowly, with its strongest gains toward 48 h. This time point coincides with the hepatocellular mitotic peak, which follows cell cycle entry around 16–20 h post 68% hepatectomy (27). In contrast, hepatocytes enter the cell cycle already at 4 h post ALPPS, with a first mitotic peak at 8 h. After PVL, cell cycle entry also occurs early, but only at levels similar to transection (which does not induce regeneration), and low numbers of mitoses are observed from 12 h onwards (21). Therefore, ALPPS surgery accelerates mouse liver regeneration both in time and magnitude relative to other liver surgeries.

Standard hepatectomy (partial 68% hepatectomy) in mice was performed as described by Lehmann (27). In short, a midline incision was performed, and the liver was freed from ligaments. The pedicle of the left lobe was ligated (silk, 6/0) and resected. After cholecystectomy (Prolene, 8/0; Ethicon, Neuchatel, Switzerland), the middle lobe was ligated in 2 steps (silk 6/0) and resected. All animal experiments conformed to the Swiss Federal Animal Regulations and were approved by the Veterinary Office of Zurich. Animals aged 10–12 weeks were kept on a 12-h day/night cycle with free access to food and water. C57BL/6 mice were obtained from Envigo (Horst, The Netherlands). All animals were part of the same shipment, same age and gender, randomized, and part of the same project to ensure similar conditions.

### Tissue Specimen

Mice liver tissue was collected at different time points after surgery (as indicated throughout the manuscript). Human liver tissue specimens (biopsies) were obtained from the Department of Visceral and Transplant Surgery, University Hospital Zurich with the approval of the local ethics committees (Nr: 2015-0547, Cantonal Ethics Committee, Zurich) and written consent of all patients (for characteristics see Supplementary Data S-XI). After laparotomy and initial inspection of the abdomen, liver punch biopsies were taken of the future liver remnant (FLR) immediately before starting ALPPS step 1 [biopsy 1]. Then, partial ALPPS was performed as described earlier (7). Biopsy 2 was taken of the FLR immediately before closure of the laparotomy [end of ALPPS step 1]. All biopsies were snap frozen in liquid nitrogen and stored at −80°C. RNA isolation and sequencing was performed as described below.

### RNA Isolation and Sequencing

RNA isolation was performed using the TRIzol Method as described by the manufacturers (Life Technologies, Switzerland). An equimolar RNA pool was prepared from liver tissue and/or biopsies using DNA Column Clean-up (Qiagen, Basel, CH). Then, 1 μg of total RNA was used for library preparation according to the Illumina TruSeq stranded mRNA sample preparation protocol (Illumina). The resulting mRNA library was sequenced on an Illumina Hiseq 2,500 sequencer (Functional Genomics Center Zürich, Zürich, CH). Sequenced reads were aligned to the mouse and human (hg19) reference genome with TopHat (version 2.0.10) 27 by using the -G (GTF file of Ensembl release 75) option. Furthermore, the aligned reads were used to quantify mRNA expression by using HTSeq-count (version 0.6.1)28 with hg19 GTF (Ensembl release 75). All data have been deposited to the European Nucleotide Archive (ENA) under the accession code PRJEB15593.

### Differential Expression Analysis

Raw reads were filtered by quality >30 score through FASTX toolkit and then trimmed at 5′ and 3′ in order to remove index and adapter. Only the remaining reads were used for alignment with the human genome assembly (GRCh37), where we employed TopHat v2.0.14 (28).

### Source Datasets

The signaling pathways knowledge base developed by SABiosciences (https://www.slideshare.net/elsavonlicy/pathway-mapreferenceguide) was used to determine structures of intracellular pathways for OncoFinder (22, 23).

### Functional Annotation of Gene Expression Data

For the functional annotation of the primary gene expression data, we applied our original algorithm termed OncoFinder (22, 23). It enables calculation of the Pathway Activation Strength (PAS), a value that serves as a qualitative measure of pathway activation. Briefly, the algorithm utilizes the following formula to evaluate pathway activation:

(1) $$\begin{array}{r} PAS_{p}=\underset{n}{\sum }ARR_{np}\cdot BTIF_{n}\cdot \text{lg}\left(CNR_{n}\right) \end{array}$$

Here the *case-to-normal ratio, CNRn*, is the ratio of expression levels for a given gene (*n)* in the sample to the mean value for the control group. The Boolean flag of *BTIF* (*beyond tolerance interval flag*) equals to zero when the *CNR* value has passed simultaneously the two criteria that demark the significantly perturbed expression level from essentially normal. First, the expression level for the sample lies within the tolerance interval, where *p* > 0.05. Second, the value of *CNR* differs from 1 considerably, *CNR* 0.66 or *CNR* 1.5. The discrete value of *ARR* (*activator / repressor role*) reflects the functional role of a protein *n* in the pathway (22). The pathway-specific PAS values calculated by Oncofinder are more reliable than single gene analysis and improves the robustness of experimental transcriptomics data (23).

### Statistical Tests

The PAS values for each normal sample were obtained using the whole set of these normal samples as a reference. Distribution of PAS values was estimated, assuming its Gaussian behavior. Then, for each pathway of each sample, the probability that its PAS value comes from this estimated distribution was calculated. Additionally, *p*-values for each pathway of the entire group of samples were calculated using Wilcoxon rank-sum test. Principal component analyses were performed using the MADE4 package (29). Hierarchical clustering heatmaps with Pearson distance and average linkage were generated using heatmap.2 function from “gplots” package (30). Pearson tau correlation matrices were calculated in R 3.1.1 using a function of standard library “cor” with the default settings. Correlation diagrams were built using a function “corrplot” from the package “corrplot” sorted with respect to hierarchical clustering. Similarities between the pathways according to the content of similar genes were calculated using the Jaccard coefficient. The Jaccard coefficient measures similarity between finite sample sets and is defined as the size of the intersection divided by the size of the union of the sample sets. Venn diagrams were constructed using Venny 2.1 [http://bioinfogp.cnb.csic.es/tools/venny/].

### miRNA Target Prediction

Focusing on differentially expressed pre-miRNAs present in our datasets, we predicted their putative mRNA targets considering only experimentally validated miRNA-mRNA interactions using the Ingenuity Pathway Analysis (IPA) suite (Qiagen, Redwood City, Calif; https://www.slideshare.net/elsavonlicy/pathway-mapreferenceguide). We used miRTarBase for predicting targets of miRNAs and assessed the influence of miRNAs on ISPs using our method MiRImpact (31). Among all miRNA-targeted mRNAs, only genes having at least 10 reads (read count ≥10) were considered true targets for differentially expressed miRNAs in BSM cells.

### Gene Set Variation Analysis of Mitotic Cell Cycle Signatures

To compare the kinetics of the expression of mitotic cell cycle genes between ALPPS and standard hepatectomy, gene signatures for different phases of the mitotic cell cycle were selected from the MSigDB and GSKB online databases. For access to the GSKB database, the gskb R package (version 1.10.0) was used. Individual-sample gene set enrichment scores were calculated using Gene Set Variation Analysis with the GSVA R package, version 1.26.0. All gene set enrichment analyses were performed within the R statistical programming environment, version 3.4.3.

## Results

### ALPPS Surgery Induced an Earlier Cell Cycle Entry

**Figure 1:**
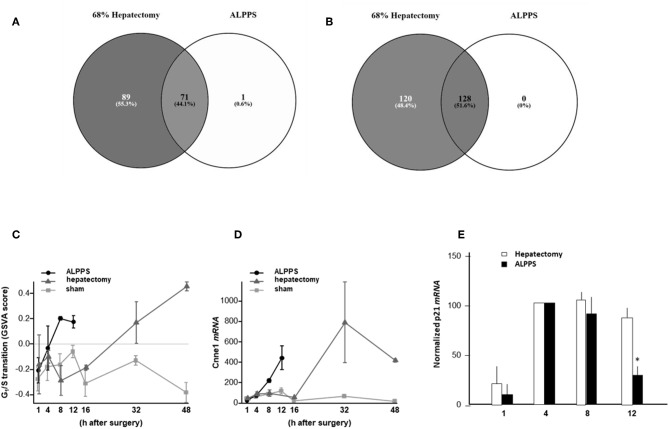
Venn diagrams showing the total of unique and common ISPs after 68% hepatectomy and ALPPS surgery. **(A)** Unique and common ISPs activated/repressed 4 h after surgery. **(B)** Common ISPs activated/repressed 8 h after surgery (For details see: Table 2). **(C)** Per-sample enrichment scores for genes involved in the G1/S transition phase of the mitotic cell cycle (the GO_BP_MM_G1_S_TRANSITION_OF_MITOTIC_CELL_CYCLE signature from the GSKB database), calculated using gene set variation analysis (GSVA). **(D)** Cyclin E1 mRNA (Ccne1) mRNA expression. **(E)** Expression of p21 mRNA after ALPPS and 68% hepatectomy (as indicated); *p < 0.005.

To elucidate the molecular pathways responsible for accelerated liver regeneration, hepatic RNA isolated at several time points after ALPPS, PVL, transection, LLLx, and sham operation was deep-sequenced. Pathway activation strength (PAS) profiles were established using the normalized gene expression levels of liver-expressed genes with the OncoFinder algorithm. First, we analyzed the activation status of 378 ISPs. Using the PAS values, we built hierarchical clustering heat maps with Euclidean distance and average linkage for all groups (For heatmaps see: Supplementary Data S-I). We identified the common and unique ISPs for ALPPS and normal liver regeneration as induced by standard hepatectomy. As shown in Figure 1A, 160 and 72 ISPs were significantly affected 4 h after 68% hepatectomy and ALPPS, respectively. While 89 ISPs were unique for standard hepatectomy, only the Interferon main pathway was specific for the ALPPS procedure (PAS:−0.479740209). Likewise, 120 ISPs were shared between the two procedures, with no unique ISPs identified for ALPPS 8 h after surgery (Figure 1B; Supplementary Data S-II). These data indicate that the two procedures predominantly differ quantitatively, not qualitatively. The earlier activation of the *Cell Cycle-pathway (metaphase-anaphase)* 8 and 12 h after ALPPS surgery (Supplementary Data S-III) signifies the initiation of chromosomal replication and segregation during cell divisions. It associated with a much earlier cell cycle entrance relative to standard hepatectomy (Figure 1C; Supplementary Data S-IV), an earlier increased expression of cyclin E1 (Figure 1D) and a decline of the cell cycle inhibitor p21 mRNA (Figure 1E). To identify pathways that are most significantly different between ALPPS and 68% hepatectomy, we performed a more stringent analysis only including those pathways having PAS values ≥0.1. We subsequently obtained 47 and 69 affected pathways after 4 and 8 h, respectively. Table 1 displays the PAS values of these ISPs, demonstrating that the ALPPS procedure affects the same ISPs as standard hepatectomy, but to a significantly higher extent. Of particular interest are the highly significant increased PAS values of the *ATM Pathway (G2_M Checkpoint Arrest)*, the *HGF Pathway (Cell cycle progression)*, the *EMT-associated hypoxia pathways*, and the *IL-10 Pathway (Stability determination)*. In addition, the three branches of the *TGF*β *Pathway* and 10 branches of the *ILK Pathway* were significantly increased 4 h after ALPPS surgery (Table 1). The same set of ISPs were retrieved comparing ALPPS-specific pathways to those significantly affected 32 and 48 h after 68% hepatectomy (Supplementary Data S-III).

**Table 1:** Comparing the intracellular signaling pathways (ISPs) of standard (68%) hepatectomy and ALPPS 4 h after surgery.

**Intracellular signaling pathway**	**PAS (68% Hx)**	**PAS (ALPPS)**
Androgen receptor pathway (gonadotropin regulation)	0.080962033	0.170591759
Androgen receptor pathway (histone modification)	0.080962033	0.170591759
Androgen receptor pathway (prostate differentiation and development)	0.080962033	0.170591759
Androgen receptor pathway (sexual differentiation and sexual maturation at puberty)	0.080962033	0.170591759
ATM main pathway	0.069585724	0.164331506
ATM pathway (G2_M checkpoint arrest)	0.192334187	0.758265977
BRCA 1 main pathway	−0.01618013	−0.173732307
EGFR main pathway	0.062796782	0.101805817
ErbB family main pathway	0.05751811	0.172579526
GPCR pathway (gene expression)	0.068563364	0.123092979
HGF main pathway	0.062203501	0.112692194
HGF pathway (cell cycle progression)	0.337396472	0.546430817
Hypoxia pathway EMT 1	0.084001749	0.691549538
Hypoxia pathway EMT 2	0.084001749	0.691549538
Hypoxia pathway EMT 3	0.084001749	0.691549538
Hypoxia pathway EMT 4	0.084001749	0.691549538
ILK Main Pathway	0.074326641	0.17275502
ILK Pathway (Apoptosis)	0.091947966	0.169660241
ILK Pathway (Cell adhesion, cell motility, opsonization)	0.095965334	0.218655438
ILK pathway (cell cycle proliferation)	0.08622972	0.180086957
ILK pathway (cell migration, retraction)	0.094442075	0.206157845
ILK pathway (cell motility)	0.080839955	0.19368415
ILK pathway (cytoskeletal reorganization)	0.115000716	0.249836663
ILK pathway (G2-phase arrest)	0.08622972	0.180086957
ILK pathway (induced cell proliferation)	0.197724934	0.186502548
ILK pathway (regulation of intermediate filaments)	0.106090655	0.23979623
ILK pathway (regulation of junction assembly of desmosomes)	0.095197611	0.216906194
ILK pathway (wound healing)	0.095197611	0.224489964
IL-10 pathway (stability determination)	0.053478054	1.848219244
IL-2 main pathway	0.017026437	0.113993181
lntegrin signaling main pathway	0.067439974	0.145964253
JNK pathway (apoptosis, inflammation, tumorigenesis, cell migration)	0.079482132	0.207123801
JNK pathway (insulin signaling)	−0.07241052	−0.426061537
MAPK signaling main pathway	0.0519986	0.108187021
MAPK signaling pathway (cell survival, inflammation, apoptosis, osmoregulation)	0.212081896	0.229612073
MAPK signaling pathway (gene expression)	0.091194477	0.149905425
mTOR pathway (actin organization)	0.059587334	0.128667337
p53 signaling (negative) main pathway	0.084470457	0.14626007
PAK main pathway	0.034655373	0.117954615
SMAD (negative) main pathway	0.139663741	0.315152085
SMAD (positive) main pathway	0.139663741	0.315152085
TGF beta pathway (SnON degradation)	0.166857414	0.576950547
TGF beta pathway (tumorigenesis)	0.238367734	0.512365322
TGF beta pathway (tumor suppression)	0.238367734	0.512365322
TNF (positive) pathway (gene expression, cell survival)	0.157317821	0.326862624
VEGF pathway (actin reorganization)	0.114882119	0.070794654

*Only ISPs with PAS values ≥0.1 are shown. The most significantly affected ISPs (PAS values ≥ 0.5) are highlighted*.

### Dissecting ALPPS-Induced Pathway Activation Profiles

**Figure 2:**
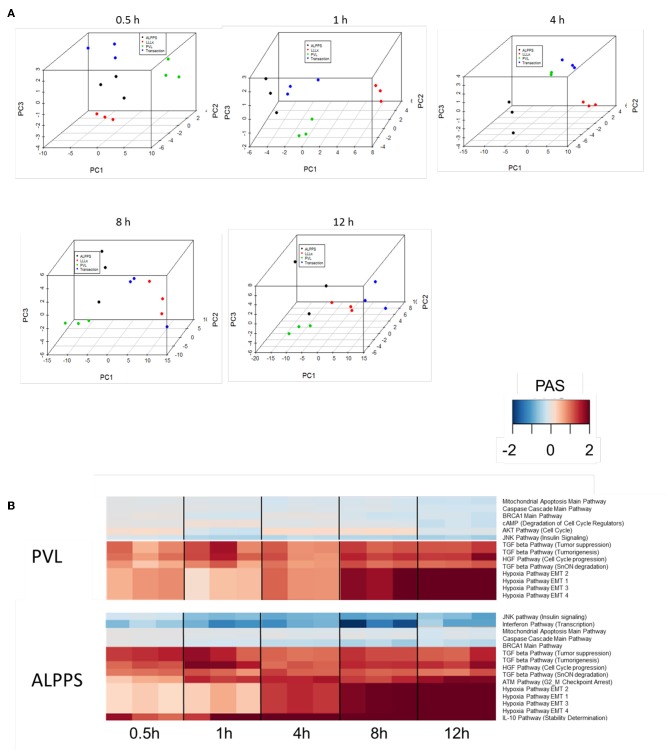
**(A)** Results of the principal component analysis 0.5, 1, 4, 8, and 12 h after surgery. Green dots, left lateral liver lobectomy (LLLx); Blue dots, Transection; Red dots, portal vein ligation (PVL); Black dots, ALPPS. **(B)** Kinetics of the most significant PVL- and ALPPS-induced ISPs over time.

**Figure 3:**
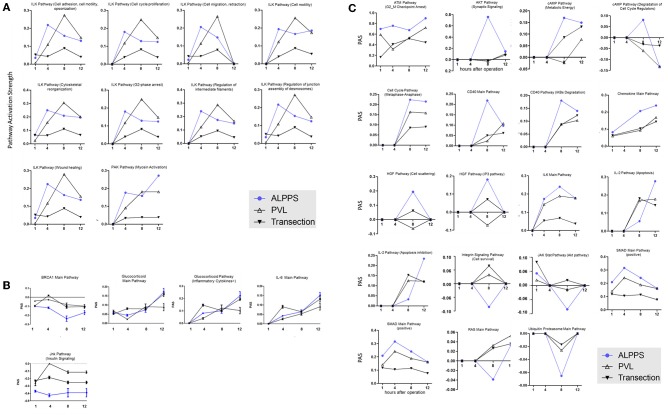
PAS values for common ISPs as a function of time after Transection-, PVL-, and ALPPS-surgery. **(A)** Relative to PVL and Transection, 10 ISPs presented with a significantly increased PAS value 4 h after ALPPS surgery. **(B)** Significantly decreased ISPs 4 h after ALPPS surgery (relative to either PVL or transection). **(C)** ISPs with a unique profile after ALPPS surgery.

To further elucidate the molecular pathways responsible for accelerated liver regeneration, hepatic RNA isolated during the first 12 h after ALPPS, PVL, transection, LLLx, and sham operation was analyzed. Unsupervised hierarchical clustering separated the samples into two early groups (≤ 4 h post OP) and two late groups (≥8 h post OP). The dendrogram presentation revealed that ALPPS samples isolated 4 h after surgery grouped together with all late samples of PVL and ALPPS (≥8 h post OP), indicating an accelerated biological response after the ALPPS procedure (Supplementary data S-XII). Principal Component Analyses confirmed that the major dissimilarities between surgical procedures occurred 4 h past surgery (data not shown). In accordance with the expression data, the most distinctive differences between the groups were observed 4 h after the surgical procedures were performed. Principal Component Analyses also confirmed that the major dissimilarities between surgical procedures occurred 4 h after surgery (Figure 2A). The observation that the samples of the same surgical procedures hardly segregate (colored dots) underlines the excellent reproducibility of the different surgical procedures. The PAS data showed that ALPPS surgery significantly affected 72 ISPs, whereas PVL and transection affected 88 and 46 ISPs, respectively. The kinetics of the most significant PVL- and ALPPS-induced ISPs are presented in Figure 2B. Next, we constructed Venn diagrams to find common and distinctive ISPs for ALPPS, PVL, and transection. As shown in Table 2, all surgical procedures involve unique ISPs. After transection, three unique ISPs are affected: the *Glucocorticoid Receptor Pathway (Gene expression)*, the *Growth Hormone Pathway (Gene expression)*, and the *IL-2 Pathway (Actin reorganization)*. The ALPPS procedure was marked by three unique upregulated pathways [*IGF1R Signaling Pathway (Cell survival)*, the *ILK Pathway (Induced cell proliferation)*, the *IL-10 Pathway (Stability determination)*, and the downregulation of the *Interferon Pathway (Transcription)*], whereas the PVL procedure was by twenty unique ISPs (For all ISPs see: Supplementary Data S-V; For over-time heatmaps see: Supplementary Data S-VI). Remarkably, the ISPs affected by ALPPS are not just the sum of the ISPs affected by PVL plus transection, indicating that the ALPPS procedure is synergistic rather than additive. To define time-dependent ALPPS-specific ISP signatures indicated by the shifted transcriptional landscape, we plotted the PAS values as a function of time. Four hours after surgery, ten ISPs presented with a significantly increased PAS value (Figure 3A), five ISPs were significantly decreased (Figure 3B), whereas 19 ISPs demonstrated unique profiles (Figure 3C).

**Table 2:** Activated and repressed ISPs unique for Transection, ALPPS, and PVL 4 h after surgery.

**ISPs exclusively in ALPPS**	**PAS**
IGF1R signaling pathway (cell survival)	0.09677963
ILK pathway (induced cell proliferation)	0.18650255
IL-10 pathway (stability determination)	1.84821924
Interferon pathway (transcription)	−0.47974021
**ISPs exclusively in PVL**	
AKT pathway (cell cycle)	0.09431465
Androgen receptor pathway (cell survival and cell growth)	0.1243162
ATM pathway (cell survival)	0.05295231
cAMP main pathway	0.05587309
cAMP pathway (degradation of cell cycle regulators)	−0.04355851
Erythropoeitin main pathway	0.04643793
Hedgehog pathway (repression of Hh, BMP)	0.08050423
HGF Pathway (Anoikis)	0.20617418
HIF1-Alpha main pathway	0.06045947
HIF1Alpha pathway (gene expression)	0.14085828
HIF1Alpha pathway (NOS pathway)	0.16252878
HIF1Alpha pathway (Pyruvate)	0.14085828
HIF1Alpha pathway (VEGF pathway)	0.13205464
IL-10 main pathway	0.27795708
lntegrin signaling pathway (cytoskeleton contraction integrin modulation cell invasion and migration)	0.12105071
Interferon main pathway	0.03386061
IP3 main pathway	0.03666389
MAPK family pathway (gene Expression)	0.03057693
RAS main pathway	0.04229486
TGF beta pathway (post-transcriptional G1 arrest)	0.13784324
**ISPs exclusively in transection**	
Glucocorticoid receptor pathway (gene expression)	−0.01557421
Growth hormone pathway (gene expression)	0.02746121
IL-2 pathway (actin reorganization)	0.4806433

*Positive PAS, activated ISPs; Negative PAS values, repressed ISPs. ISPs with PAS*.

*The most significantly affected ISPs (up and down) are highlighted*.

### Identification of ALPPS-Specific microRNAs

**Figure 4:**
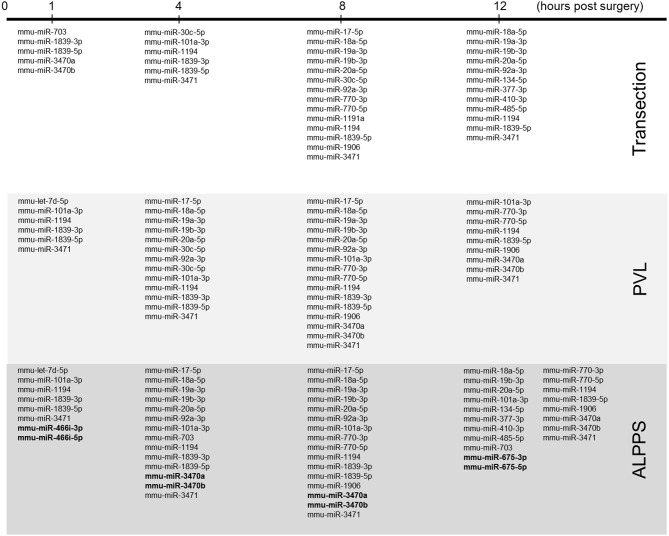
miRNA expression patterns as a function of time after surgery (Transection, PVL and ALPPS). miRNAs specific for ALPPS, as well as miRNAs expressed at an earlier time point, are highlighted in bold.

Our dataset contained 73 differentially expressed long non-coding (lnc)RNA transcripts, of which 60 were identified as pre-miRNAs (Supplementary Data S-VII). Assuming pre-miRNA sequences as the precursors of mature miRNAs, we further analyzed their expression patterns and effects on ISPs. After removal of possible miRNA-targeted mRNA transcripts not expressed in the liver, using miRTarBase software we identified 28 known miRNAs, which together target 2,003 experimentally validated mRNAs (Supplementary Data S-VIII). How the distribution of miRNA expression patterns changes over time after ALPPS, PVL, and transection is presented in Figure 4. We identified six miRNAs, which may serve prominent roles in the biological processes involved in accelerated liver regeneration. *Mmu-miR 466i-3p* and *mmu-miR 466i-5p* were exclusively expressed 1 h after ALPPS surgery, whereas *mmu-miR 675-3p* and *mmu-miR 675-5p* were exclusively observed 12 h after ALPPS surgery. In addition, *mmu-miR 3470a* and *mmu-miR-3470b* were detected 4 h after ALPPS surgery, whereas they first appeared in the PVL samples 8 h post-surgery.

### ISPs Analysis in Human ALPPS

**Figure 5:**
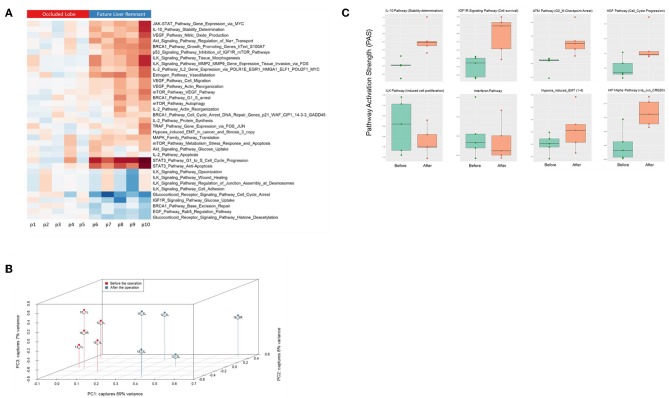
**(A)** Hierarchical clustering heatmap based on the analysis of 374 intracellular signaling pathway activation profiles in human liver tissue (occluded lobe compared to future liver remnant). Red, positive PAS scores; blue, negative PAS scores for a given pathway in a given sample. **(B)** Results of the principal component analysis before and after the first step of ALPPS surgery of the human liver samples. **(C)** PAS values of ALPPS-specific pathways identified in our murine ALPPS model compared to those obtained with human liver samples.

Finally, we assessed to what extent the observations with our mouse model of ALPPS reflect the biology of accelerated liver regeneration in humans. Considering the difficulty of sampling humans' hepatic samples, we isolated RNA from liver biopsies taken just before initiating and concluding the first stage of the ALPPS procedure (4–5 h apart; *n* = 5). Again, we built ISPs with normalized RNAseq expression data using the OncoFinder software and plotted the PAS values of the 35 most affected ISPs (Figure 5A). The heatmap clearly demarcated two groups with similar ISP activation patterns representing the biopsies taken from the occluded lobe and the FLR, respectively. Principal Component Analyses confirmed the major dissimilarities between the two groups (Figure 5B). The five most activated ISPs in the FLR were identified as the *STAT3 Pathway* [*G1_to_S_Cell_Cycle_Progression* and the *STAT3 Pathway (Anti-Apoptosis)*], the *JAK-STAT Pathway (Gene_Expression_via_MYC)*, the *IL-10 Pathway (Stability determination)*, the *Estrogen Pathway (Vasodilatation)*, and the *Akt-Signaling Pathway (Regulation of Na*^+^ *transport)*. The five most repressed pathways were identified as the *Glucocorticoid Receptor Signaling Pathway (Cell_Cycle_Arrest)*, the *Glucocorticoid Receptor Signaling Pathway (Histone_Deacetylation)*, the *EGF Pathway (Rab5_Regulation_Pathway)*, the *IGF1R Signaling Pathway (Glucose_Uptake)*, and the *BRCA1 Pathway (Base_Excision_Repair)*. A comprehensive list of all affected pathways, including the PAS values, can be found in the Supplementary Data (S-IX). Finally, we compared the ALPPS-specific pathways identified in our mouse model to those obtained with human liver samples. As demonstrated in Figure 5C, two of the four ALPPS-specific ISPs of mice are also upregulated in humans, immediately after ALPPS surgery step 1 [*IGF1R Signaling Pathway (Cell survival)* and the *IL-10 Pathway (Stability determination)*]. In contrast, the *ILK Pathway (Induced cell proliferation)* and the *IFN main pathway* were not significantly affected. In the human samples, we also retrieved the ISPs that were highly and significantly enriched in ALPPS when compared to standard hepatectomy, including the *ATM Pathway (G2_M Checkpoint Arrest)*, the *HGF Pathway (Cell cycle progression)*, the *EMT-associated hypoxia pathways*, and the ensuing activation of the *HIF1Alpha pathway via Jun_CREB3* (Figure 5C).

## Discussion

The novel two-staged hepatectomy ALPPS, which combines PVL plus a parenchymal liver transection, has gained increasing interest among liver surgeons. Its importance lies in the observation that ALPPS induces accelerated liver hypertrophy, so that hepatectomy can be completed within a very short time frame (7–10 days post stage 1). Previously, we reported that the *Ihh* gene is one of the 50 most upregulated genes 4 h after ALPPS surgery in mice and present in serum of patients shortly after the ALPPS procedure (21). We here observed that *Hedgehog signaling* is not unique to ALPPS surgery, since it is also activated during normal liver regeneration after 68% hepatectomy—a finding in accordance with earlier studies (32). Importantly, however, hedgehog signaling after 68% hepatectomy was induced primarily through *shh*, while *ihh* is the dominant morphogen after ALPPS (21).

Our current data demonstrate that ALPPS surgery is unique in that it induced an earlier activation of the cell cycle, signified by increased expression of cyclin E1 and a decline of the cell cycle inhibitor p21. In search of an ALPPS-specific signature, we observed four ISPs that demarcated the ALPPS procedure from PVL and transection. After ALPPS surgery, the *IGF1R Signaling Pathway (Cell survival)*, the *ILK Pathway (Induced cell proliferation)*, and the *IL-10 Pathway (Stability determination)* were significantly enriched, whereas the activity of the *Interferon Pathway (Transcription)* was reduced. The *IGF1R signaling pathway* for cell survival has been associated with early wound healing and liver cell proliferation via the IGF1R/IRS1/ERK axis and activation of the cyclins A1 and D1. Disrupting hepatic IGF1R signaling has been shown to significantly impair hepatocyte proliferation in a mouse model of liver regeneration, and IGFBP-1 null mutants show abnormal liver regeneration (33). Increased IGF1R signaling may thus be reflecting an accelerated regeneration process. The integrin-linked kinase (ILK) is a protein involved in transmitting extracellular matrix signals and has been associated with the termination of liver regeneration. Mice lacking hepatic ILK expression cannot appropriately complete the liver regeneration process, and DNA synthesis in such mice is prolonged (34). Our data now indicate that a specific branch of the ILK pathway is also crucial to the early phase of ALPPS-induced accelerated liver regeneration, possibly contributing to the early matrix production. ALPPS surgery is also unique in that it decreased the *IFN-Main Pathway* and concomitantly induced a branch of the *IL10 Pathway*. Earlier, it was reported that IFN-γ deficiency in mice enhanced liver regeneration responses (35). In hepatocytes, IFN-γ activates Stat-mediated signaling, leading to the activation of p53, which together form transcriptionally active protein complexes to induce the cell cycle inhibitor p21 (36). The reduced IFN signaling may thus denote accelerated cell cycle progression due to diminished p21 activity. Further, the dampening of IFN signaling implies a role for IFN-γ producing cells, such as T lymphocytes, NK and NKT cells. In response to surgery-inflicted injury, these cells may receive signals not to enter the liver or to silence their activity. The activation of NK and NKT cells clearly impeded liver regeneration, which also involved IFN-γ mediated STAT1 signaling (35). The increased activity of the *IL10 pathway* is indeed suggestive of such scenarios, since it functions to diminish inflammatory responses, hence preparing an environment suitable for augmented regeneration as observed after ALPPS surgery.

In addition to these ALPPS-specific signaling pathways, we observed 10 ISPs activated at an earlier time point, paralleling the hepatic mRNA levels peaking at 4 h post ALPPS surgery. With nine distinctive branches, the ILK pathways predominated these shifted ISPs. The integrin-linked kinase (ILK) is a PI3-kinase-dependent, serine/threonine protein kinase that interacts with the cytoplasmic domains of both β1 and β3 integrins, possesses kinase activity, and controls an assortment of biological activities (37). The wide-ranging functionality of ILK pathways in numerous processes related to cell proliferation and tissue remodeling (see: Figure 3A), as well as their early and increased PAS values after ALPPS surgery, picture ILK as a plausible key regulator of accelerated liver regeneration. The earlier activation of one branch of the *PAK pathway (myosin activation)*, which is also a characteristic feature of ALPPS, may reflect the above findings, since ILK and PAK1 cross-regulate each other and together regulate cytoskeletal dynamics. A function for PAKs has also been established in cell cycle progression in cancers, in which PAKs are overexpressed or hyper-activated (38).

Comparing standard hepatectomy with ALPPS surgery, we demonstrated that 4 h after surgery the two procedures predominantly differ quantitatively, not qualitatively. The only ALPPS-specific signature was—as described above—the suppression of the *IFN Main Pathway*, which clearly demarcated ALPPS from standard hepatectomy. The majority of the affected ISPs demonstrated a low but detectable activity after normal liver regeneration. After ALPPS surgery, however, the activities of these ISPs were dramatically increased. Of particular interest are the four branches of the hypoxia-induced pathway, which are 8.2-fold higher in ALPPS than in standard hepatectomy. Hypoxia signaling has been implicated in regulating the transition that is necessary to produce the extracellular matrix but also to initiate the regenerative capacity of EMT-like liver cells (39). Although it has been proposed that *in situ* liver partition contributes to hypoxia (40), our data explicitly demonstrate that ligation of the portal vein suffices to induce the hypoxia pathways (Supplementary Date S-I). Hypoxia of the FLR is thus an immediate early event after PVL, probably due to an excess of oxygen-poor blood from the portal vein, and a major trigger for the accelerated ALPPS-induced regenerative response.

The importance of hypoxia as a driver of liver regeneration is increasingly being recognized. Although HIFs have traditionally been in the focus, HIF1A has been reported not to react toward hypoxia in the liver. Interestingly, HIF1A's cellular location was associated with peroxisomes rather than the nucleus upon exposure of hepatocytes to hypoxia (41). Conceivably, early changes due to the altered portal flow may provide triggers (such as activation of Kupffer cells, or the re-organization of matrix) for regeneration but so far are unproven. When the parenchyma expands, however, hypoxia develops in analogy to a growing tumor. After 70%HX, hypoxia leads to HIF2A (but not HIF1A) activation, which promotes hepatocyte mitosis and induces VEGF production for the later angiogenic phase (42). Thus, hypoxia imprints timely order on regeneration, with the start of the angiogenic phase coupled to the successful completion of hepatocyte division. Recently, unspecific activation of HIFs by ethyl-3,4-dihydroxybenzoate was shown to accelerate PVL regeneration to ALPPS levels but had no discernible effect on liver metastases (40, 43). Induction of the HIF pathway may thus be a first clinical approach to improving liver regeneration after 68% hepatectomy or PVL.

Further, three branches of the *TGF*β*-pathway*, the *IL10-Pathway* (stability determination), and the *ATM-pathway* (G2-M_Checkpoint_Arrest) are significantly enriched after the ALPPS procedure. At the molecular level, one of the key mediators of regenerative responses is the secreted cytokine transforming growth factor-β (TGFβ) (44). The same branch of the *IL-10-pathway (stability_determination)* is again recovered. Relative to normal hepatectomy, the activity of the ATM main pathway and its specific branch for G2-M checkpoint arrest were significantly increased 4 h post ALPPS surgery. The G2-M DNA damage checkpoint is an important cell cycle checkpoint ensuring that cells do not initiate mitosis before repairing damaged DNA after replication. Cells that have a defective G2-M checkpoint enter mitosis before repairing their DNA, leading to death after cell division.

Our comprehensive analysis demonstrated that the distinct surgical procedures underlying ALPPS surgery—PVL and transection—are discernible by a distinctive set of activated and repressed ISPs. It also revealed that ALPPS surgery is not merely an addition of the pathways induced by PVL plus Transection. Although the molecular mechanism for this synergism is currently unclear, it might be explained by the presence and involvement of several unique ALPPS-specific microRNAs. MicroRNAs (miRNAs) are short, single-stranded RNAs that modify gene expression at the post-transcriptional level and are heavily involved in the spatiotemporal control of gene expression during the entire process of liver regeneration. The number, nature and level of expressed miRNAs profoundly depends on the organism studied, the activation status and microenvironment of cells, as well as other unidentified factors. In rats and mice, several miRNAs have been linked to liver regeneration, including miR19a, miR21, and miR214 (45). Here, we recognized 28 precursor transcripts of 28 miRNA present after liver surgery (Transection, PVL, and ALPPS) and identified four that were uniquely and specifically enriched after ALPPS surgery. *Mmu-miR 466i-3p* and *mmu-miR 466i-5p* were uniquely expressed immediately after ALPPS surgery, indicating they may underlie the initiation of the accelerated regeneration process by deactivating the mRNAs of cell cycle inhibitors. Indeed, *miR 466i-3p* and *mmu-miR 466i-5p* intervene with many targets of ISPs that keep the cells committed and prevent them from preparing for cell cycle entry, including the *PAK Pathway (Actin_Organization)*, the *p38 pathway (Actin-Cytoskeleton_reorganization)*, the *MAPK signaling pathways*, and the *Jnk Pathway (Gene expression Apoptosis Inflammation_Tumorigenesis_Cell-Migration)* (see Supplementary Data S-X). Likewise, *mmu-miR 3470a* and *mmu-miR-3470b* were uniquely detected 4 h after the ALPPS procedure and accordingly coincided with the ALPPS-specific shift described earlier (21). *Mmu-miR 675-3p* and *mmu-miR 675-5p*, both exclusively observed 12 h after ALPPS surgery, exert a narrower range of biological activities, mainly silencing anti-proliferative pathways. Indeed, *miR-675* and its precursor long-non-coding RNA, H19, contribute to increased proliferation, and apoptosis inhibition (46). These ALPPS-specific miRNAs may cooperate so that the earlier described ALPPS-specific ISPs become prevalent—unhindered from competing inhibitory signals and/or ISPs. Also of interest is the observation that six of the 28 detected miRNAs target the PTEN gene (*mmu-miR-17-5p, mmu-miR-18a-5p, mmu-miR-19a-3p, mmu-miR-19b-3p, mmu-miR-20a-5p, and mmu-miR-410-3p*), thus enhancing PI3-Kinase/Akt signaling and fatty acid metabolism. The downregulation of PTEN may fuel liver growth after hepatectomy due to increased β-oxidation (47).

Comparing the human data to the murine data, we observed that they have several ISPs in common and that the direction of change is the same, indicating our ALPPS model reflects the major changes of accelerated regeneration in humans at the molecular level. That not all ALPPS-specific ISPs observed in mice are retrieved in human liver tissue may reflect distinct kinetics of the liver regeneration process: sufficient regeneration in our mice model is achieved 2 days post-surgery, while it takes 7–10 days in humans. Still, the data signify that our murine ALPPS model—despite differences—may be useful to gain insight into the molecular background underlying ALPPS-induced accelerated liver regeneration in humans. Considering the close match between ISPs in mice and humans, deeper exploration of our ALPPS model may disclose essential leads to the development of potential therapeutic strategies targeting specific ISPs, in particular those that counteract responses of the immune system.

We realize that our results require further biological validation, since RNAseq data do not give insights into protein expression changes or activities of proteins belonging to a specific pathway. Still, our study provides a comprehensive framework of the signaling pathways involved in normal and accelerated liver regeneration, which is now available for further exploration.

## Data Availability Statement

The datasets generated for this study can be found in the ENA, PRJEB15593.

## Ethics Statement

The studies involving human participants were reviewed and approved by Cantonal Ethics Committee, Stampfenbachstrasse 121, 8090 Zürich Switzerland. The patients/participants provided their written informed consent to participate in this study. The animal study was reviewed and approved by Veterinary Office Zürich, Zollstrasse 20 8090 Zürich, Switzerland.

## Author Contributions

PB: experimental design, fund raising, data analysis, and writing. MSc: experimental design, patients, and writing. LF, MSo, and AB: data analysis. ML and EK: animals and experiments. RG and BH: fund raising, consultancy, and animal studies. P-AC: fund raising, study design, patients, and consultancy.

### Conflict of Interest

AB was employed by company OmicsWay Corp., Walnut, CA, United States and Oncobox ltd., Moscow, Russia. MSo was employed by company OmicsWay Corp., Walnut, CA, United States. The remaining authors declare that the research was conducted in the absence of any commercial or financial relationships that could be construed as a potential conflict of interest.

## Abbreviations

ALPPS: Associating Liver Partition and Portal Vein Ligation Surgery
LLLx: left lateral lobe resection
PAS: pathway activation strength
PVL: portal vein ligation
Tx: parenchymal transection
TSH: two-stage hepatectomy
FLR: functional liver remnant.
