# Supplementary material for: Exploration of the Transcriptional Landscape of ALPPS Reveals the Pathways of Accelerated Liver Regeneration
Source: Front Oncol. 2019 Nov 19;9:1206. doi: 10.3389/fonc.2019.01206 (PMC6882302; doi:10.3389/fonc.2019.01206)

**Heatmap 1h post surgery: 30 differentially expressed ISPs**


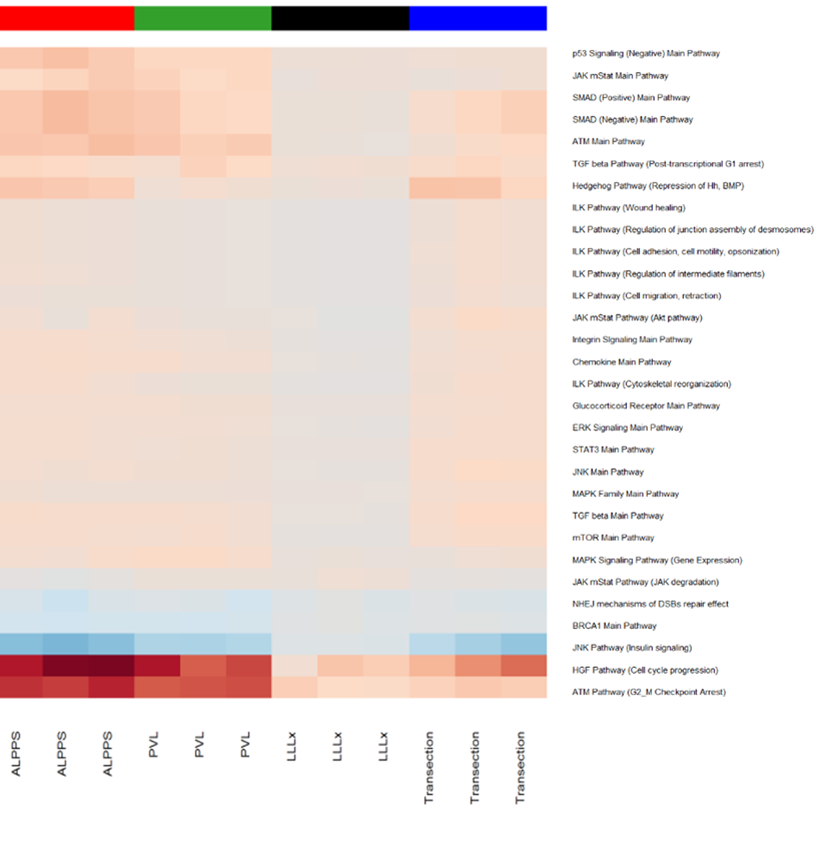


**Heatmap 4h post surgery: 67 differentially regulated ISPs**


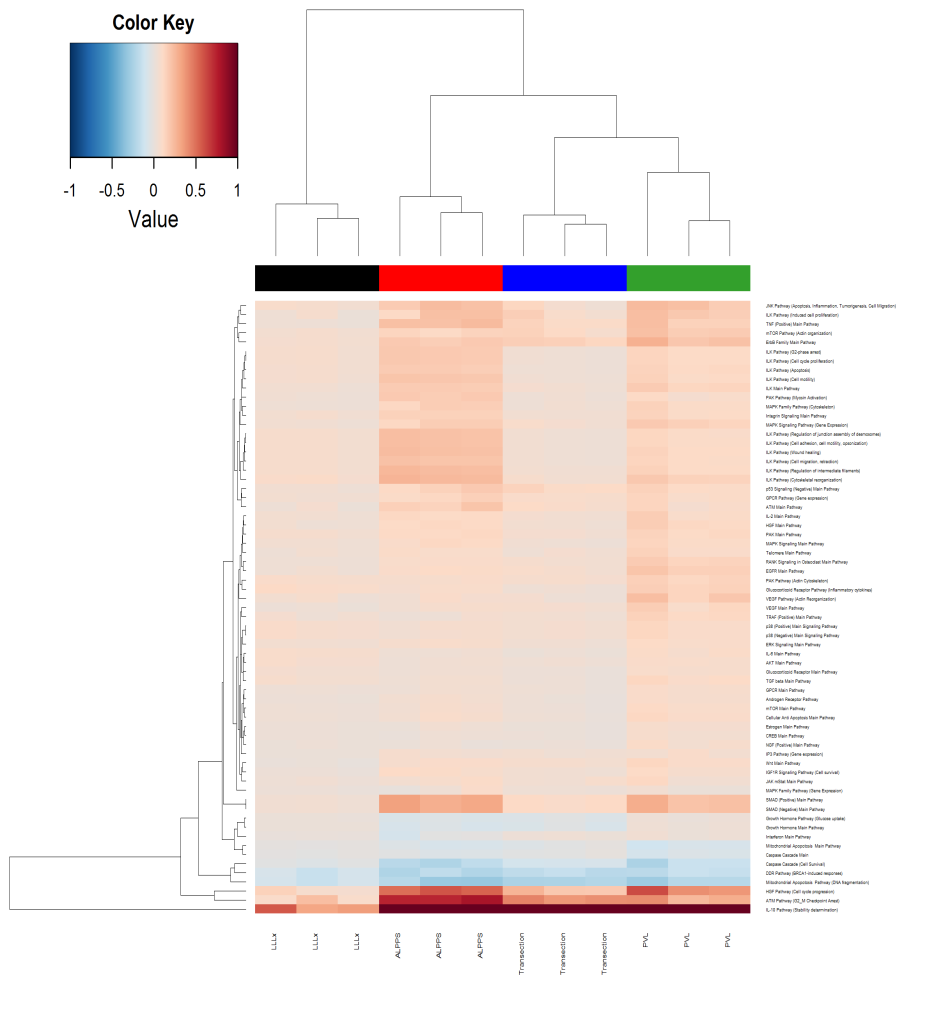

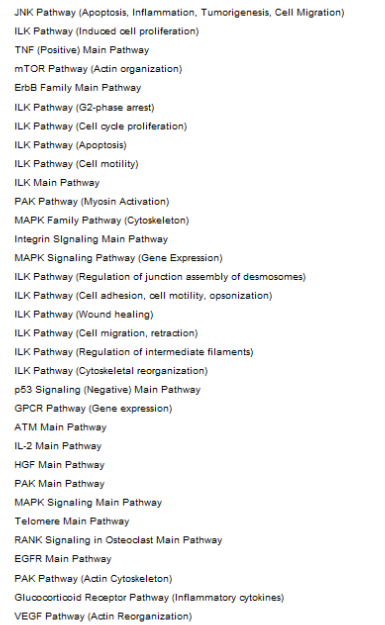

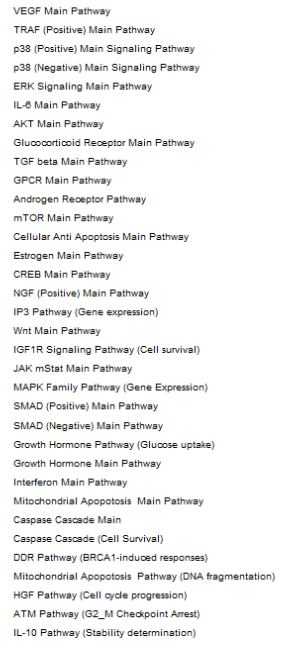


**Heatmap 8h post surgery: 128 differentially regulated ISPs**


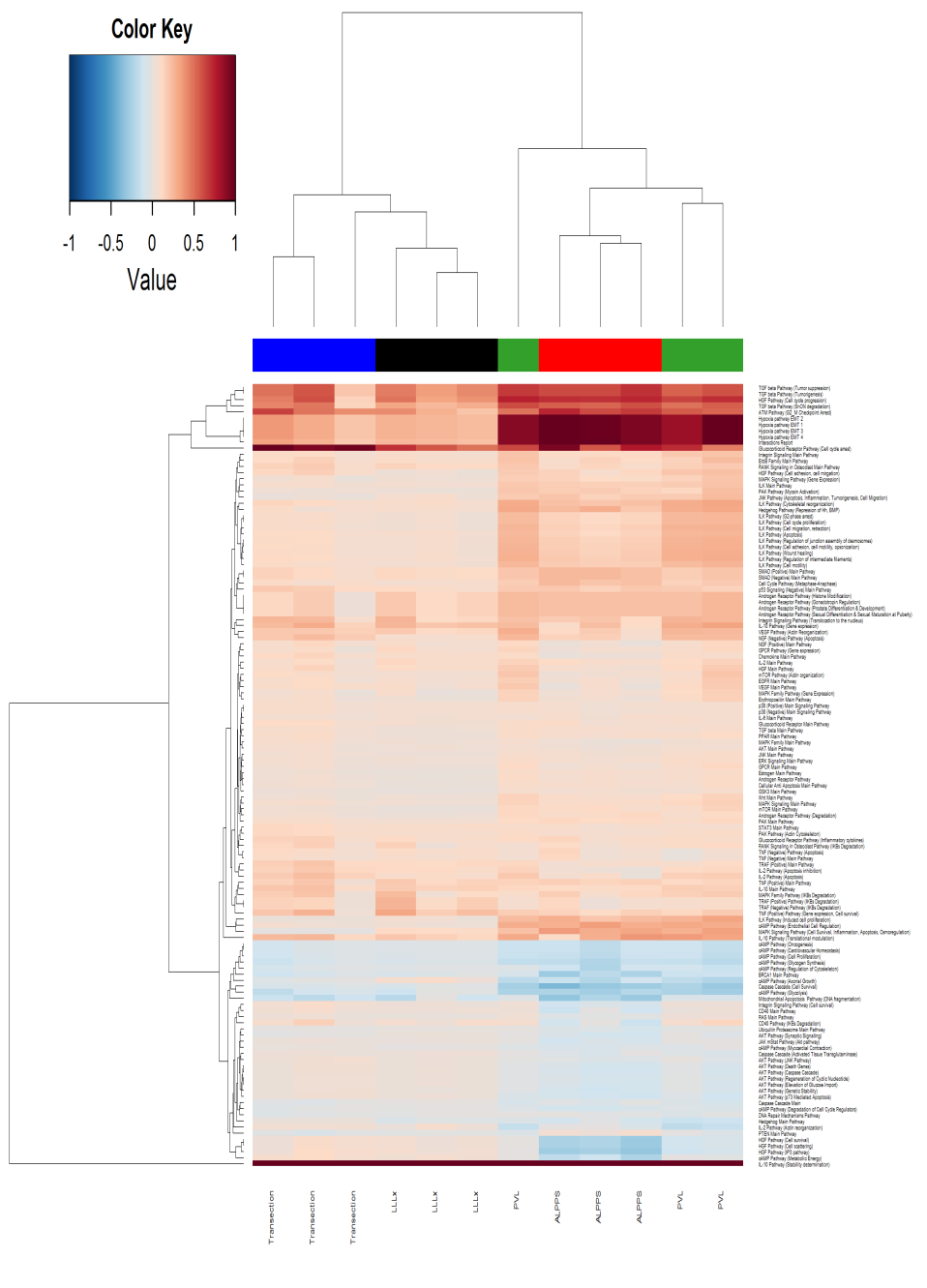

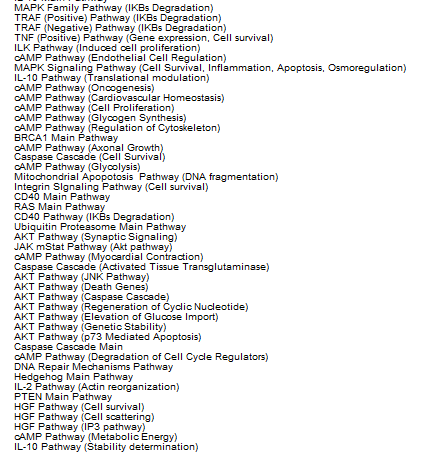

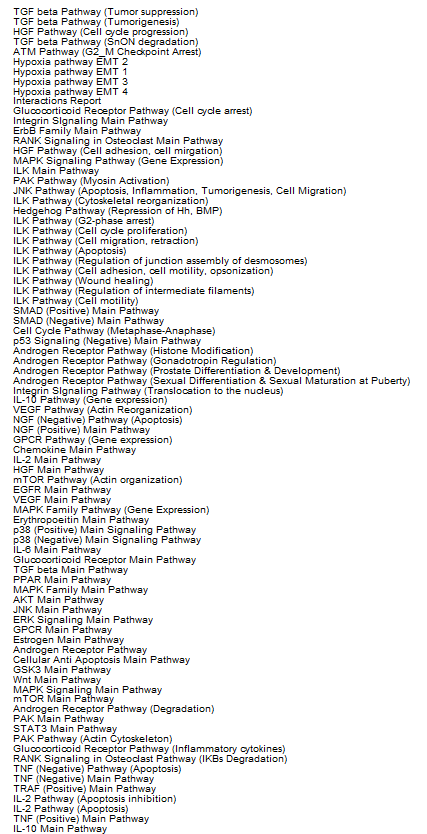


**Heatmap 12h post surgery: 137 differentially expressed pathways**


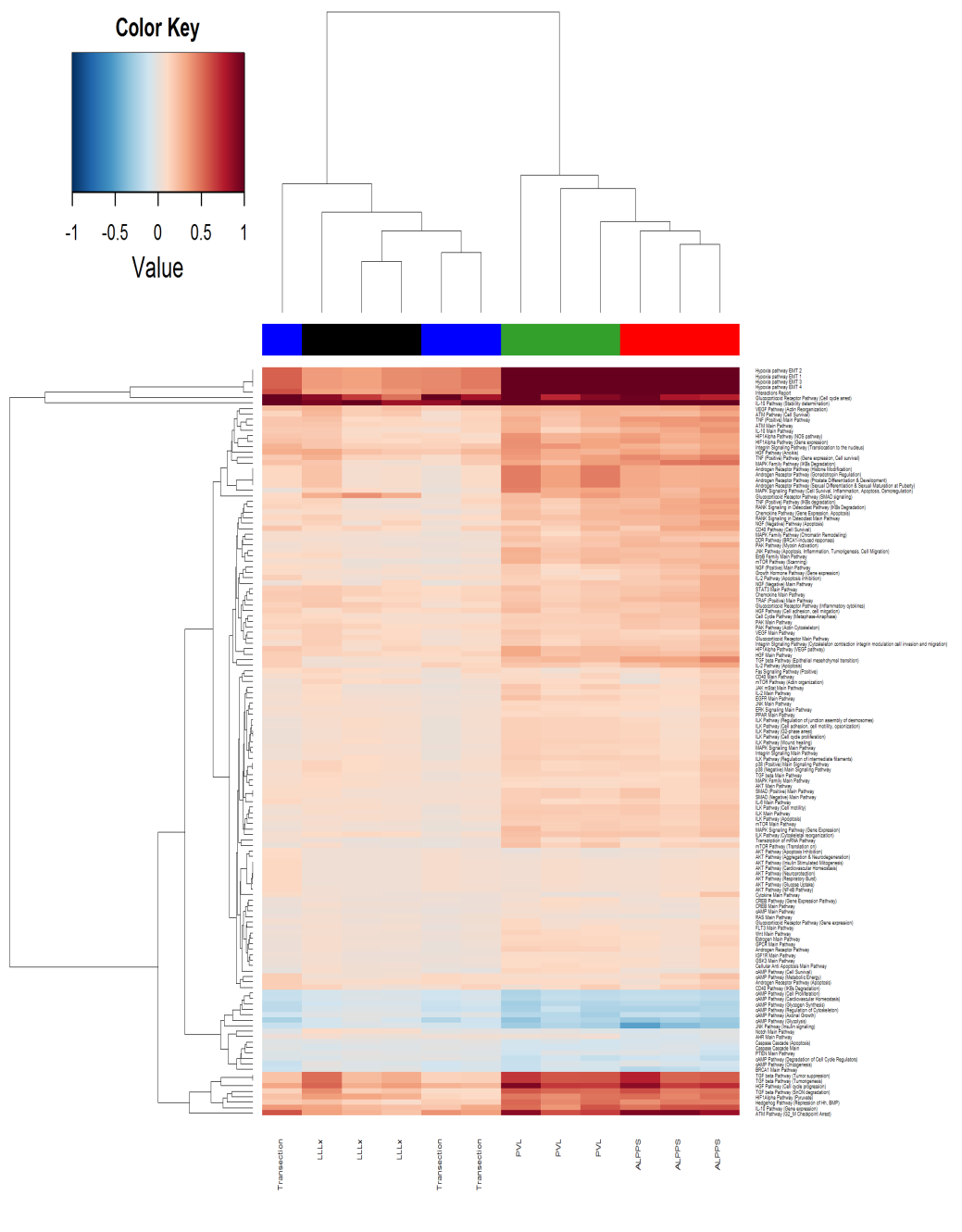

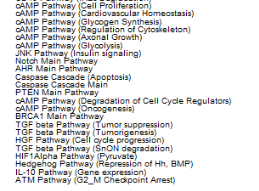

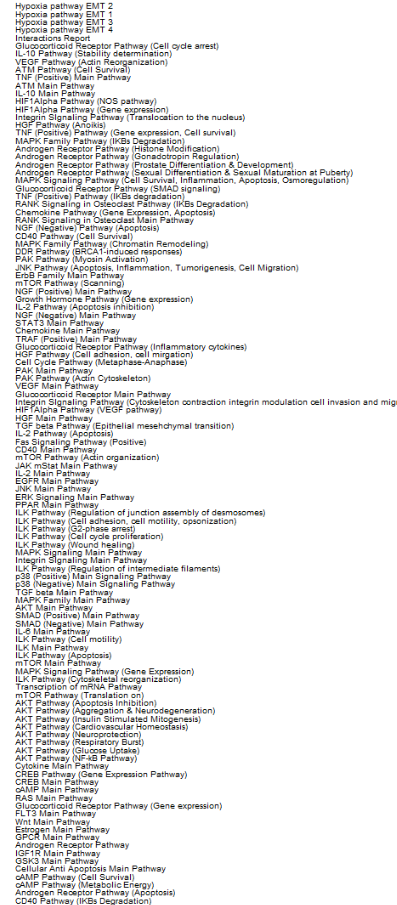

Supplement: Supplementary file 1 [file Data_Sheet_1.DOCX]
