# Supplementary material for: Exploration of the Transcriptional Landscape of ALPPS Reveals the Pathways of Accelerated Liver Regeneration
Source: Front Oncol. 2019 Nov 19;9:1206. doi: 10.3389/fonc.2019.01206 (PMC6882302; doi:10.3389/fonc.2019.01206)
Supplement: Supplementary file 2 [file Data_Sheet_2.DOCX]

**Table 1: Common ISPs activated/silenced 4h post OP (70% Hx and ALPPS)**

| **ISP** | **Mean PAS**  **(70% Hx)** | **Mean PAS**  **(ALPPS)** |
| --- | --- | --- |
| Androgen Receptor Pathway | 0.036425354 | 0.061101387 |
| Androgen Receptor Pathway (Gonadotropin Regulation) | 0.080962033 | 0.170591759 |
| Androgen Receptor Pathway (Histone Modification) | 0.080962033 | 0.170591759 |
| Androgen Receptor Pathway (Prostate Differentiation & Development) | 0.080962033 | 0.170591759 |
| Androgen Receptor Pathway (Sexual Differentiation & Sexual Maturation at Puberty) | 0.080962033 | 0.170591759 |
| ATM Main Pathway | 0.069585724 | 0.164331506 |
| ATM Pathway (G2_M Checkpoint Arrest) | 0.192334187 | 0.758265977 |
| BRCA1 Main Pathway | -0.01618013 | -0.173732307 |
| Caspase Cascade Main | -0.01303648 | -0.051055212 |
| Cellular Anti Apoptosis Main Pathway | 0.027718114 | 0.071826152 |
| EGFR Main Pathway | 0.062796782 | 0.101805817 |
| ErbB Family Main Pathway | 0.05751811 | 0.172579526 |
| ERK Signaling Main Pathway | 0.039288924 | 0.080098885 |
| Estrogen Main Pathway | 0.023150742 | 0.038724647 |
| Glucocorticoid Receptor Main Pathway | 0.018079786 | 0.044023946 |
| Glucocorticoid Receptor Pathway (Inflammatory cytokines) | 0.017244728 | 0.080704052 |
| GPCR Main Pathway | 0.024114074 | 0.049562643 |
| GPCR Pathway (Gene expression) | 0.068563364 | 0.123092979 |
| GSK3 Main Pathway | 0.009850525 | 0.025361913 |
| HGF Main Pathway | 0.062203501 | 0.112692194 |
| HGF Pathway (Cell cycle progression) | 0.337396472 | 0.546430817 |
| Hypoxia pathway EMT 1 | 0.084001749 | 0.691549538 |
| Hypoxia pathway EMT 2 | 0.084001749 | 0.691549538 |
| Hypoxia pathway EMT 3 | 0.084001749 | 0.691549538 |
| Hypoxia pathway EMT 4 | 0.084001749 | 0.691549538 |
| IGF1R Signaling Pathway (Cell survival) | 0.029611615 | 0.096779629 |
| ILK Main Pathway | 0.074326641 | 0.17275502 |
| ILK Pathway (Apoptosis) | 0.091947966 | 0.169660241 |
| ILK Pathway (Cell adhesion, cell motility, opsonization) | 0.095965334 | 0.218655438 |
| ILK Pathway (Cell cycle proliferation) | 0.08622972 | 0.180086957 |
| ILK Pathway (Cell migration, retraction) | 0.094442075 | 0.206157845 |
| ILK Pathway (Cell motility) | 0.080839955 | 0.19368415 |
| ILK Pathway (Cytoskeletal reorganization) | 0.115000716 | 0.249836663 |
| ILK Pathway (G2-phase arrest) | 0.08622972 | 0.180086957 |
| ILK Pathway (Induced cell proliferation) | 0.197724934 | 0.186502548 |
| ILK Pathway (Regulation of intermediate filaments) | 0.106090655 | 0.23979623 |
| ILK Pathway (Regulation of junction assembly of desmosomes) | 0.095197611 | 0.216906194 |
| ILK Pathway (Wound healing) | 0.095197611 | 0.224489964 |
| IL-10 Pathway (Stability determination) | 0.053478054 | 1.848219244 |
| IL-2 Main Pathway | 0.017026437 | 0.113993181 |
| IL-6 Main Pathway | 0.032827423 | 0.042166032 |
| Integrin SIgnaling Main Pathway | 0.067439974 | 0.145964253 |
| JNK Main Pathway | 0.022231589 | 0.043158471 |

**Table 1 (continued): Common ISPs activated/silenced 4h post OP (70% Hx and ALPPS)**

| **ISP** | **Mean PAS**  **(70% Hx)** | **Mean PAS**  **(ALPPS)** |
| --- | --- | --- |
| JNK Pathway (Apoptosis, Inflammation, Tumorigenesis, Cell Migration) | 0.079482132 | 0.207123801 |
| JNK Pathway (Insulin signaling) | -0.07241052 | -0.426061537 |
| MAPK Signaling Main Pathway | 0.0519986 | 0.108187021 |
| MAPK Signaling Pathway (Cell Survival, Inflammation, Apoptosis, Osmoregulation) | 0.212081896 | 0.229612073 |
| MAPK Signaling Pathway (Gene Expression) | 0.091194477 | 0.149905425 |
| Mitochondrial Apopotosis Main Pathway | -0.02214683 | -0.070989145 |
| mTOR Main Pathway | 0.030621819 | 0.063551156 |
| mTOR Pathway (Actin organization) | 0.059587334 | 0.128667337 |
| NGF (Positive) Main Pathway | 0.065652871 | 0.023880418 |
| p38 (Negative) Main Signaling Pathway | 0.03734519 | 0.063658989 |
| p38 (Positive) Main Signaling Pathway | 0.037269285 | 0.06555432 |
| p53 Signaling (Negative) Main Pathway | 0.084470457 | 0.14626007 |
| PAK Main Pathway | 0.034655373 | 0.117954615 |
| PAK Pathway (Actin Cytoskeleton) | 0.027547608 | 0.084506603 |
| PPAR Main Pathway | 0.045990394 | 0.069292444 |
| RANK Signaling in Osteoclast Main Pathway | 0.056148898 | 0.092082069 |
| SMAD (Negative) Main Pathway | 0.139663741 | 0.315152085 |
| SMAD (Positive) Main Pathway | 0.139663741 | 0.315152085 |
| TGF beta Main Pathway | 0.020283971 | 0.053085017 |
| TGF beta Pathway (SnON degradation) | 0.166857414 | 0.576950547 |
| TGF beta Pathway (Tumorigenesis) | 0.238367734 | 0.512365322 |
| TGF beta Pathway (Tumor suppression) | 0.238367734 | 0.512365322 |
| TNF (Positive) Main Pathway | 0.096317033 | 0.224260215 |
| TNF (Positive) Pathway (Gene expression, Cell survival) | 0.157317821 | 0.326862624 |
| TRAF (Positive) Main Pathway | 0.020857177 | 0.044484633 |
| VEGF Main Pathway | 0.058352822 | 0.066069173 |
| VEGF Pathway (Actin Reorganization) | 0.114882119 | 0.070794654 |

**Table 2: Common ISPs activated/silenced 8h post OP (70% Hx and ALPPS)**

| **ISP** | **Mean PAS**  **(70% Hx)** | **Mean PAS**  **(ALPPS)** |
| --- | --- | --- |
| AKT Main Pathway | 0.001489939 | 0.039324986 |
| AKT Pathway (Caspase Cascade) | -0.016328663 | -0.09207135 |
| AKT Pathway (Death Genes) | -0.037076829 | -0.086956275 |
| AKT Pathway (Elevation of Glucose Import) | -0.035125417 | -0.082379629 |
| AKT Pathway (Genetic Stability) | -0.071531433 | -0.096628981 |
| AKT Pathway (JNK Pathway) | -0.037076829 | -0.04938373 |
| AKT Pathway (p73 Mediated Apoptosis) | -0.038136167 | -0.099632161 |
| AKT Pathway (Regeneration of Cyclic Nucleotide) | -0.083476071 | -0.080267331 |
| AKT Pathway (Synaptic Signaling) | -0.007692091 | -0.086401947 |
| Androgen Receptor Pathway | 0.032317147 | 0.06581105 |
| Androgen Receptor Pathway (Degradation) | -0.005475041 | 0.104760752 |
| Androgen Receptor Pathway (Gonadotropin Regulation) | 0.033581217 | 0.215580801 |
| Androgen Receptor Pathway (Histone Modification) | 0.033581217 | 0.215580801 |
| Androgen Receptor Pathway (Prostate Differentiation & Development) | 0.033581217 | 0.215580801 |
| Androgen Receptor Pathway (Sexual Differentiation & Sexual Maturation at Puberty) | 0.033581217 | 0.215580801 |
| ATM Pathway (G2_M Checkpoint Arrest) | 0.774102422 | 0.673356877 |
| BRCA1 Main Pathway | -0.225148624 | -0.237496811 |
| cAMP Pathway (Axonal Growth) | -0.072238113 | -0.147383708 |
| cAMP Pathway (Cardiovascular Homeostasis) | -0.154795957 | -0.137746515 |
| cAMP Pathway (Cell Proliferation) | -0.166703339 | -0.148342401 |
| cAMP Pathway (Degradation of Cell Cycle Regulators) | -0.069907852 | -0.086783896 |
| cAMP Pathway (Endothelial Cell Regulation) | 0.232881728 | 0.308342421 |
| cAMP Pathway (Glycogen Synthesis) | -0.197013037 | -0.175313747 |
| cAMP Pathway (Glycolysis) | -0.131733957 | -0.240993018 |
| cAMP Pathway (Metabolic Energy) | -0.053913131 | -0.171086416 |
| cAMP Pathway (Myocardial Contraction) | -0.047111095 | -0.067357834 |
| cAMP Pathway (Oncogenesis) | -0.154795957 | -0.137746515 |
| cAMP Pathway (Regulation of Cytoskeleton) | -0.135446463 | -0.147502398 |
| Caspase Cascade Main | -0.022075731 | -0.044227804 |
| Caspase Cascade (Activated Tissue Transglutaminase) | -0.019057083 | -0.050644518 |
| Caspase Cascade (Cell Survival) | -0.094997658 | -0.324999998 |
| CD40 Main Pathway | -0.083395013 | -0.084860427 |
| CD40 Pathway (IKBs Degradation) | -0.075428189 | -0.080347816 |
| Cellular Anti Apoptosis Main Pathway | 0.021150735 | 0.076073781 |
| Chemokine Main Pathway | 0.017310316 | 0.02326567 |
| DNA Repair Mechanisms Pathway | -0.058983869 | -0.029466055 |
| EGFR Main Pathway | 0.000583119 | 0.052654656 |
| ErbB Family Main Pathway | -0.022836959 | 0.113731943 |
| ERK Signaling Main Pathway | 0.016777735 | 0.061598696 |
| Erythropoeitin Main Pathway | -0.021783141 | 0.043786725 |
| Estrogen Main Pathway | 0.036987737 | 0.067943991 |
| Glucocorticoid Receptor Main Pathway | 0.046292469 | 0.073127009 |
| Glucocorticoid Receptor Pathway (Cell cycle arrest) | 1.397129082 | 0.764893765 |
| Glucocorticoid Receptor Pathway (Inflammatory cytokines) | 0.018070476 | 0.100259668 |
| GPCR Main Pathway | 0.025010979 | 0.04649428 |
| GPCR Pathway (Gene expression) | 0.055500017 | 0.049541767 |
| GSK3 Main Pathway | 0.017409741 | 0.055630392 |
| Hedgehog Main Pathway | -0.197566955 | -0.078234567 |
| Hedgehog Pathway (Repression of Hh, BMP) | 0.284156044 | 0.239693895 |
| HGF Main Pathway | 0.024263138 | 0.061937683 |
| HGF Pathway (Cell adhesion, cell mirgation) | 0.043270801 | 0.114444101 |
| HGF Pathway (Cell cycle progression) | 0.433490548 | 0.711161357 |
| HGF Pathway (Cell scattering) | -0.130897694 | -0.257142041 |
| HGF Pathway (Cell survival) | -0.130897694 | -0.257142041 |
| HGF Pathway (IP3 pathway) | -0.150532349 | -0.288934509 |
| Hypoxia pathway EMT 1 | 0.800757255 | 0.994945733 |
| Hypoxia pathway EMT 2 | 0.800757255 | 0.994945733 |
| Hypoxia pathway EMT 3 | 0.800757255 | 0.994945733 |
| Hypoxia pathway EMT 4 | 0.800757255 | 0.994945733 |
| ILK Main Pathway | 0.069174915 | 0.158480796 |
| ILK Pathway (Apoptosis) | 0.042836587 | 0.13308184 |
| ILK Pathway (Cell adhesion, cell motility, opsonization) | 0.02815973 | 0.158405777 |
| ILK Pathway (Cell cycle proliferation) | 0.019442823 | 0.128964876 |
| ILK Pathway (Cell migration, retraction) | 0.021983963 | 0.147604194 |
| ILK Pathway (Cell motility) | 0.062411098 | 0.166428052 |
| ILK Pathway (Cytoskeletal reorganization) | 0.081346983 | 0.209266644 |
| ILK Pathway (G2-phase arrest) | 0.019442823 | 0.128964876 |
| ILK Pathway (Induced cell proliferation) | 0.060269873 | 0.240071141 |
| ILK Pathway (Regulation of intermediate filaments) | 0.040950447 | 0.17489141 |
| ILK Pathway (Regulation of junction assembly of desmosomes) | 0.027934452 | 0.153280433 |
| ILK Pathway (Wound healing) | 0.034165008 | 0.163083322 |
| IL-10 Main Pathway | -0.012682123 | 0.105900526 |
| IL-10 Pathway (Gene expression) | 0.0472062 | 0.144551561 |
| IL-10 Pathway (Stability determination) | 1.728536757 | 2.427285447 |
| IL-10 Pathway (Translational modulation) | 0.057898184 | 0.256395394 |
| IL-2 Main Pathway | 0.039264953 | 0.091129878 |
| IL-2 Pathway (Actin reorganization) | -0.275828184 | -0.035288567 |
| IL-2 Pathway (Apoptosis) | 0.029521257 | 0.054911545 |
| IL-2 Pathway (Apoptosis inhibition) | -0.002934755 | 0.032438133 |
| IL-6 Main Pathway | 0.044750517 | 0.064122058 |
| Integrin SIgnaling Main Pathway | 0.03047794 | 0.112990206 |
| Integrin SIgnaling Pathway (Cell survival) | -0.086211226 | -0.084867535 |
| Integrin SIgnaling Pathway (Translocation to the nucleus) | 0.401943517 | 0.164758239 |
| JAK mStat Pathway (Akt pathway) | -0.070087662 | -0.084453053 |
| JNK Main Pathway | 0.00809321 | 0.053791386 |
| JNK Pathway (Apoptosis, Inflammation, Tumorigenesis, Cell Migration) | 0.083876965 | 0.173598474 |
| MAPK Family Main Pathway | 0.00622822 | 0.024634703 |
| MAPK Family Pathway (Gene Expression) | 0.008328632 | 0.053714469 |
| MAPK Family Pathway (IKBs Degradation) | 0.098636863 | 0.124388587 |
| MAPK Signaling Main Pathway | 0.035825034 | 0.092707776 |
| MAPK Signaling Pathway (Cell Survival, Inflammation, Apoptosis, Osmoregulation) | 0.19440265 | 0.32660244 |
| MAPK Signaling Pathway (Gene Expression) | 0.066900445 | 0.115102486 |
| Mitochondrial Apopotosis Pathway (DNA fragmentation) | -0.253327087 | -0.285077442 |
| mTOR Main Pathway | 0.032547413 | 0.099140596 |
| mTOR Pathway (Actin organization) | -0.000472793 | 0.047664908 |
| NGF (Negative) Pathway (Apoptosis) | 0.055802205 | 0.122714664 |
| NGF (Positive) Main Pathway | -0.033013103 | 0.026249716 |
| p38 (Negative) Main Signaling Pathway | 0.02007549 | 0.053671637 |
| p38 (Positive) Main Signaling Pathway | 0.020034686 | 0.054987191 |
| p53 Signaling (Negative) Main Pathway | 0.082978824 | 0.173657007 |
| PAK Main Pathway | 0.046268481 | 0.114561118 |
| PAK Pathway (Actin Cytoskeleton) | 0.012854696 | 0.068031395 |
| PAK Pathway (Myosin Activation) | 0.050190028 | 0.158422694 |
| PPAR Main Pathway | 0.050300794 | 0.075474807 |
| PTEN Main Pathway | 0.05757828 | 0.049804857 |
| RANK Signaling in Osteoclast Main Pathway | 0.079480333 | 0.106001592 |
| RANK Signaling in Osteoclast Pathway (IKBs Degradation) | 0.064113961 | 0.080852581 |
| RAS Main Pathway | -0.014860591 | -0.038841977 |
| SMAD (Negative) Main Pathway | 0.175370166 | 0.240575867 |
| SMAD (Positive) Main Pathway | 0.175370166 | 0.240575867 |
| Cell Cycle Pathway (Metaphase-Anaphase) | 0.147895336 | 0.223879531 |
| STAT3 Main Pathway | 0.03313539 | 0.073137421 |
| TGF beta Main Pathway | 0.0061111 | 0.073003159 |
| TGF beta Pathway (SnON degradation) | 0.266307663 | 0.564491509 |
| TGF beta Pathway (Tumorigenesis) | 0.380439519 | 0.644093404 |
| TGF beta Pathway (Tumor suppression) | 0.380439519 | 0.644093404 |
| TNF (Negative) Main Pathway | 0.006207219 | 0.071396548 |
| TNF (Negative) Pathway (Apoptosis) | -0.050757976 | 0.079796142 |
| TNF (Positive) Main Pathway | 0.190544598 | 0.148989258 |
| TNF (Positive) Pathway (Gene expression, Cell survival) | 0.218297874 | 0.134870363 |
| TRAF (Negative) Pathway (IKBs Degradation) | 0.125727888 | 0.07170541 |
| TRAF (Positive) Main Pathway | 0.027504352 | 0.070103647 |
| TRAF (Positive) Pathway (IKBs Degradation) | 0.125727888 | 0.07170541 |
| Ubiquitin Proteasome Main Pathway | -0.023246044 | -0.065262947 |
| VEGF Main Pathway | 0.010450379 | 0.033278447 |
| VEGF Pathway (Actin Reorganization) | 0.136446006 | 0.122529101 |
| Wnt Main Pathway | 0.036835744 | 0.082310699 |

***Table 2: ISPs with PAS values ≥ 0.1. Pathway with PAS values ≥ 0.5 are highlighted***
