# Supplementary material for: Exploration of the Transcriptional Landscape of ALPPS Reveals the Pathways of Accelerated Liver Regeneration
Source: Front Oncol. 2019 Nov 19;9:1206. doi: 10.3389/fonc.2019.01206 (PMC6882302; doi:10.3389/fonc.2019.01206)
Supplement: Supplementary file 3 [file Data_Sheet_3.DOCX]

| ***Intracellular Signaling Pathway (ISP)*** | ***mean PAS ALPPS (4h)*** | ***mean PAS ALPPS (8h)*** | ***mean PAS ALPPS (12h)*** | ***mean 70% Hx (32h)*** | ***mean 70% Hx (48h)*** |
| --- | --- | --- | --- | --- | --- |
|  |  |  |  |  |  |
| **AKT Main Pathway** |  |  | 0.145864574 | 0.318676876 |  |
| **AKT Pathway (Cell Survival)** |  |  |  | 0.175205676 |  |
| **AKT Pathway (Glycogen Synthesis)** |  |  |  | 0.150176293 |  |
| **AKT Pathway (p53 Degradation)** |  |  |  | 0.131404257 |  |
| **Androgen Receptor Pathway (Degradation)** |  | 0.104760752 |  |  |  |
| **Androgen Receptor Pathway (Apoptosis)** |  |  | 0.102401093 | 0.144154649 | 0.135903491 |
| **Androgen Receptor Pathway (Gonadotropin Regulation)** |  | 0.215580801 | 0.29128311 |  |  |
| **Androgen Receptor Pathway (Histone Modification)** |  | 0.215580801 | 0.29128311 |  |  |
| **Androgen Receptor Pathway (Prostate Differentiation & Development)** |  | 0.215580801 | 0.29128311 |  |  |
| **Androgen Receptor Pathway (Sexual Differentiation & Sexual Maturation at Puberty)** |  | 0.215580801 | 0.29128311 |  |  |
| **ATM Main Pathway** | 0.164331506 |  | 0.349845013 | 0.129388507 |  |
| **ATM Pathway (Apoptosis, Senescense)** |  |  |  | 0.143933545 | -0.128149846 |
| **ATM Pathway (Cell Cicle Checkpoint Control)** |  |  |  | 0.194005778 | 0.1475622 |
| **ATM Pathway (Cell Survival)** |  |  | 0.27635038 | 0.105395963 |  |
| **ATM Pathway (G2_M Checkpoint Arrest)** | 0.758265977 | 0.673356877 | 0.898728706 | -0.651298809 | -1.173779041 |
| **ATM Pathway (G2 Mitosis Progression)** |  |  |  | -1.894466839 | -2.064555183 |
| **ATM Pathway (S-Phase Progression)** |  |  |  | -0.194826223 |  |
| **ATM Pathway (Checkpoint Activation)** |  |  |  | 0.194005778 | 0.1475622 |
| **ATM Pathway (DNA Repair)** |  |  |  | 0.526332533 | 0.764544748 |
| **ATM Pathway (MDMX Ubiquitination, Degradation)** |  |  |  |  | -0.301342015 |
| **ATM Pathway (NF-kB Pathway)** |  |  |  | 0.194005778 | 0.1475622 |
| **ATM Pathway (Synaptic Vesicle Transport)** |  |  |  | 0.194005778 | 0.1475622 |
| **ATM Pathway (S-Phase Arrest)** |  |  |  | 0.996035253 | 0.802718287 |
| **BRCA1 Main Pathway** |  | -0.237496811 | -0.169668604 | 0.292340978 | 0.257715212 |
| **cAMP Pathway (Axonal Growth)** |  | -0.147383708 | -0.167920598 |  | -0.15462705 |
| **cAMP Pathway (Cardiovascular Homeostasis)** |  | -0.137746515 | -0.160986684 | -0.247536264 | -0.239458868 |
| **cAMP Pathway (Cell Growth)** |  |  |  | -0.410706855 | -0.322152531 |
| **cAMP Pathway (Cell Proliferation)** |  | -0.148342401 | -0.173370275 |  | -0.109192998 |
| **cAMP Pathway (Cell Survival, Chemotaxis)** |  |  |  | -0.308030141 | -0.241614398 |
| **cAMP Pathway (Cytokine Production)** |  |  |  |  | -0.13806537 |
| **cAMP Pathway (Degradation of Cell Cycle Regulators)** |  |  | -0.131923642 |  |  |
| **cAMP Pathway (Endothelial Cell Regulation)** |  | 0.308342421 |  | 0.157543638 |  |
| **cAMP Pathway (Glycogen Synthesis)** |  | -0.175313747 | -0.204892143 |  | -0.12904627 |
| **cAMP Pathway (Glycolysis)** |  | -0.240993018 | -0.290030933 |  |  |
| **cAMP Pathway (Metabolic Energy)** |  | -0.171086416 | 0.149192792 |  |  |
| **cAMP Pathway (Oncogenesis)** |  | -0.137746515 |  |  | -0.101393498 |
| **cAMP Pathway (Protein Retention)** |  |  |  | 0.137868849 |  |
| **cAMP Pathway (Regulation of Cytoskeleton)** |  | -0.147502398 | -0.232283523 |  |  |
| **Caspase Cascade (Cell Survival)** | -0.195975105 | -0.324999998 |  |  | -0.128462276 |
| **CD40 Pathway (Cell Survival)** |  |  | 0.268730557 |  |  |
| **CD40 Pathway (IKBs Degradation)** |  |  | 0.140117493 |  |  |
| **Cell Cycle Pathway (Metaphase-Anaphase)** |  | 0.223879531 | 0.214888509 | 0.519644505 | 0.547505918 |
| **Cell Cycle Pathway (Origin of S-phase)** |  |  |  | 1.314349782 | 1.115747946 |
| **Cell Cycle Pathway (SCC during S-phase)** |  |  |  | -0.152618976 | -0.224782007 |
| **Cell Cycle Pathway (End of S-phase)** |  |  |  | 0.225368236 |  |
| **Chemokine Main Pathway** |  |  | 0.238571922 |  |  |
| **Chemokine Pathway (Gene Expression, Apoptosis)** |  |  | 0.312245531 |  |  |
| **Chromatin Main Pathway** |  |  |  | 0.201536717 | 0.182233623 |
| **Chromatin Pathway (Octamer Sliding)** |  |  |  | 0.232542366 | 0.210269565 |
| **Chromatin Pathway (Octamer Transfer)** |  |  |  | 0.201536717 | 0.182233623 |
| **Circadian Main Pathway** |  |  |  |  | 0.133933923 |
| **Cytokine Main Pathway** |  |  | 0.122276137 |  |  |
| **DDR Main pathway** |  |  |  | 0.843294475 | 0.811701965 |
| **DDR pathway Apoptosis** |  |  |  | 1.103896527 | 1.030312638 |
| **DDR Pathway (BRCA1-induced responses)** | -0.207103146 |  | 0.211150507 |  | -0.172474809 |
| **DDR pathway (MMR)** |  |  |  | 0.681375582 | 0.644804225 |
| **DDR Pathway (NER)** |  |  |  | 0.392225911 | 0.243724844 |
| **DNA Repair Mechanisms Pathway** |  |  |  | 0.259081043 | 0.177862826 |
| **EGFR Main Pathway** | 0.101805817 |  | 0.134578206 |  |  |
| **ErbB Family Main Pathway** | 0.172579526 | 0.113731943 | 0.227081637 |  |  |
| **ERK Signaling Main Pathway** |  |  | 0.119446361 | 0.110954497 |  |
| **Estrogen Main Pathway** |  |  | 0.108667621 |  |  |
| **Fas Signaling Pathway (Negative)** |  |  |  | 0.118142333 |  |
| **Fas Signaling Pathway (Positive)** |  |  | 0.121782733 |  |  |
| **Glucocorticoid Receptor Main Pathway** |  |  | 0.171150635 |  |  |
| **Glucocorticoid Receptor Pathway (Cell cycle arrest)** |  | 0.764893765 | 0.838599963 | 0.772394462 | 0.379311121 |
| **Glucocorticoid Receptor Pathway (Inflammatory cytokines)** |  | 0.100259668 | 0.222454482 |  |  |
| **Glucocorticoid Receptor Pathway (SMAD signaling)** |  |  | 0.286817122 |  |  |
| **GPCR Main Pathway** |  |  | 0.112142876 |  |  |
| **GPCR Pathway (Gene expression)** | 0.123092979 |  |  |  |  |
| **Growth Hormone Pathway (Cell survival)** |  |  |  |  | 0.506706498 |
| **Growth Hormone Pathway (Gene expression)** |  |  | 0.196176338 |  |  |
| **Growth Hormone Pathway (Protein synthesis)** |  |  |  |  | 0.760059747 |
| **GSK3 Pathway (Gene expression)** |  |  |  |  | 0.10089841 |
| **HGF Main Pathway** | 0.112692194 |  |  |  |  |
| **HGF Pathway (Cell cycle progression)** | 0.546430817 |  |  |  |  |
| **Hedgehog Main Pathway** |  |  |  | 0.261337874 | 0.310878582 |
| **Hedgehog Pathway (Repression of Hh, BMP)** |  | 0.239693895 | 0.435546032 | 0.500880755 | 0.287788355 |
| **HGF Main Pathway** |  |  | 0.245054096 |  |  |
| **HGF Pathway (Anoikis)** |  |  | 0.318576497 | 0.159890844 | 0.150631405 |
| **HGF Pathway (Cell adhesion, cell mirgation)** |  | 0.114444101 | 0.208782971 |  | 0.106278802 |
| **HGF Pathway (Cell cycle progression)** |  | 0.711161357 | 0.752896787 | 0.188458187 | 0.20763753 |
| **HGF Pathway (Cell scattering)** |  | -0.257142041 |  | 0.134808767 | 0.115226277 |
| **HGF Pathway (Cell survival)** |  | -0.257142041 |  | 0.134808767 | 0.115226277 |
| **HGF Pathway (IP3 pathway)** |  | -0.288934509 |  | 0.102468379 | 0.132510219 |
| **HIF1Alpha Pathway (Gene expression)** |  |  | 0.332634605 |  | 0.107147138 |
| **HIF1Alpha Pathway (NOS pathway)** |  |  | 0.317878382 |  | 0.123631313 |
| **HIF1Alpha Pathway (Pyruvate)** |  |  | 0.486021236 |  | 0.107147138 |
| **HIF1Alpha Pathway (VEGF pathway)** |  |  | 0.204896449 |  | 0.100450442 |
| **Hypoxia pathway EMT 1** |  | 0.994945733 | 1.327324605 | 0.682544691 | 0.889378469 |
| **Hypoxia pathway EMT 2** |  | 0.994945733 | 1.327324605 | 0.682544691 | 0.889378469 |
| **Hypoxia pathway EMT 3** |  | 0.994945733 | 1.327324605 | 0.682544691 | 0.889378469 |
| **Hypoxia pathway EMT 4** |  | 0.994945733 | 1.327324605 | 0.682544691 | 0.889378469 |
| **IGF1R Signaling Pathway (Glucose uptake)** |  |  |  | 0.210246811 |  |
| **IGF1R Signaling Pathway (IKB degradation)** |  |  |  | 0.130200644 |  |
| **ILK Main Pathway** | 0.17275502 | 0.158480796 | 0.177750615 | 0.165981895 | 0.148337143 |
| **ILK Pathway (Apoptosis)** | 0.169660241 | 0.13308184 | 0.169195727 | 0.259877673 | 0.24961264 |
| **ILK Pathway (Cell adhesion, cell motility, opsonization)** | 0.218655438 | 0.158405777 | 0.130072058 | 0.280924068 | 0.272371368 |
| **ILK Pathway (Cell cycle proliferation)** | 0.180086957 | 0.128964876 | 0.12435912 | 0.252424525 | 0.252126257 |
| **ILK Pathway (Cell migration, retraction)** | 0.206157845 | 0.147604194 |  | 0.276464956 | 0.268048013 |
| **ILK Pathway (Cell motility)** | 0.19368415 | 0.166428052 | 0.185612859 | 0.244698871 | 0.220225189 |
| **ILK Pathway (Cytoskeletal reorganization)** | 0.249836663 | 0.209266644 | 0.196497064 | 0.272833911 | 0.256990754 |
| **ILK Pathway (G2-phase arrest)** | 0.180086957 | 0.128964876 | 0.12435912 | 0.252424525 | 0.252126257 |
| **ILK Pathway (Induced cell proliferation)** | 0.186502548 | 0.240071141 |  | 0.130572193 | 0.1447218 |
| **ILK Pathway (Regulation of intermediate filaments)** | 0.23979623 | 0.17489141 | 0.149199644 | 0.278676675 | 0.276721151 |
| **ILK Pathway (Regulation of junction assembly of desmosomes)** | 0.216906194 | 0.153280433 | 0.123949271 | 0.278676675 | 0.270192398 |
| **ILK Pathway (Wound healing)** | 0.224489964 | 0.163083322 | 0.136041827 | 0.290956887 | 0.282572062 |
| **IL-10 Pathway (Stability determination)** | 1.848219244 | 0.105900526 | 0.32063802 | 0.114906409 | 0.130160706 |
| **IL-10 Pathway (Gene expression)** |  | 0.144551561 | 0.56045593 |  |  |
| **IL-10 Pathway (Stability determination)** |  | 2.427285447 | 2.166319182 |  |  |
| **IL-10 Pathway (Translational modulation)** |  | 0.256395394 |  |  |  |
| **IL-2 Main Pathway** | 0.113993181 |  | 0.121934659 |  |  |
| **IL-2 Pathway (Apoptosis)** |  |  | 0.276000022 |  | 0.15439179 |
| **IL-2 Pathway (Apoptosis inhibition)** |  |  | 0.234077099 |  | 0.168415384 |
| **IL-2 Pathway (Actin reorganization)** |  |  |  | 0.12733996 | 0.12633063 |
| **IL-6 Main Pathway** |  |  | 0.151970913 |  |  |
| **Integrin SIgnaling Main Pathway** | 0.145964253 | 0.112990206 | 0.127763162 | 0.164202433 | 0.159401091 |
| **Integrin SIgnaling Pathway (Cytoskeleton contraction integrin modulation cell invasion and migration)** |  |  | 0.193273261 |  |  |
| **Integrin SIgnaling Pathway (Translocation to the nucleus)** |  | 0.164758239 | 0.279869502 | 0.119527902 | 0.126621032 |
| **JAK mStat Main Pathway** |  |  | 0.127115987 | 0.140946372 | 0.113820344 |
| **JNK Main Pathway** |  |  | 0.12885844 |  |  |
| **JNK Pathway (Apoptosis, Inflammation, Tumorigenesis, Cell Migration)** | 0.207123801 | 0.173598474 | 0.221695559 | 0.106506495 |  |
| **JNK Pathway (Insulin signaling)** |  |  | -0.390675465 |  |  |
| **MAPK Family Main Pathway** |  |  | 0.14234341 | 0.121667205 | 0.109014177 |
| **MAPK Family Pathway (Chromatin Remodeling)** |  |  | 0.228857478 |  |  |
| **MAPK Signaling Pathway (Cell Survival, Inflammation, Apoptosis, Osmoregulation)** |  | 0.32660244 | 0.281599767 |  | 0.147806725 |
| **MAPK Family Pathway (IKBs Degradation)** |  | 0.124388587 | 0.451402892 |  |  |
| **MAPK Family Pathway (Cytoskeleton)** | 0.14887561 |  |  |  | -0.205917383 |
| **MAPK Signaling Pathway (Gene Expression)** | 0.149905425 | 0.115102486 | 0.179606895 |  |  |
| **Mitochondrial Apopotosis Pathway (DNA fragmentation)** | -0.269274386 | -0.285077442 |  | -0.185180947 | -0.169863102 |
| **Mitochondrial Apopotosis Pathway (Gene expression)** |  |  |  | -0.315825561 | -0.301342015 |
| **Mismatch Repair Main Pathway** |  |  |  | 1.071119808 | 0.769970494 |
| **mTOR Main Pathway** |  |  | 0.160998471 |  |  |
| **mTOR Pathway (Actin organization)** | 0.128667337 |  |  |  |  |
| **mTOR Pathway (Scanning)** |  |  | 0.240188141 |  |  |
| **mTOR Pathway (Translation on)** |  |  | 0.13744706 |  |  |
| **NHEJ mechanisms of DSBs repair effect** |  |  |  | 0.328670395 | 0.30369151 |
| **NGF (Negative) Main Pathway** |  |  | 0.21879972 |  |  |
| **NGF (Negative) Pathway (Apoptosis)** |  | 0.122714664 | 0.290820287 |  |  |
| **NGF (Positive) Main Pathway** |  |  | 0.227754796 |  |  |
| **p38 (Negative) Main Signaling Pathway** |  |  | 0.160845843 |  |  |
| **p38 (Positive) Main Signaling Pathway** |  |  | 0.161839745 |  |  |
| **p53 Signaling (Negative) Main Pathway** | 0.14626007 | 0.173657007 |  |  |  |
| **PAK Main Pathway** | 0.117954615 | 0.114561118 | 0.213752316 |  |  |
| **PAK Pathway (Actin Cytoskeleton)** |  |  | 0.214543455 | 0.103543769 |  |
| **PAK Pathway (Myosin Activation)** | 0.176492452 | 0.158422694 | 0.271887147 |  |  |
| **RANK Signaling in Osteoclast Main Pathway** |  | 0.106001592 | 0.273309261 |  |  |
| **RANK Signaling in Osteoclast Pathway (IKBs Degradation)** |  |  | 0.29341188 |  |  |
| **RAS Main Pathway** |  |  |  |  | 0.108911208 |
| **SMAD (Negative) Main Pathway** | 0.315152085 | 0.240575867 | 0.162290767 |  |  |
| **SMAD (Positive) Main Pathway** | 0.315152085 | 0.240575867 | 0.162290767 |  |  |
| **STAT3 Main Pathway** |  |  | 0.236298372 |  |  |
| **TGF beta Main Pathway** |  |  | 0.141713288 |  |  |
| **TGF beta Pathway (Epithelial mesehchymal transition)** |  |  | 0.366992731 |  |  |
| **TGF beta Pathway (SnON degradation)** |  | 0.564491509 | 0.515297501 |  |  |
| **TGF beta Pathway (Tumorigenesis)** |  | 0.644093404 | 0.614227285 |  |  |
| **TGF beta Pathway (Tumor suppression)** |  | 0.644093404 | 0.614227285 |  |  |
| **TNF (Negative) Main Pathway** |  |  |  | 0.105249839 |  |
| **TNF (Negative) Pathway (Apoptosis)** |  |  |  | 0.117632173 |  |
| **TNF (Positive) Main Pathway** | 0.224260215 | 0.148989258 | 0.381605393 |  |  |
| **TNF (Positive) Pathway (Gene expression, Cell survival)** |  | 0.134870363 | 0.414691815 |  | 0.107554454 |
| **TNF (Positive) Pathway (IKBs degradation)** |  |  | 0.300376339 |  |  |
| **TRAF (Positive) Main Pathway** |  |  | 0.266135534 |  |  |
| **Ubiquitin Proteasome Main Pathway** |  |  |  |  | 0.115793209 |
| **Ubiquitin Proteasome Pathway (Degraded Protein)** |  |  |  | 0.112024452 | 0.191727433 |
| **VEGF Main Pathway** |  |  | 0.14673194 |  |  |
| **VEGF Pathway (Actin Reorganization)** |  | 0.122529101 | 0.323931678 | 0.186678743 | 0.130696122 |
| **Wnt Main Pathway** |  |  | 0.101839692 |  |  |
