# Supplementary figures and images for: Exploration of the Transcriptional Landscape of ALPPS Reveals the Pathways of Accelerated Liver Regeneration
Source: Front Oncol. 2019 Nov 19;9:1206. doi: 10.3389/fonc.2019.01206 (PMC6882302; doi:10.3389/fonc.2019.01206)

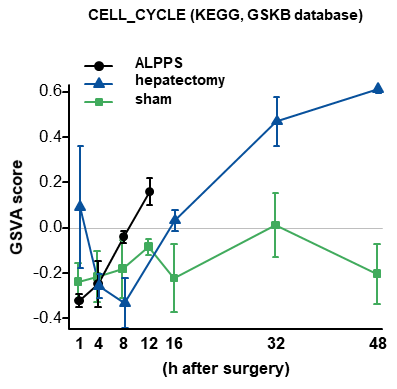

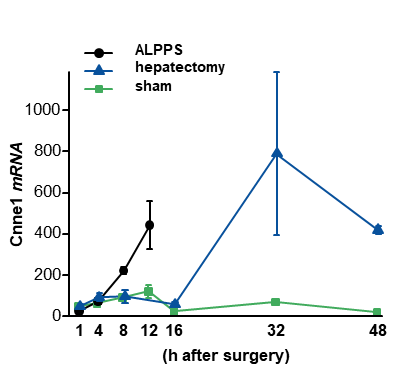

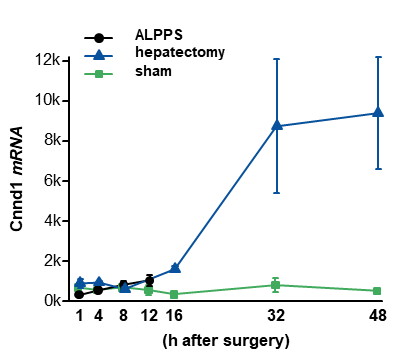


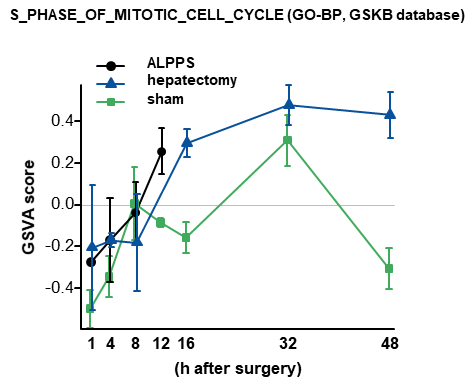


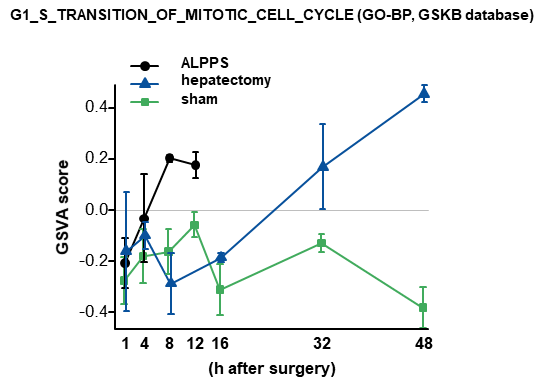

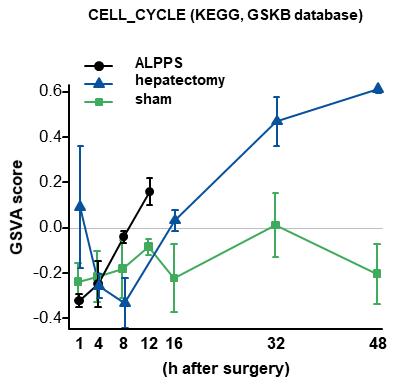


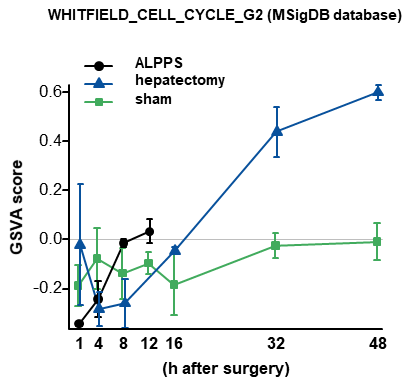

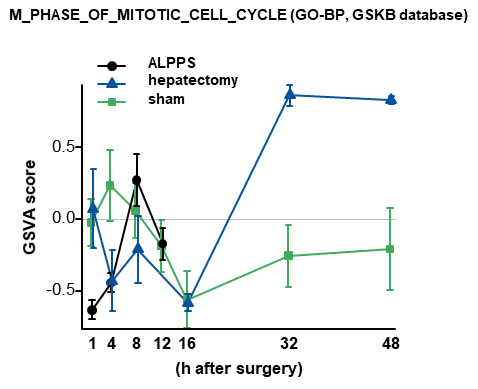

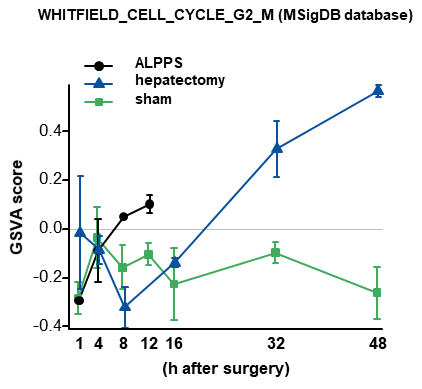


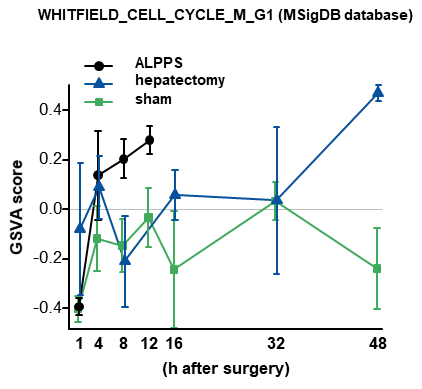

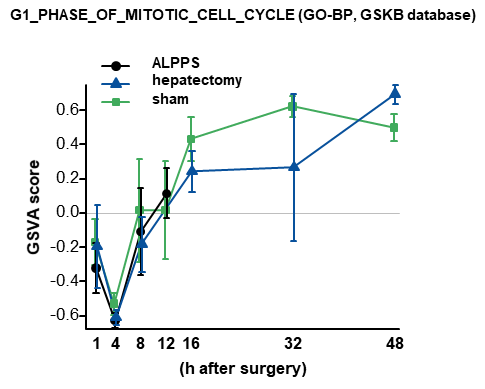

Supplement: Supplementary file 4 [file Data_Sheet_4.DOCX]
