# Supplementary material for: Exploration of the Transcriptional Landscape of ALPPS Reveals the Pathways of Accelerated Liver Regeneration
Source: Front Oncol. 2019 Nov 19;9:1206. doi: 10.3389/fonc.2019.01206 (PMC6882302; doi:10.3389/fonc.2019.01206)
Supplement: Supplementary file 5 [file Data_Sheet_5.DOCX]

**1) ISPs unique for ALPPS, PVL and transection (T=4h post surgery)**

**2) 6/8 common ISPs significantly changed, ALPPS vs transection (t=4h post surgery)**

**3) 14/33 common ISPs significantly changed, ALPPS vs PVL (t=4h post surgery)**

**4) 26/27 common ISPs significantly changed, ALPPS vs PVL and ALPPS vs transection (t=4h post surgery)**
