# Supplementary material for: Exploration of the Transcriptional Landscape of ALPPS Reveals the Pathways of Accelerated Liver Regeneration
Source: Front Oncol. 2019 Nov 19;9:1206. doi: 10.3389/fonc.2019.01206 (PMC6882302; doi:10.3389/fonc.2019.01206)

**LLX over time (24 affected ISPs):**


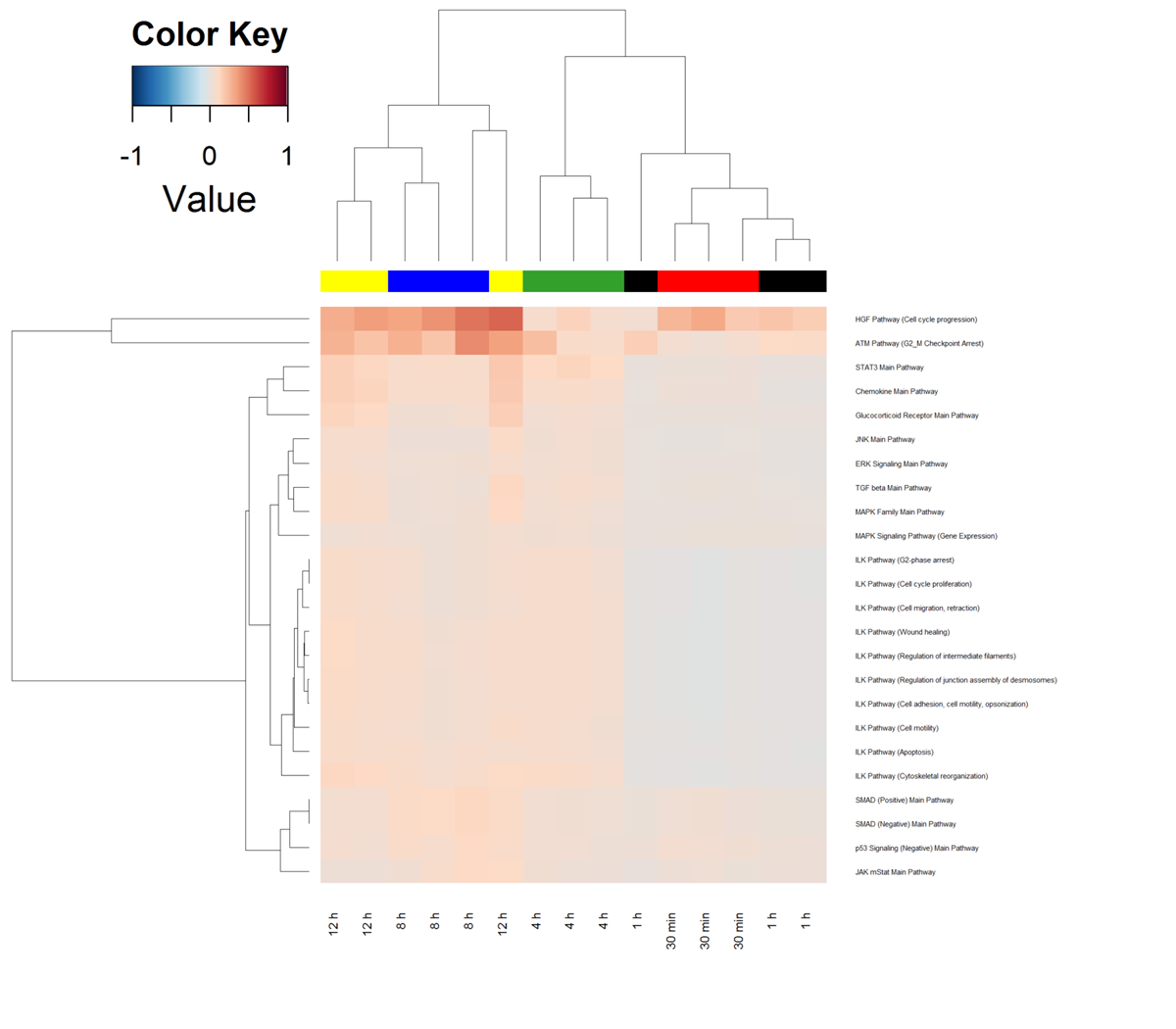


**Transection over time (46 affected ISPs):**


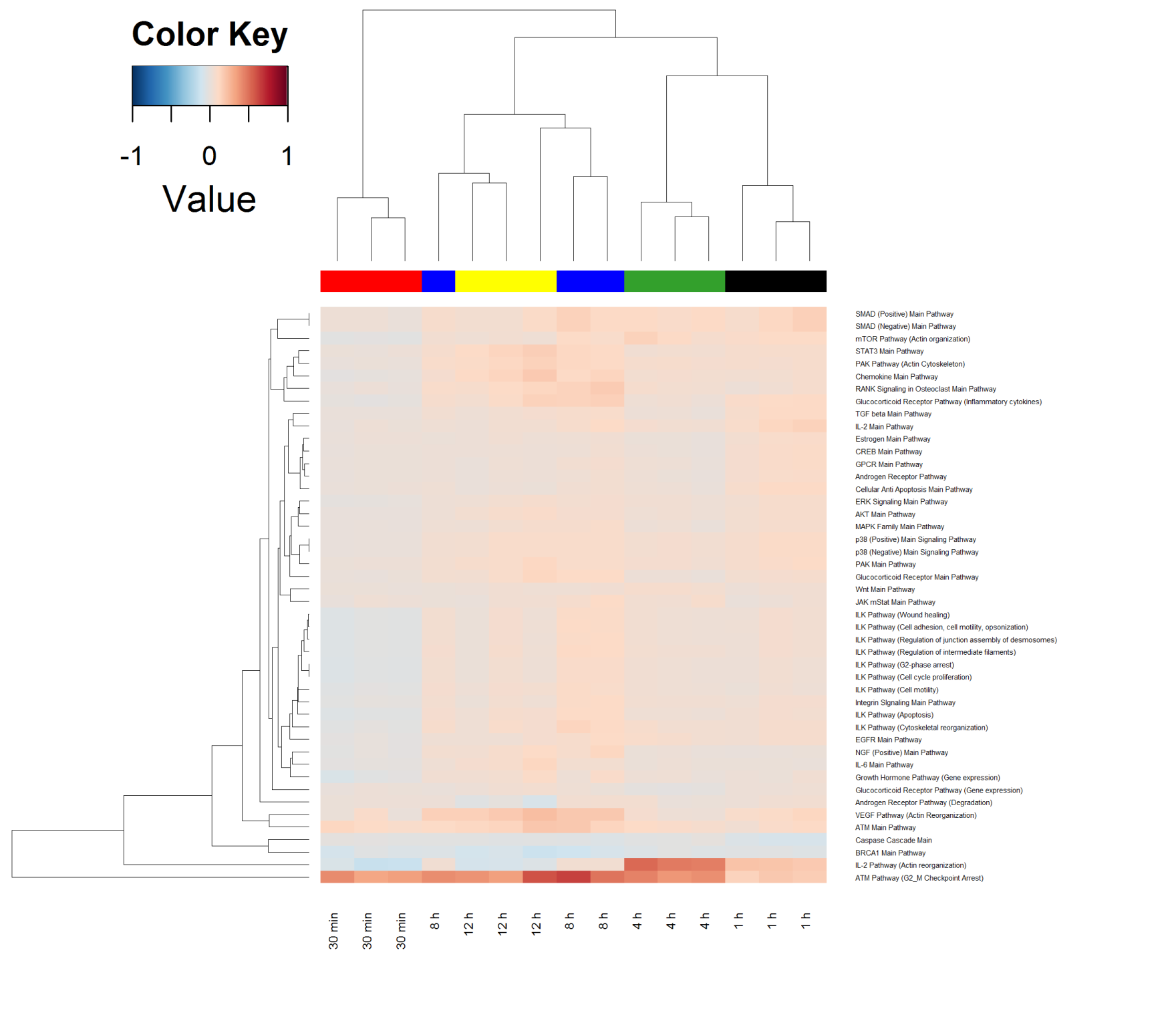


**PVL over time (88 affected ISPs):**

**
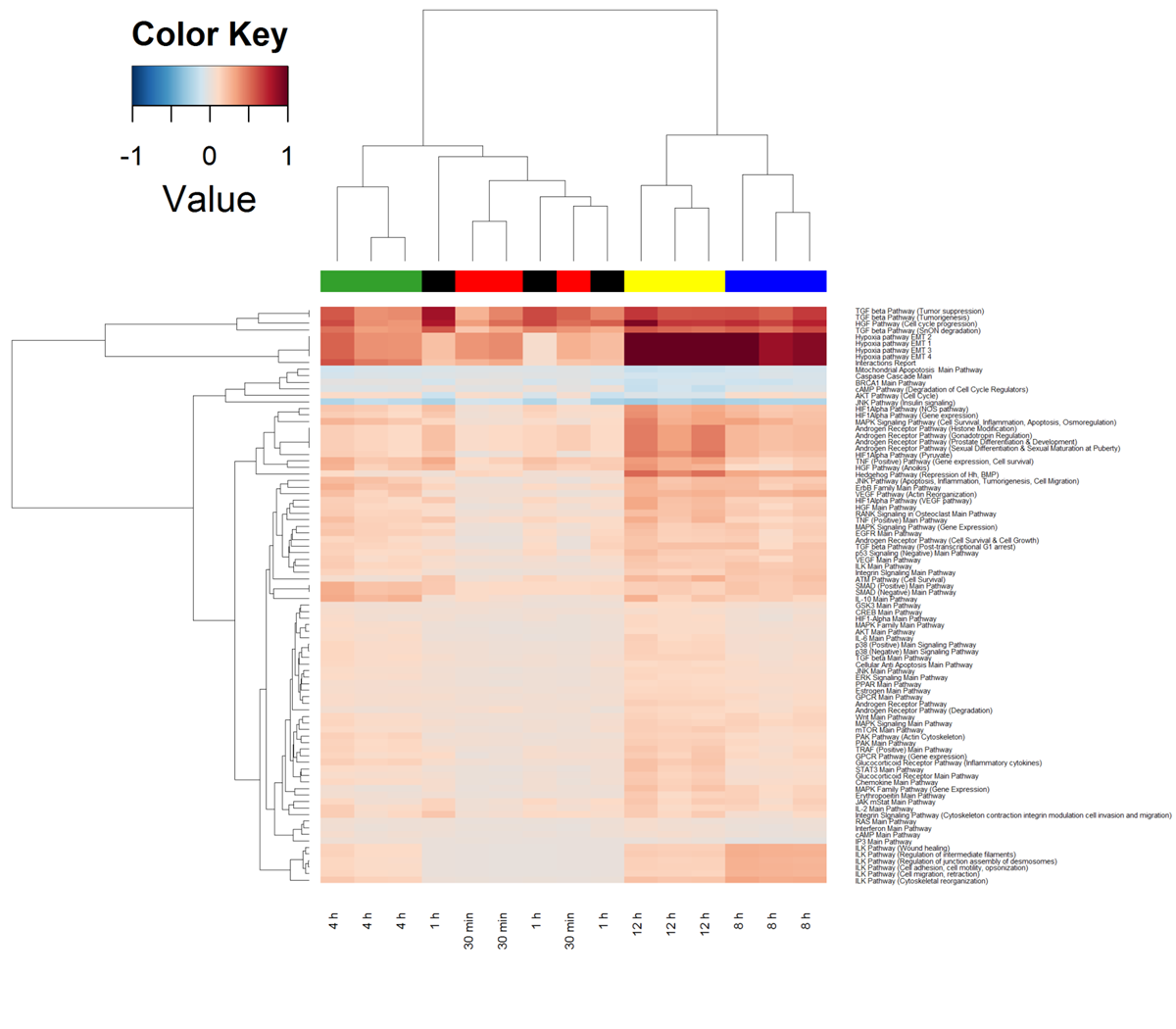
**

**ALPPS over time (72 affected ISPs)**


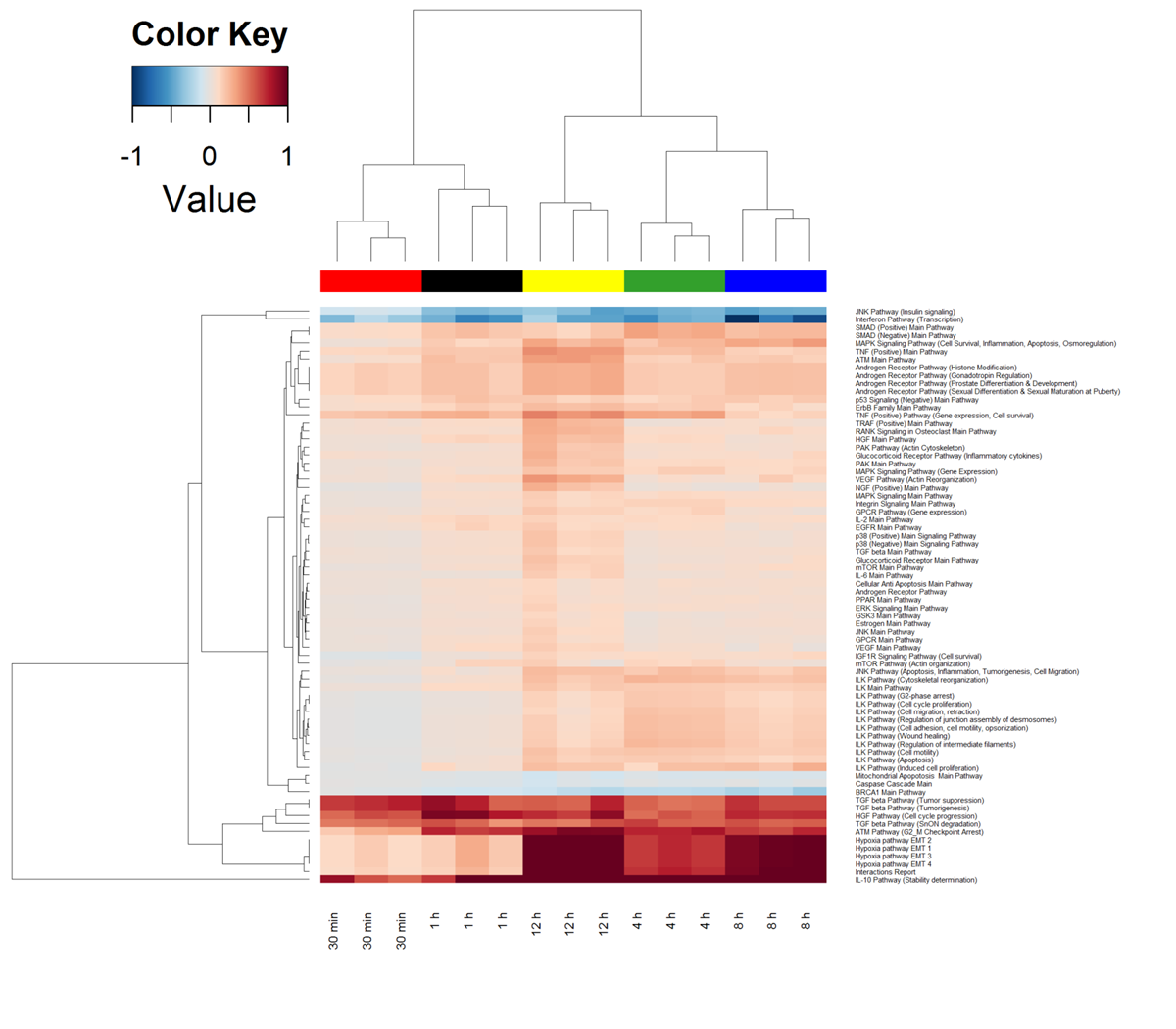

Supplement: Supplementary file 6 [file Data_Sheet_6.DOCX]
