# Supplementary material for: Exploration of the Transcriptional Landscape of ALPPS Reveals the Pathways of Accelerated Liver Regeneration
Source: Front Oncol. 2019 Nov 19;9:1206. doi: 10.3389/fonc.2019.01206 (PMC6882302; doi:10.3389/fonc.2019.01206)
Supplement: Supplementary file 7 [file Data_Sheet_7.DOCX]

***T=1h post surgery:***

***T=4h post surgery:***

***T=4h post surgery (continued):***

***T=8h post surgery:***

***T=8h post surgery (continued):***

***T=8h post surgery (continued):***

***T=12h post surgery:***

***T=12h post surgery (continued):***

***T=12h post surgery (continued):***

***60 lnc RNA identified as pre-miRNAS (ENSMUS numbers)***

***ENSMUSG00000080666***

***ENSMUSG00000065453***

***ENSMUSG00000098269***

***ENSMUSG00000098343***

***ENSMUSG00000096425***

***ENSMUSG00000098457***

***ENSMUSG00000098973***

***ENSMUSG00000072837***

***ENSMUSG00000080533***

***ENSMUSG00000094467***

***ENSMUSG00000065567***

***ENSMUSG00000104740***

***ENSMUSG00000104193***

***ENSMUSG00000084535***

***ENSMUSG00000076451***

***ENSMUSG00000084559***

***ENSMUSG00000094390***

***ENSMUSG00000092853***

***ENSMUSG00000093965***

***ENSMUSG00000092934***

***ENSMUSG00000093024***

***ENSMUSG00000095989***

***ENSMUSG00000093306***

***ENSMUSG00000096031***

***ENSMUSG00000093005***

***ENSMUSG00000096352***

***ENSMUSG00000092843***

***ENSMUSG00000092978***

***ENSMUSG00000093301***

***ENSMUSG00000094359***

***ENSMUSG00000095747***

***ENSMUSG00000093133***

***ENSMUSG00000065416***

***ENSMUSG00000065508***

***ENSMUSG00000076062***

***ENSMUSG00000065403***

***ENSMUSG00000065442***

***ENSMUSG00000076256***

***ENSMUSG00000094702***

***ENSMUSG00000093302***

***ENSMUSG00000092840***

***ENSMUSG00000104618***

***ENSMUSG00000065451***

***ENSMUSG00000096667***

***ENSMUSG00000088354***

***ENSMUSG00000098638***

***ENSMUSG00000065458***

***ENSMUSG00000070128***

***ENSMUSG00000065438***

***ENSMUSG00000065426***

***ENSMUSG00000065497***

***ENSMUSG00000076275***

***ENSMUSG00000094431***

***ENSMUSG00000096112***

***ENSMUSG00000092851***

***ENSMUSG00000094603***

***ENSMUSG00000099245***

***ENSMUSG00000076137***

***ENSMUSG00000095278***

***ENSMUSG00000076136***
