# Supplementary material for: Exploration of the Transcriptional Landscape of ALPPS Reveals the Pathways of Accelerated Liver Regeneration
Source: Front Oncol. 2019 Nov 19;9:1206. doi: 10.3389/fonc.2019.01206 (PMC6882302; doi:10.3389/fonc.2019.01206)
Supplement: Supplementary file 8 [file Data_Sheet_8.DOCX]

**Inclusion Criteria: ≥2 lncRNA/procedure; same sign per procedure**

**Exclusion Criteria: lncRNAs were exlcuded when present in both procedures (“non-specific lincRNAs”)**

List of miRNA targets in ALPPS ***(miRNA targets using mirtarbase)***

| ***miRNA*** | ***Target*** |
| --- | --- |
| mmu-let-7d-5p | Apc2, Armc2, Atp2b2, Bdnf, Cts8, Drd3, Fbxl14, Gnl3l, Ifnar1, Lins, Meis2, Msi2, Mup11, Mup17, Nf2, Nup214, Tnfrsf26, Zfp444 |
| mmu-miR-101a-3p | 4930539E08Rik, Akt1, Atxn1, B3gnt5, Bend4, Bsn, COX2, Car10, Cdc42ep3, Cebpa, Chml, Dnajb4, Dusp1, Gse1, Hdgfrp3, Hif1a, Icos, Lrig2, Map7d1, Mapre1, Msi2, Ptgs2, Ptp4a3, Rcor3, Wars2, Zfp329 |
| mmu-miR-1191a | Armc1,Bpifc,Camk4,Casc4,Ccdc85a,Dzank1,Edil3,Hecw1,Iffo2,Rab27a,Trp63,Wdr76 |
| mmu-miR-1194 | C3ar1, Car5b, Cd300a, Commd7, Dlc1, Efcab4a, Elf1, Fam154b, Gata5, Ggps1, Gtf2h2, H2T24, Hs6st2, Itsn1, Jazf1, Lpar6, March5, Mzt1, Neu3, Nip7, Nrcam, Onecut3, Osbpl8, Pctp, Ppp1r16b, Rbak, Slc35d2, Spg21, Syne2, T2, Thoc6, Tmem151a, Tmem88b, Tnfsf10, Tox4, Unc5d, Usp29, Xpo7, Ybey |
| mmu-miR-17-5p | 1600012H06Rik, 1700052N19Rik, 2510009E07Rik, 2810055G20Rik, 4930506M07Rik, 4931406C07Rik, 6030458C11Rik, A330021E22Rik, Abca1, Acap2, Acvr1b, Adam10, Adam17, Adam19, Adcyap1r1, Aff4, Ago4, Aifm1, Ajuba, Akt3, Aldh6a1, Alkbh5, Amot, Ankhd1, Ankrd17, Ankrd29, Ankrd9, Anp32a, Ap3d1, Aplp2, App, Appl2, Arap2, Arhgef9, Aspscr1, Atf2, Atxn3, B3galt2, B4galnt1, Bcl2l11, Bmp2k, Bmp4, Brms1l, Brwd1, Btg2, C1qa, C2cd4c, Cacna2d1, Cadm2, Cald1, Camk2n1, Camta1, Capn2, Casc4, Cav1, Cbx2, Ccdc88a, Ccser2, Cd164, Cd28, Cdc42se2, Cdc7,Cdh2, Cds1,Celf2, Celsr2, Cend1, Chl1, Cnot6l, Col4a2, Cops2, Cpe, Cpeb3, Cpeb4, Crebrf, Cxcl12, Cyld, Cyth1, D15Ertd621e, D4Wsu53e, D630045J12Rik, Dcbld2, Derl2, Dgkd, Dhcr24, Dhx36, Dio2, Dip2a, Dmd, Dnm1l, Dnmt3a, Dpysl2, Dpysl5, Draxin, Dync1li2, Eef2k, Elovl6, Eml1, En2, Entpd7, Ep300, Epb4, 1l5, Epha7, Eps15, Erbb4, Etnk1, Extl2, Extl3, Ezh1, F3, Fam117b, Fam120c, Fam134a, Fam134c, Fam227a, Fam49b, Fam63b, Fat2, Fbxo21, Fbxo9, Fcho2, Fchsd2, Fgf10, Fgfr1op2, Ficd, Flnb, Fn1, Foxp1, Frmpd4, Ftsjd2, Gabbr2, Gabra1, Gabrb3, Gas7, Gng4, Gpatch8, Gpm6b, Gpr63, Gramd1a, Grb10, Gtf2h2, Gxylt1, Hbp1, Hdac8, Heg1, Hid1, Hsd17b10, Id1, Ift88, Igfbp7, Igsf3, Il10rb, Ildr2, Insm1, Islr2, Itgb8, Itm2c, Kbtbd8, Kdelr2, Kdm2a, Kif21a, Kif5a, Kif5c, Klf10, Klf9, Klhl2, Klhl20, Klhl42, Kpnb1, Kras, Larp1, Lcorl, Limch1, Lmbrd1, Lnp, Lpp, Lrch1, Lrp11, Lrrc3, Lrrc55, Lrrn3, Lsamp, Luc7l3, M6pr, Macf1, Map1a, Map2, Map3k5, Map4, Map4k2, Map7d2, Mapk14, Mapre3, March6, March8, March9, Mcl1, Mcm7, Mctp2, Med17, Mef2c, Megf9, Mga, Mgll, Mlxip, Morf4l1, Mrc1, Msantd4, Msl1, Mycbp, Mylip, Myo10, N4bp2l2, Napb, Nbea, Ncam1, Necab1, Nedd4l, Nefh, Neurl1b, Neurod1, Nf1, Nfe2l2, Nfia, Nipa2, Npat, Nptx1, Nr1d2, Nrip1, Nsg2, Nt5dc3, Nudcd3, Nudt18, Ogfod1, Oprl1, Osr1, Otud4, Pabpc5, Pank1, Papolg, Pappa, Pbrm1, Pcdh10, Pcdhac1, Pcf11, Pdp2, Pfn2, Pgrmc2, Phlpp1, Pik3r4, Pim3, Plagl2, Plxna2, Polq, Polr3k, Ppap2b, Ppig, Ppp1r21, Ppp1r3b, Ppp2r2c, Ppp3r1, Ppp6c, Prune2, Pten, Ptp4a2, Ptpn11, Ptprg, Ptprj, Pum1, Pvr, Qser1, Rab11fip4, Rab12, Rab30, Rab33b, Rabgap1l, Ralgds, Ranbp2, Rap1gds1, Rapgefl1, Rasa1, Rasal1, Rasgef1a, Rassf4, Rb1, Rbl2, Rcan3, Rdx, Reep1, Rev3l, Rgma, Rnf103, Rnf213, Rnf220, Robo2, Rogdi, Rora, Rsf1, Rsrc2, Rundc3b, Ryr2, Sall3, Scara5, Scn1a, Scn2a1, Scn3b, Scn8a, Scp2, Sdccag3, Sema3c, Sepp1, Serinc1, Serpinb9, Sez6l, Sh3bgrl, Sh3d19, Shh, Shox2, Ski, Skil, Slc10a7, Slc17a7, Slc1a4, Slc24a2, Slc35f3, Slc36a1, Slc44a5, Slc7a14, Slc7a2, Smim20, Smoc2, Sobp, Socs6, Sox8, Spag9, Spast, Sqstm1, Srcin1, St6galnac5, St8sia3, Stat3, Stxbp5, Syt1, Syt11, Taok1, Taok2, Tbc1d12, Tbc1d8b, Tbx3, Tceal1, Tceb3, Tenm4, Tex2, Tfe3, Tgfa, Timp1, Timp2, Tmcc1, Tmed8, Tmem230, Tmem64, Tmod2, Tmx4, Tnfrsf21, Tnks, Tnrc6b, Tomm34, Tor1aip2, Trim2, Trip12, Trp53inp1, Tspan9, Ttc14, Ttc9, Ubr3, Ubtf, Ubxn2a, Ugcg, Usp3, Vegfa, Wdr37, Wdr82, Wfs1, Whsc1l1, Wnk1, Xpc, Xrn1, Yipf6, Ythdf3, Yy1, Zbtb41, Zdhhc16, Zeb2, Zfand4, Zfhx3, Zfp217, Zfp317, Zfp367, Zfp597, Zfp62, Zfp652, Zfp704, Zfp84, Zfpm2, Zhx3, Zic2, Zmat3, Znfx1 |
| mmu-miR-134-5p | Ccdc91, Chrdl1, Cmtm3, Creb1, Cysltr1, Dcx, Ehbp1l1, Evi2b, Fadd, Gnao1, Limk1, Lrtm2, Nanog, Nr5a2, Prkd3, Prrc2b, Pum2, Runx1t1, Shc4, Snai2, Sox2, Tmem167, Trpm3 |
| mmu-miR-1839-3p | Grb14,Hist1h1d,Myo9a,Pvrl4,Reep3 |
| mmu-miR-1839-5p | 2410012M07Rik, Akap8, Azi2, Ccdc157, Ecm2, Eif2s1, Fam211b, Fli1, Glt28d2, Il7, Mfsd4, Neu4, Ric8b, Slc11a2, Slc25a42, Tnfrsf26, Ttll1 |
| mmu-miR-18a-5p | Abce1, Hsf2, Kif1a, Lrrc58, Map3k1, Pten, Rab11fip1, Runx1, Smad2 |
| mmu-miR-1906 | 4930444A02Rik, Angel1, Armcx6, Cdc14b, Cox15, Ddx19b, Dhdh, Fam168b, Fbxo21, Fgd4, Irgq, Isoc1, Itgav, Itsn1, Klc1, Mapkap1, Mbtps2, Mtf1, Opa1, Otop1, Oxsm, Pacsin2, Sorcs2, Spen, Spsb4, Tacc1, Tln2 |
| mmu-miR-19a-3p | 1700019G17Rik, Acadm, Akap2, Atp10a, Atxn1, Chrnd, Cml2, Fam104a, Fam83d, Fgfr1op2, Gja3, Ilf3, Kdm2a, Map3k12, Nhsl1, Pten, Rnf11, Slc35f1, Snap25, Snx27, Tet2, Timp2, Tmem25, Tnf, Ttc39b, Ubap2l, Zfpm2 |
| mmu-miR-19b-3p | 1500009C09Rik, 1700019G17Rik, 2510039O18Rik, 4931406P16Rik, 5730455P16Rik, Abcg1, Abhd10, Abi2, Abl2, Acadm, Actn1, Acvr1b, Adam11, Adam23, Adcy1, Adcyap1r1, Add3, Aff4, Agap1, Agfg1, Aicda, Akap2, Akap7, Akap9, Aldh3a2, Amz2, Ankib1, Ankra2, Ankrd26, Ap3s2, Arap2, Arel1, Arf3, Arhgap21, Arid4b, Arl6ip1, Arpc3, Asxl3, Atf2, Atp10a, Atp2a2, Atp2b2, Atp6ap2, Atp6v1b2, Atp8a1, Atraid, Avl9, B3galnt1, B4galt6, Btbd7, C1galt1, Cadm2, Cadm3, Camk2n1, Camkk2, Camsap2, Caprin1, Cav1, Cblb, Cbln2, Ccser2, Cds1, Celf1, Cep120, Chrnd, Chst1, Clock, Cltc, Cml2, Cmtm6, Cnih, Cnksr2, Cnot1, Cnot6, Cnr1, Cox5b, Csmd1, Daam1, Dcbld2, Dennd5a, Diras2, Dock4, Dpp6, Dpysl2, Drd1a, Dsel, Efnb2, Eif4g2, Enc1, Enpp4, Enpp5, Entpd7, Eogt, Ercc6, Etnk1, Ezh1, Fam104a, Fam126a, Fam173b, Fam179b, Fam20b, Fam83d, Fat3, Fbxo8, Fem1b, Fgfr1op2, Fkbp1b, Fndc3b, Foxo3, Ftsjd2, Furin, Fzd6, Gadd45a, Gja3, Gmeb2, Gmpr, Gnai1, Gng12, Golt1b, Gpcpd1, Gpr155, Gramd1b, Grb10, Grin3a, Gtdc2, Gtf2i, Hecw2, Hg1, Hif1an, Hipk3, Hlf, Hmgcll1, Hpca, Hprt, Hs2st1, Igfbp3, Ilf3, Inhbb, Itga6, Jtb, Kcnab3, Kcnj10, Kdm2a, Kif21a, Kif21b, Klf7, Ldhb, Ldlr, Ldlrad4, Lemd3, Lhfpl2, Lman1, Lnpep, Lonrf1, Lrp11, Maml1, Map3k1, Map3k12, Mfsd7c, Mid2, Mink1, Msantd4, Mtmr12, Myh9, Mylip, N4bp1, Naa50, Nap1l3, Nbea, Ncald, Ndfip2, Neurod1, Nfia, Nfya, Nhsl1, Nipal3, Npnt, Npy2r, Nrarp, Nsg2, Nudt10, Oprl1, Pak3, Pank1, Papss2, Paqr8, Parg, Pde7b, Pea15a, Phf3, Phtf2, Pianp, Pigl, Pik3r3, Plagl2, Ppap2b, Ppp2r2a, Ppp2r5e, Pros1, Prrc2c, Prrg3, Prune2, Psme4, Pten, Rab11b, Rab21, Ralbp1, Ralgps1, Rapgefl1, Rasgef1a, Rasgrp1, Rassf8, Reps2, Ric3, Rin2, Rnf11, Rnf111, Rnf128, Rnf167, Rnf216, Rnf38, Rnf44, Robo1, Rph3a, Sall3, Scamp5, Sema5a, Sfrs18, Sgcb, Sh3d19, Sh3rf3, Shank1, Sik1, Skil, Slc17a7, Slc24a2, Slc24a3, Slc35b4, Slc35f1, Slc35f3, Slc36a1, Slc6a8, Slc7a5, Slc9a2, Slitrk5, Smarca2, Smoc2, Snap2, Snip1, Sntb2, Snx27, Snx30, Socs6, Soga1, Soga3, Sp1, Srebf2, Srrm2, Sstr4, St8sia3, Stam, Stard13, Stat1, Stim2, Stk35, Suv420h1, Syt1, Syt11, Tbc1d12, Tbc1d23, Tef, Tet2, Tgif1, Timp2, Tm4sf1, Tm9sf3, Tmem179, Tmem245, Tmem25, Tmem30a, Tmem57, Tmod2, Tnip1, Tnks, Tnpo1, Tnrc6b, Trappc10, Trib2, Tspan2, Tspyl2, Ttc3, Ttc39b, Ttyh3, Tub, Txnip, Ubap2l, Ube3b, Ubl3, Ubr4, Ubxn4, Usp33, Vcpip1, Whsc1l1, Wnk1, Wnt1, Xxylt1, Ywhab, Zcchc2, Zeb2, Zfp120, Zfp275, Zfp41, Zfp521, Zfp597, Zfp800, Zfpm2, Zhx3, Zkscan1, Zmym2, Znfx1 |
| mmu-miR-20a-5p | Ago4, App, Bmp4, Cd28, Ep300, Fam227a, Fgf10, Fgfr1op2, Hbp1, Id1, Il10rb, Kdm2a, Larp1, Mapk14, Mctp2, Mef2c, Morf4l1, Nedd4l, Neurl1b, Osr1, Pcdh10, Pdp2, Ppp1r3b, Pten, Ptp4a2, Shox2, Sqstm1, St8sia3, Stat3, Tbx3, Timp2, Trp53inp1, ULK1, Ubxn2a, Vegfa, Zbtb41, Zbtb7a, Zfpm2 |
| mmu-miR-30c-5p | Amer1, Camk4, Ccne2, Cd300a, Cdk13, Celsr3, Cep170b, Csf1, Dpy19l1, Egfr, Eno2, Fam227a, Gskip, Hoxb3, Il18r1, Ing1, Ints12, Lpar3, Mdm2, Mesdc2, Mgea5, Mtdh, Narg2, Nfatc3, Pgr, Pigh, Ptrhd1, Reep4, Rrad, Runx1, Serpine1, Six4, Slc16a1, Smad1, Snai1, Strip1, Tbc1d5, Timp3, Tnrc6b, Tomm70a, Trp53rk, Twf1, Wdr44, Wdr89, Zfp446, Zfp711, Zfp9 |
| mmu-miR-3470a | 2010109I03Rik, A630033H20Rik, Abhd15, Acmsd, Acp2, Ap5m1, Arid4b, BC030476, Btaf1, Calm1, Camsap1, Cand2, Clmn, Crtc1, Csf3r, Cx3cr1, Denr, Dut, Elmod3, Epg5, Erich1, Fam134b, Fam86, Fbxl3, Fcer2a, Fktn, Ftsj1, Gad2, Gla, Gprc5c, Ikbip, Il5ra, Invs, Itih5, Jakmip2, Kctd14, Ky, Lgr5, Limd2, Lrrc14b, Lrrc8e, Lsm11, March1, Med16, Mettl2, Mfap1a, Mob4, Mobp, Mrpl19, Myo1e, Nek7, Npy1r, Nwd1, Pax2, Pof1b, Prex2, Prickle2, Prss43, Ptpn7, Rangap1, Raph1, Rhag, Rint1, Samd4, Scd2, Slc17a8, Slc35g1, Slc7a2, Slx1b, Smyd4, Snx30, Srgn, Tmed5, Tmem170b, Tmem178b, Tmem50a, Tmppe, Trim39, Ttbk2, Ung, Wbp1l, Wdr59, Zfp397, Zfp459, Zfp617, Zfp959, Zfyve27 |
| mmu-miR-3470b | 2010012O05Rik, 2510002D24Rik, 4930579G24Rik, 5730455P16Rik, A130010J15Rik, AY074887, Abcd4, Adam28, Afg3l1, Aida, Aoc3,Ap5z1, Arhgef9, Asxl2, Bckdk, Camk4, Car5a, Ccdc78, Cd300a, Cd300lf, Cd8b1, Chek2, Chrnb4, Ciapin1, Clmn, Cog7, Crtc3, Ctsa, Dbt, Dcp2, Ddx55, Denr, Dkc1, Dlgap5, Dnal1, Dtx3l, Eci1, Elf1, F10, Fbxo22, Fsd1l, Fxn, Gabpb2, Gga2, Gimap1, Glrx2, Gm4951, Gnal, Gng2, Hccs, Il11, Inip, Iws1, Kcna4, Krr1, Lhx9, Lmbrd1, Lrrc3, Lypla1, Madd, Mau2, Mettl2, Mob3c, Mpv17, Mtrr, N6amt1, Nav1, Nbl1, Ncl, Ncoa7, Nfatc2ip, Nol10, Opa3, Pik3c2a, Pla2g2d, Pla2g7, Polr3f, Prdm2, Psmb11, Psmd9, Rab27a, Rad51c, Rapgef4, Rnf168, Rp2h, Rpl23, Rpl7l1, Rprd1a, Samd8, Sap18, Sass6, Serpine1, Sfn, Sgpp2, Slc10a1, Slc23a1, Slc2a10, Slc6a2, Smurf2, Sppl2a, Svopl, Taf1, Taf1a, Tcea1, Timm17b, Tmem245, Tns4, Tprkb, Tspyl5, Ttc9c, Uvssa, Veph1, Vps72, Vsx1, Was, Xcr1, Ybx1, Zfp111, Zfp553, Zfp78, Zfp882, Zfp941, Zmym2, Zyg11b |
| mmu-miR-3471 | 2310030G06Rik, 9930104L06Rik, Aak1, Adam19, Akr1d1, Anapc16, Ankrd28, B4galt6, Casp8, Cd300a, Cd8b1, Chrnb4, Cnih4, Commd7, Cpm, Cxcr7, Dbt, Dennd4c, Ggps1, Glrx2, Glyctk, Gmip, Gnal,H2-T24, Heatr5a, Idi2, Ifngr2, Il1rl1, Ints6, Iws1, Limd1, Llph, March5, Neu3, Pigh, Ppp1r16b, Psmb11, Ptcd1, Ran, Rapgef4, Rnf150, Rprd1a, Rragd, S1pr3, Slc12a8, Slc35d2, Tcaim, Tmem151a, Tmem69, Tmem88b, Tnfsf10, Trp53rk, Ubxn2a, Urb2, Uvssa, Whsc1, Ybey, Zbtb9, Zfp329 |
| mmu-miR-377-3p | 2810407C02Rik, Acot11, Acvr1b, Adam19, Adamts9, Adra2b, Agpat4, Anln, Arf3, Arhgap23, Arhgef15, Arhgef9, Arid4a, Arnt, Arxes2, Atf2, Atg16l1, Atl2, Atm, Atxn7, BC068281, Bbc3, Bsn, Cadm2, Cadm3, Capza1, Cbln3, Ccdc138, Ccrl1, Cd1d1, Cd3d, Cisd2, Clasp1, Clec4a2, Clic5, Coa5, Cog7, Creb5, Cry2, Cstf3, D16Ertd472e, Dcp2, Dzank1, E2f1, Ehf, Ehmt1, Elovl7, Ephb3, Ephx3, F11r, Fam114a1, Fam160b2, Fam212b, Fam53c, Fbxl17, Frmd3, Fubp1, Gad2, Gmeb1, Gpd1l, Gxylt1, Gxylt2, Hexim1, Hnrnpd, Hook3, I830012O16Rik, Ifit3, Igdcc4, Igfbp3, Iglon5, Ikzf2, Ino80d, Insig2, Ints8, Iqsec3, Itgam, Itsn1, Jazf1, Kcna4, Krtap4-9, Lcorl, Lman2, Lonrf1, Lrp8, Mbtps2, Mmab, Mob3c, Myrip, Nceh1, Net1, Nfatc4, Ociad2, Pabpc4l, Pacs2, Pak4, Pappa, Pax3, Pck1, Pdpk1, Pfkfb2, Pgbd1, Phactr2, Pi15, Plcl1, Plekha2, Plekhg1, Ppp1r2, Ppp1r26, Prr3, Prr5, Prrg3, Prrg4, Pttg1, Rapgef3, Rasal2, Rcan2, Rictor, Rmi2, Rnf152, Rnf219, Rnf41, Rsf1, Scamp1, Sele, Sema4f, Sema6d, Sept11, Sfn, Shroom1, Sin3a, Slc1a2, Slc35a1, Slc36a1, Slc43a2, Slc7a11, Smarca4, Spock2, Spp1, Spryd3, Tbl1xr1, Tcf15, Tmem170b, Tmem201, Tmem65, Tmem8b, Tmlhe, Tnfrsf1b, Tnfsf10, Trak1, Trp53i11, Tulp3, Ubn2, Ubtf, Ulk3, Vdr, Wdr59, Xpnpep3, Zbtb7a, Zdhhc24, Zdhhc7, Zfp467, Zfp532, Zfp663, Zfp689, Zfp827, Zfp839 |
| mmu-miR-410-3p | 1200011I18Rik, 4921524J17Rik, Acbd5, Akna, Anln, Arhgap11a, Arl15, Atp2b2, Cacnb2, Calm1, Casc4, Cbx3, Ccdc90b, Cd69, Ceacam1, Cops7b, Crebzf, Crp, Ctxn3, Cxxc5, Dimt1, Dlx3, Eif2s1, Elavl4, Ep300, Erc1, Etv3, Fam151b, Fam46a, Fbxl20, Fgf16, Folh1, Fsd1l, Fzd1, Fzd5, Gm8369, Gpc6, Grhl3, Has2, Hexim1, Hiat1, Hist1h1d, Hopx, Hoxa11, Id2, Igfbp3, Ikzf5, Ints6, Ism1, Klf12, L3mbtl4, Larp1, Lin54, Lrig2, Lrrc71, Lrrtm2, Lzts3, Map1a, Mettl20, Mex3b, Mgea5, Mllt3, Mpped2, Nadsyn1, Nck2, Nckap5l, Ndnf, Nf2, Nfib, Nln, Nol7, Ntrk3, Nupl2, Ocrl, Olr1, Osbp, Pcdh20, Pcdhb17, Pcsk5, Pfkl, Phf17, Pkib, Plau, Plaur, Plxnc1, Pm20d2, Pou4f2, Ppp4r2, Ppp6r1, Prrx1, Pten, Ranbp10, Rasal2, Rassf5, Rbms3, Rccd1, Reep5, Ret, Rnf166, Rnf6, Rragb, Rufy2, Runx1t1, Senp3, Shh, Slc12a8, Slc35a1, Slc7a11, Slc8a1, Slitrk2, Smurf2, Snx27, Sort1, Sox11, Sri, Strbp, Synpr, Syp, Tbx4, Tcf21, Tcf7l2, Tead3, Tet2, Tex12, Tfam, Timp3, Tmem100, Tmem161b, Tmem56, Trak2, Trpc7, Trps1, Ttc17, Ttc39b, Ugt2b35, Usp6nl, Whsc1l1, Wnt11, Wnt3, Wwtr1, Zbed6, Zc3hav1l, Zer1, Zfand5, Zfp148, Zfp384, Zfp518b, Zfp644, Zmynd8, Znrf1, Zwint |
| mmu-miR-466i-3p | 1700028K03Rik, 2900026A02Rik, 4933427D06Rik, 9930021J03Rik, A430078G23Rik, A630001G21Rik, AK010878, Aak1, Abca6, Acot11, Acvr1b,Adamts9, Adcyap1r1, Adm, Adrbk2, Akap10, Akap5, Alkbh1, Alkbh5, Ankle1, Anln, Aplnr, Apol8, Appl1, Arf3, Arhgap12, Arhgap32, Arl14epl, Arl4c, Arl5b, Arl8a, Armc2, Atg10, Atxn1, Atxn7, Azi2, B3gnt2, B4galt6, BC030336, Bbc3, Bcl2l11, Bcl2l15, Bpifc, Bsn, Btnl9, C2cd4c, Cadm3, Ccdc121, Ccdc144b, Ccdc169, Ccpg1, Cdc14a, Cdc37, Cdk12, Cebpa, Cecr6, Cep170b, Cep76, Chek1, Chic1, Chrna1, Clec7a, Cnr1, Col24a1, Col8a1, Cops7b, Crim1, Cry2, Csnk1e, Csrnp3, Cst6, Ctif, Ctsl, Cxcl5, Cyb5r1, Cytip, D230025D16Rik, D630023F18Rik, Dcaf17, Dcun1d1, Ddit4l, Ddx55, Decr1, Dennd4a, Desi2, Dhx37, Diap2, Dnajb1, Dnajb4, Dnajb6, Dpm1, Dus3l, Dusp18, Dusp9, Dzank1, Dzip3, E2f1, Efnb2, Ehhadh, Eif2s1, Eif4e2, Epha4, Erc1, Ermp1, Ets1, Evi2b, Eya1, Fam101b, Fam107a, Fam160b2, Fam168b, Fam169b, Fam212b, Fam214b, Fam227a, Fam46a, Fam53c, Fam78b, Fat3, Fbxl14, Fbxl17, Fech, Fgf5, Fhod1, Fign, Fndc1, Fndc3a, Foxj3, Foxn3, Frat2, Fus, Gabpb2, Gcnt4, Gfod1, Gm13476, Gm1587, Gm5148, Gmeb1, Gnb4, Gnl3l, Gpcpd1, Gpd1l, Gpkow, Gpr20, Grb2, Grik3, Grin2b, Gtf2h2, Gxylt1, Haus2, Hecw1, Hif1an, Hif3a, Hist1h1d, Hivep3, Hk2, Hook3, Hrk, I830012O16Rik, Ids,Ifitm7, Igf1, Ikzf2, Il15ra, Il17ra, Il18r1, Ints6, Ints8, Irgq, Itpripl2, Jag2, Jhdm1d, Kcnc1, Kctd11, Klf8, Klhl13, Klra2, Larp1, Ldb3, Ldlrad3, Lhfpl4, Lin28b, Lmx1a, Lnp, Lonrf1, Loxl3, Lrp12, Luc7l2, Lzts3, Map3k7, Mast3, Mast4, Mavs, Mcam, Mdga1, Mef2a, Mfsd5, Mill1, Mkx, Mmp12, Mms22l, Mrap2, Mrps24, Ms4a4b, Ms4a4c, Msr1, Msx2, Mtfr2, Mtx3, Mylk, Nacc2, Nbeal1, Ndrg2, Nfatc2, Nkx1-2, Npy1r, Nrf1, Nrg3, Nrp2, Nufip2, Nup133, Oas1e, Oas3, Ociad2, Oscp1, Otog, Pak6, Pappa, Pard6b, Pbld2, Pcdh15, Pcgf2, Pck1, Pde4dip, Pfas, Pgbd1, Pgm2l1, Phactr2, Phtf2, Pias1, Piga, Pik3r1, Plag1, Plau, Plcl1, Plxna4, Pofut1, Ppp1r14c, Prdx1, Prkcd, Prps2, Prr5, Prrg3, Prrx1, Prss22, Pter, Ptp4a2, Ptprk, Rab3c, Rabgap1, Rad51d, Rad54l2, Rap2b, Rbbp5, Rdh1, Rgl1, Rgs20, Rnasel, Rnf152, Rnf157, Rock2, Rpp14, Rraga, Rtf1, Rufy2, Rwdd4a, Scg2, Scrt2, Sept11, Sept3, Serpinb8, Set, Sgms1, Sin3a, Six4, Skint7, Slc10a2, Slc11a2, Slc12a2, Slc16a10, Slc25a46, Slc25a51, Slc41a3, Slc6a17, Slc6a8, Slc7a11, Slfn5, Slx1b, Smarca4, Snhg11, Sort1, Sox7, Spag7, Spata13, Spta1, Srgap2, Srp72, Srxn1, Ss18l1, Ssr1, St6galnac1, Ston2, Strbp, Stx1b, Stxbp6, Synj2bp, Synpo2l, Syt12, Syt2, Sytl4, Tagap, Tagap1, Tas1r3, Tbc1d13, Tbc1d22b, Tbl1xr1, Tbx20, Tcf4, Tcte1, Tdrd1, Thsd4, Tll1, Tmbim1, Tmem178b, Tmem201, Tmem252, Tmem26, Tmprss13, Tnfrsf11a, Tns1, Tns3, Tpm3, Tpte, Tril, Trim12c, Trim44, Tspan31, Tyw1, Ubac1, Ubap2l, Ube2v2, Ubn2, Ugt2b35, Uhrf1bp1l, Unc5a, Unc5c, Usf2, Usp27x, Vegfa, Vps4b, Vps72, Wfdc12, Wfs1, Whsc1l1, Xiap, Xk, Zbtb10, Zbtb34, Zbtb39, Zbtb42, Zbtb7b, Zc3h12d, Zdhhc5, Zfhx3, Zfp128, Zfp169, Zfp354a, Zfp408, Zfp462, Zfp516, Zfp827, Zfp839, Zfp871, Zfp874b, Zfp882, Zfp951, Zfp955a, Zfp963, Zscan4c, Zscan4d, Zxdc |
| mmu-miR-466i-5p | 1700019G17Rik, 4921509C19Rik, Abcc9, Acot2, Acsm2, Acss1, Adamts14, Adamts17, Adarb2, Ado, Adra1b, Adrbk2, Agtrap, Akap10, Akap2, Akap7, Ank2, Ap1ar, Apba1, Aplnr, Appl1, Aptx, Asah2, Asap3, Asb13, Ascl4, Asxl2, Atp2b3, Atxn1, B4galt6, BC053749, Barhl2, Baz2b, Bcl2, Bcl2l11, Bend3, Bnc2, C030039L03Rik, C5ar2, Cacna2d2, Calcoco1, Calcr, Camk1d, Cap2, Car10, Casc4, Cask, Casp8, Ccdc85a, Ccna2, Ccpg1, Ccrl1, Cd1d1, Cd274, Cd28, Cd2ap, Cd33, Cd4, Cdh12, Cdh20, Cdk13, Cdk7, Cds2, Ceacam1, Ceacam18, Cer1, Chrnb4, Chrnd, Chst2, Clec7a, Clvs1, Cml2, Cnih3, Cnksr2, Cntn3, Commd7, Cox15, Cradd, Creg2, Csf2ra, D430042O09Rik, D630003M21Rik, D630045J12Rik, Ddx6, Dgkg, Dhdh, Dhfr, Dmd, Dmrta1, Dnase1l3, Dnlz, Draxin, Drp2, Dzank1, E2f8, Ebf3, Ece1, Efcab14, Egfl6, Ehd3, Ehhadh, Elfn1, Elovl6, Entpd1, Eogt, Epas1, Epha4, Epha7, Etv3, Evi2b, Fam107a, Fam169b, Fam198a, Fam213a, Fblim1, Fbrs, Fbxl17, Fech, Flnb, Flrt1, Foxk1, Frk, Frmd5, Fsd1l, Gabpb2, Gatc, Gcnt4, Gdpgp1, Gfod1, Gfpt1, Gfra2, Gm14137, Gm14326, Gm4841, Gm5615, Gna13, Gnal, Gnb4, Gpr123, Gramd1c, Grasp, Greb1, Gria3, Grid1, Grik3, Gtf2h2, Has2, Havcr2, Heatr2, Hif1an, Hoxc8, Hpca, Hspb7, Htt, Iars, Iffo2, Ifi44, Igf1r, Igf2, Iglon5, Igsf11, Il18r1, Il8, Ildr2, Insig2, Insr, Iqgap2, Itga11, Itga9, Jarid2, Kank2, Kcnc1, Kcnip3, Kcnj1, Kcnj16, Kif1a, Klhl13, Klhl23, Krt222, L1cam, LOC100048884, Lbp, Lcp2, Lhx6, Lifr, Lin7a, Lnp, Lpp, Lrrc32, Lrrc61, Lrrn4cl, Lrtm2, Ltf, Ly6g6c, Ly96, Maf, Mafb, Magee2, Man1c1, Man2a2, Map3k7, Mapk11, Mapkbp1, Mavs, Mbnl3, Mcidas, Mdm2, Mfap3l, Micall1, Mlec, Mocs1, Mon1b, Mrgpre, Ms4a5, Mtmr12, Muc13, Mylk4, Nab1, Nab2, Napb, Nat8l, Nbeal1, Ncam1, Ncan, Nedd4l, Neu1, Neurod2, Nfat5, Nfatc1, Ngfr, Nid1, Nkap, Nkx2-9, Nmnat2, Npr3, Nqo2, Nr4a2, Nrbp2, Nsun3, Nwd1, Nxpe3, Oacyl, Onecut2, Opcml, Oprm1, Oxsm, Oxtr, P4ha3, Pacsin2, Palm2, Pappa2, Pcdh10, Pcdh17, Pclo, Pdcd4, Pdxk, Pgm5, Phactr3, Pik3r5, Pirt, Plcb1, Pld5, Podn, Pogk, Ppp1r16b, Ppp1r1c, Prdm12, Prdm8, Prex2, Prkcd, Prkci, Prrc2b, Prss42, Psd2, Psd3, Pstpip2, Ptgdr, Ptprb, Ptpre, Pura, Rab11fip1, Rab3c, Rab6b, Rab9b, Rabgap1, Rara, Rasa2, Rasal2, Rassf2, Reps2, Rfx3, Rgs9bp, Rhobtb1, Rhou,R nasel, Rorb, Rpusd2, Rsph4a, Runx1, Runx1t1, Runx3, Saa4, Samd7, Scd3, Scn2a1, Sema5a, Sema5b, Sema6a, Sfrp1, Sh2d2a, Shisa6, Shroom3, Sike1, Sim1, Six4, Ski, Slc17a5, Slc1a2, Slc22a8, Slc24a2, Slc25a12, Slc25a21, Slc30a10, Slc31a2, Slc39a14, Slc4a4, Slc5a8, Slc6a17, Slc6a6, Slc7a1, Slc8a1, Slc8a3, Slmo1, Smarca2, Smo, Smpd4, Snai2, Snap23, Snap25, Snx12, Sox1, Sp9, Srgap3, Ssr1, St18, St8sia1, Steap2, Stk10, Stxbp4, Stxbp5l, Supt7l, Suv420h1, Syn3, Synj2bp, Syt15, Tacr1, Tacr2, Tbc1d30, Tbx15, Tbx22, Terf2ip, Tet2, Tfap2b, Tgfb2, Thsd4, Tiprl, Tmco1, Tmem132b, Tmem151b, Tmem236, Tmem245, Tmem26, Tmem47, Tmod2, Tnfrsf13c, Tns4, Trim65, Trp53i11, Trpc7, Trpm3, Tspan18, Tyw3, Ubtf, Uhrf1bp1l, Unc13b, Unc5d, Unc93a, Uncx, Urb2, Vdr, Vprbp, Vps33b, Vps37a, Vsnl1, Wars2, Wdr46, Wrn, Xk, Xpr1, Xrcc3, Zeb2, Zfand2a, Zfhx3, Zfp169, Zfp248, Zfp329, Zfp39,Zfp449, Zfp46, Zfp488, Zfp641, Zfp68, Zfp691, Zfp74, Zfp92, Zfp931, Znrf3, Zscan29 |
| mmu-miR-675-3p | Ccdc127, Ell2, Fam214b, Flrt1, Il18r1, Lrrc40, Myo1e, P4ha3, Prr11, Pstpip2, Rab9b, Sema5a, Sema5b, Slamf1, Slc15a5, Slc6a6, Tmco1, Vdr, Whsc1, Wnt7a, Zc3h12a, Zfp46 |
| mmu-miR-675-5p | Dnase1l3, Ky |
| mmu-miR-485-5p | Cd93, Foxk2, Gns, Pax2, Pigm |
| mmu-miR-703 | 4933427D06Rik, Arhgap11a, Asxl2, Braf, Cep97, Cnnm3, Cnot4, Commd8, Epas1, Faah, Fam96a, Foxj3, Git2,Gpr3, Il1r1, Ilkap, Kif23, Larp4b, Mars2, Mau2, Mmab, Mnat1, Mxra7, Narf, Pcnp, Pctp, Pde8b, Pdxk, Phex, Pitpnm2, Prex2, Prr18, Prtg, Ptcd1, Rem1, Ret, Ric3, Rnf165, Rpp14, Rras2, Rsf1, Sept8, Slc12a2, Slc25a51, Slc30a7, Tcf7l2, Tctn3, Tlx2, Tmem170b, Ttc17, Ttc32, Usp29, Usp43, Vps26a, Xpnpep3, Zbed4, Zbtb14, Zfp106, Zfp11, Zfp53, Zfp84 |
| mmu-miR-770-3p | 2510002D24Rik, Cd8b1, Pctp, Syt7, Tmem176b, Zfp941 |
| mmu-miR-770-5p | BC027231, Chm, Cisd2, Gm14420, Lsm6, Pigt, Tmem178b, Tmlhe,Ttc38 |
| mmu-miR-92a-3p | Btg2, Cd69, Chm,Cpeb1, Fam136a, Gm14420,Gm5148, Hipk3, Il5ra, Map2k4, Mylip, Ncam2, Rhbdl3, Shox2, Stk10, Tagap, Tagap1, Tbx3, Trp63, Uba1y, Zfp300, Zfpm2, Zpbp |
