# Supplementary material for: Exploration of the Transcriptional Landscape of ALPPS Reveals the Pathways of Accelerated Liver Regeneration
Source: Front Oncol. 2019 Nov 19;9:1206. doi: 10.3389/fonc.2019.01206 (PMC6882302; doi:10.3389/fonc.2019.01206)

1) TRAF pathway


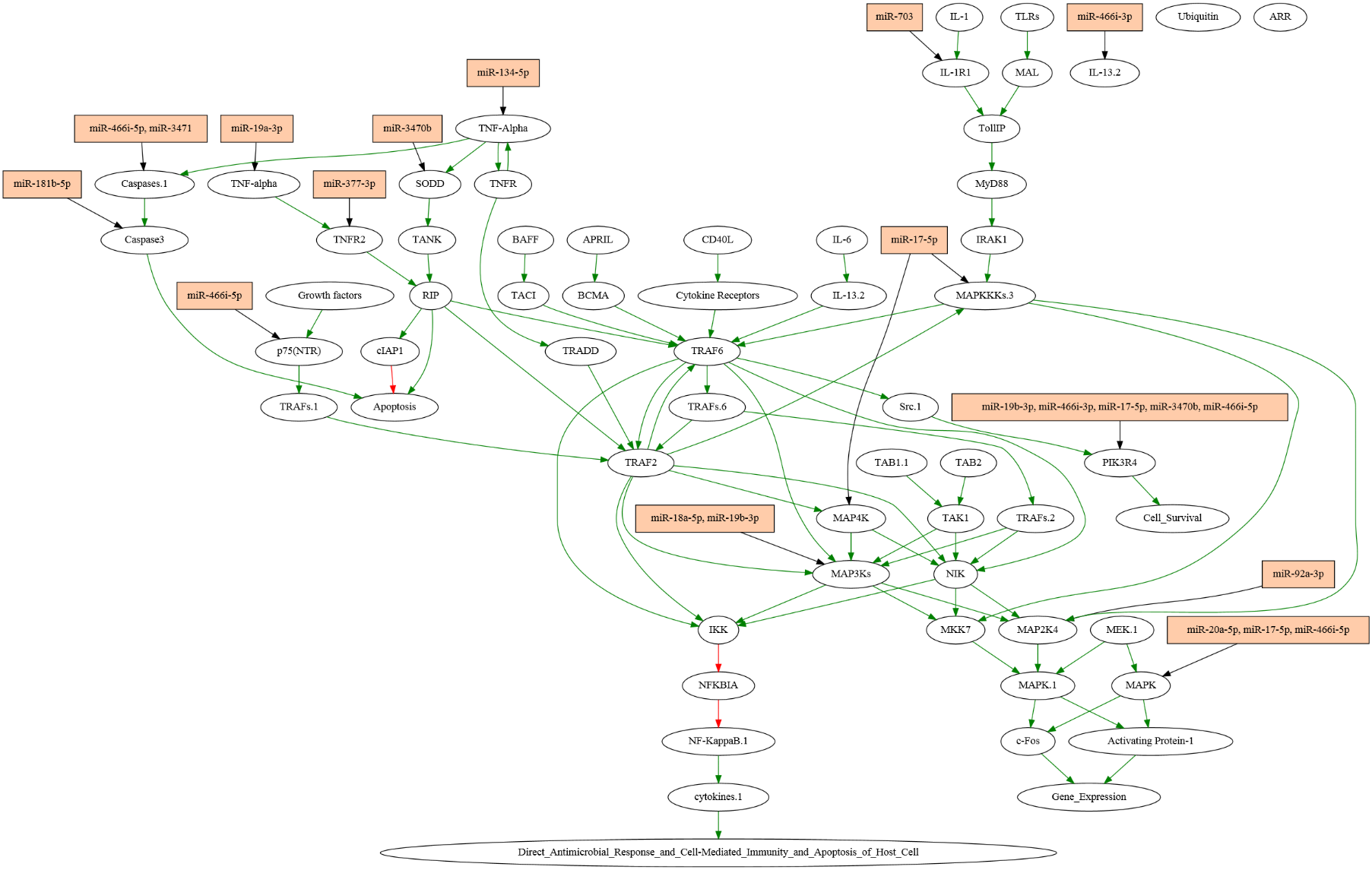


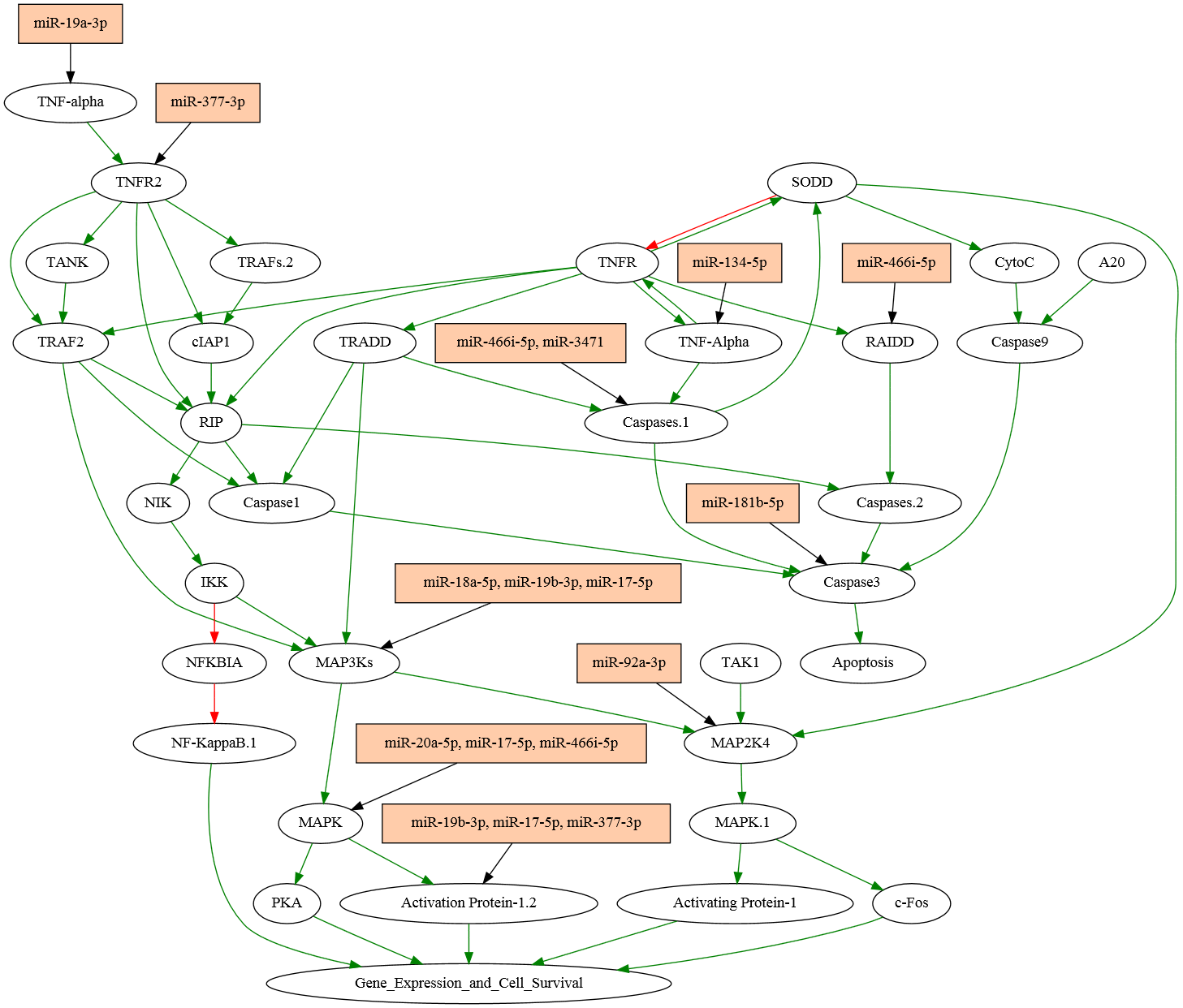
2) TNF pathway


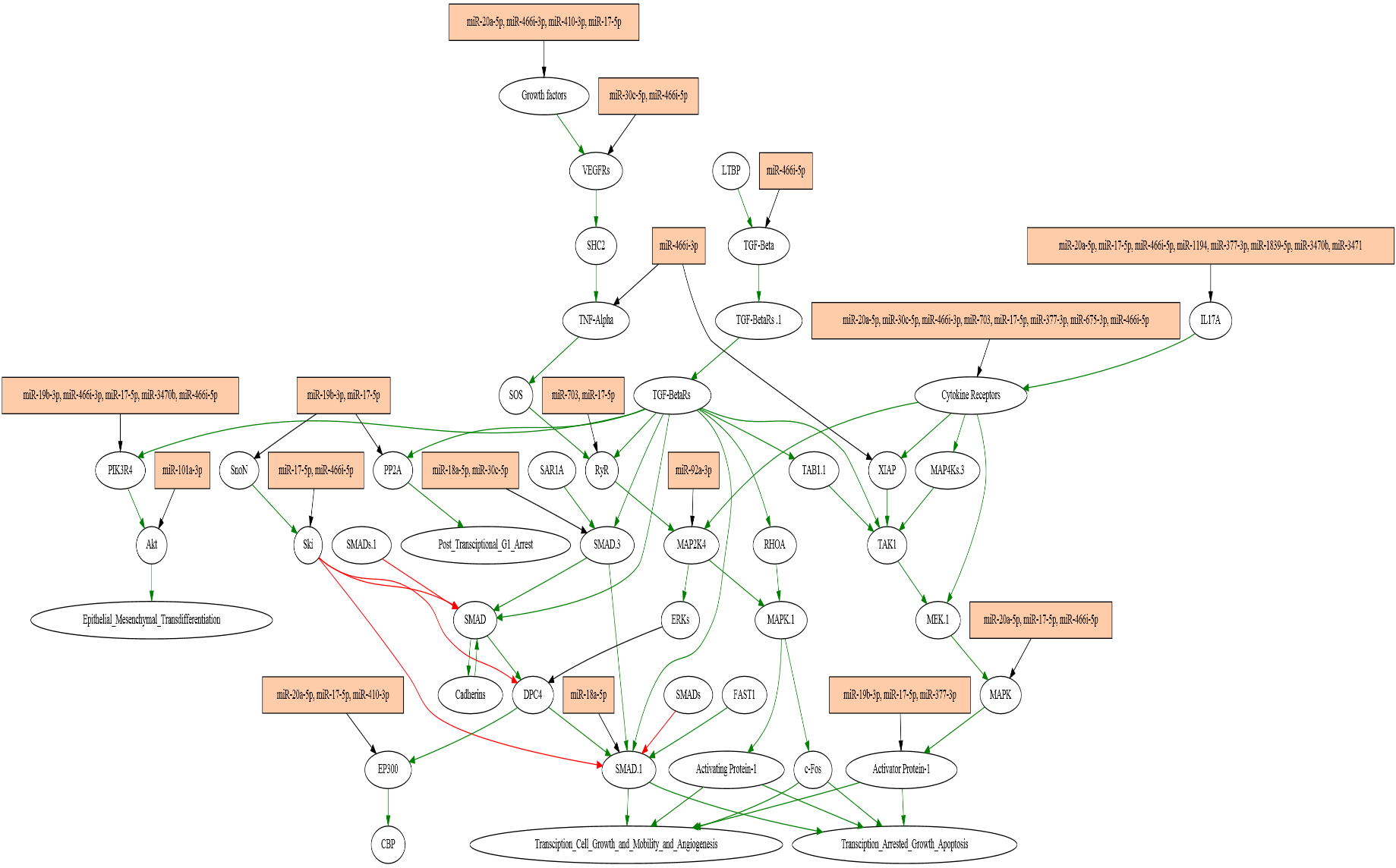
3) TGF-beta pathway

4) PAK pathway (Lamelliopodia_and_Filopodia_Outgrowth)


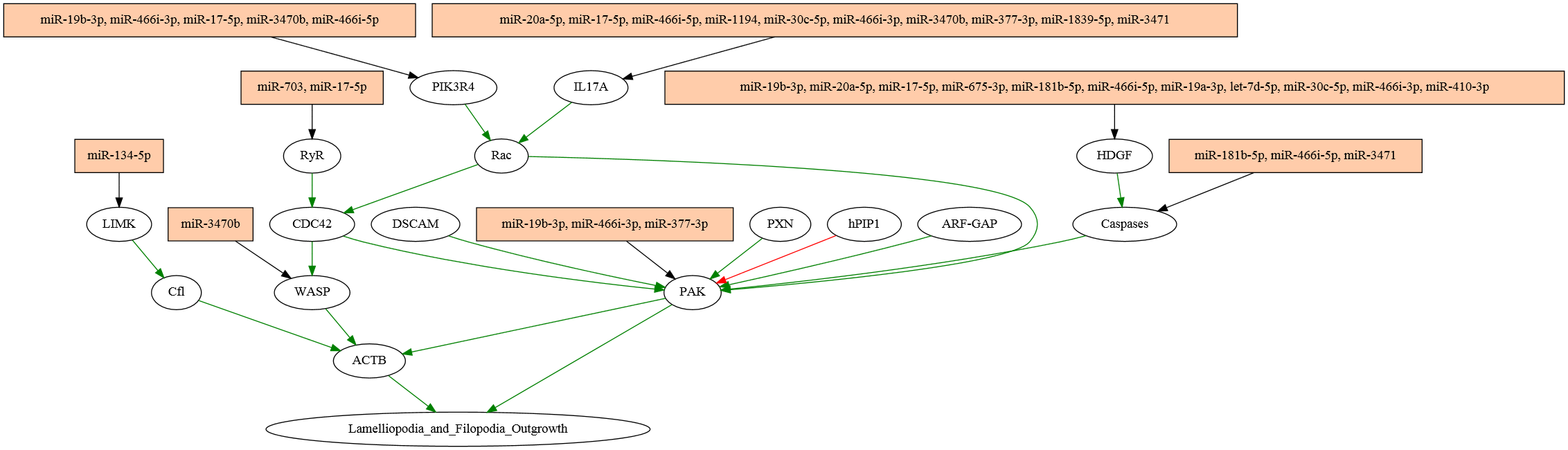


5) PAK_Pathway (Cell_Survival)


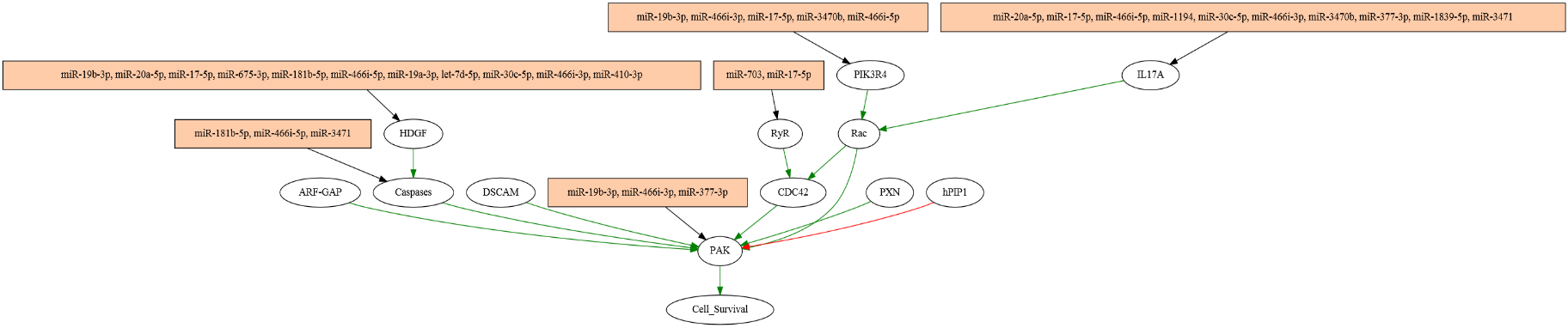


6) PAK Pathway (Actin_Organization)


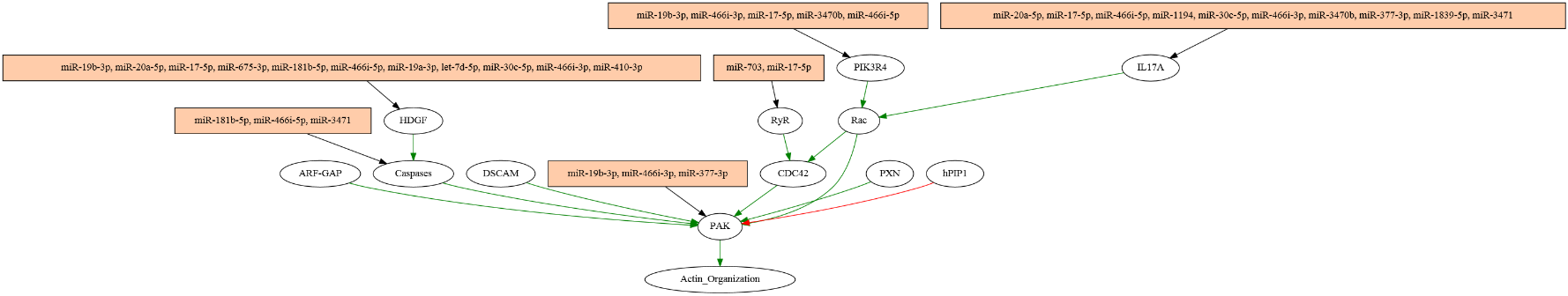


7) PAK Pathway


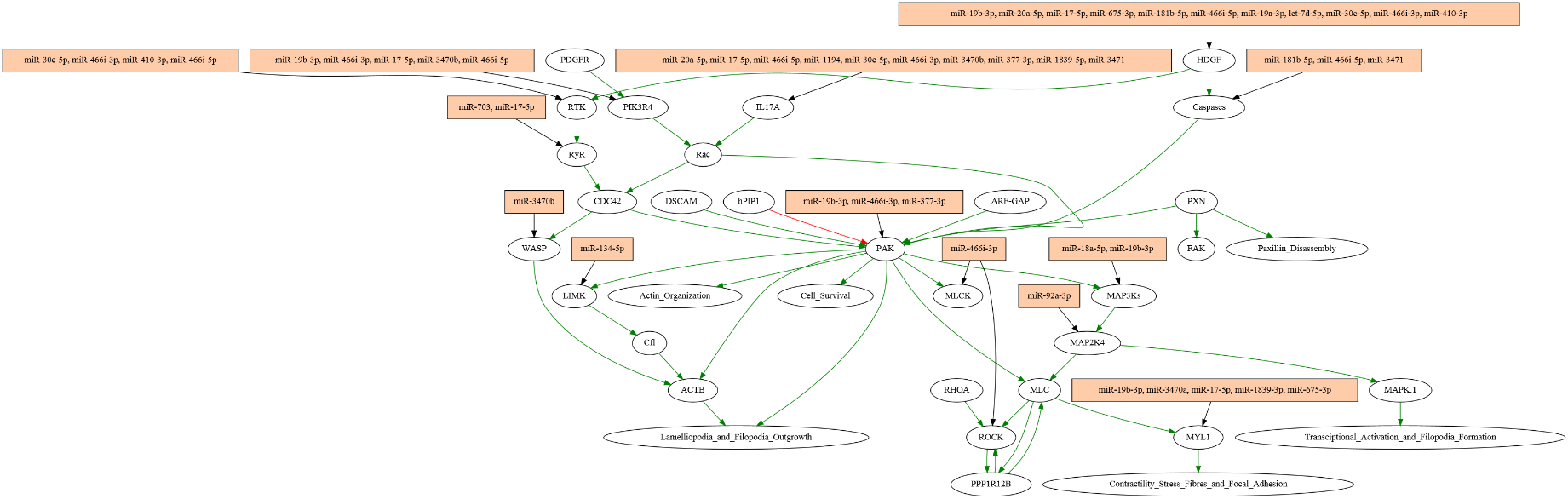


8) p38 signaling pathway


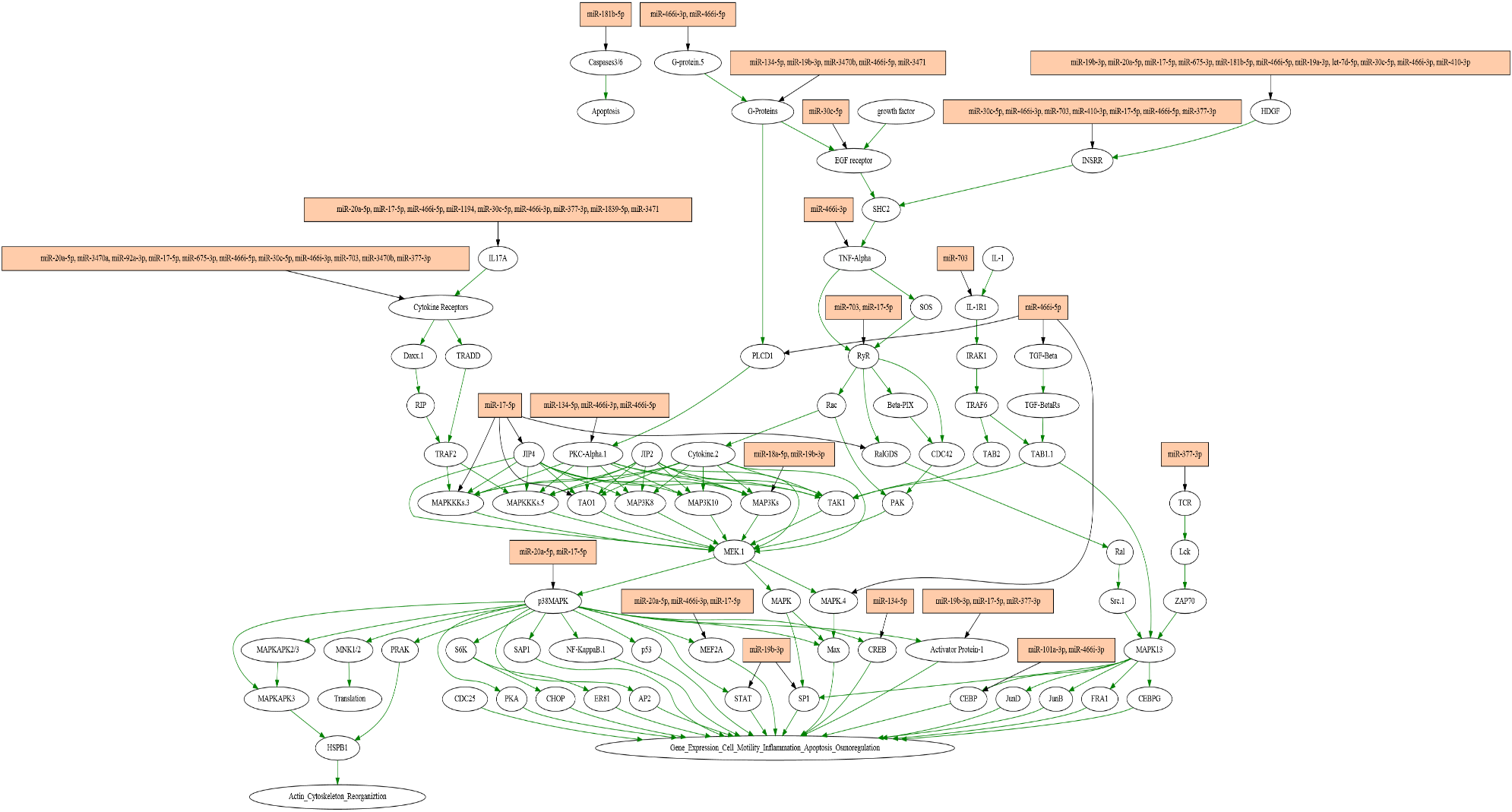


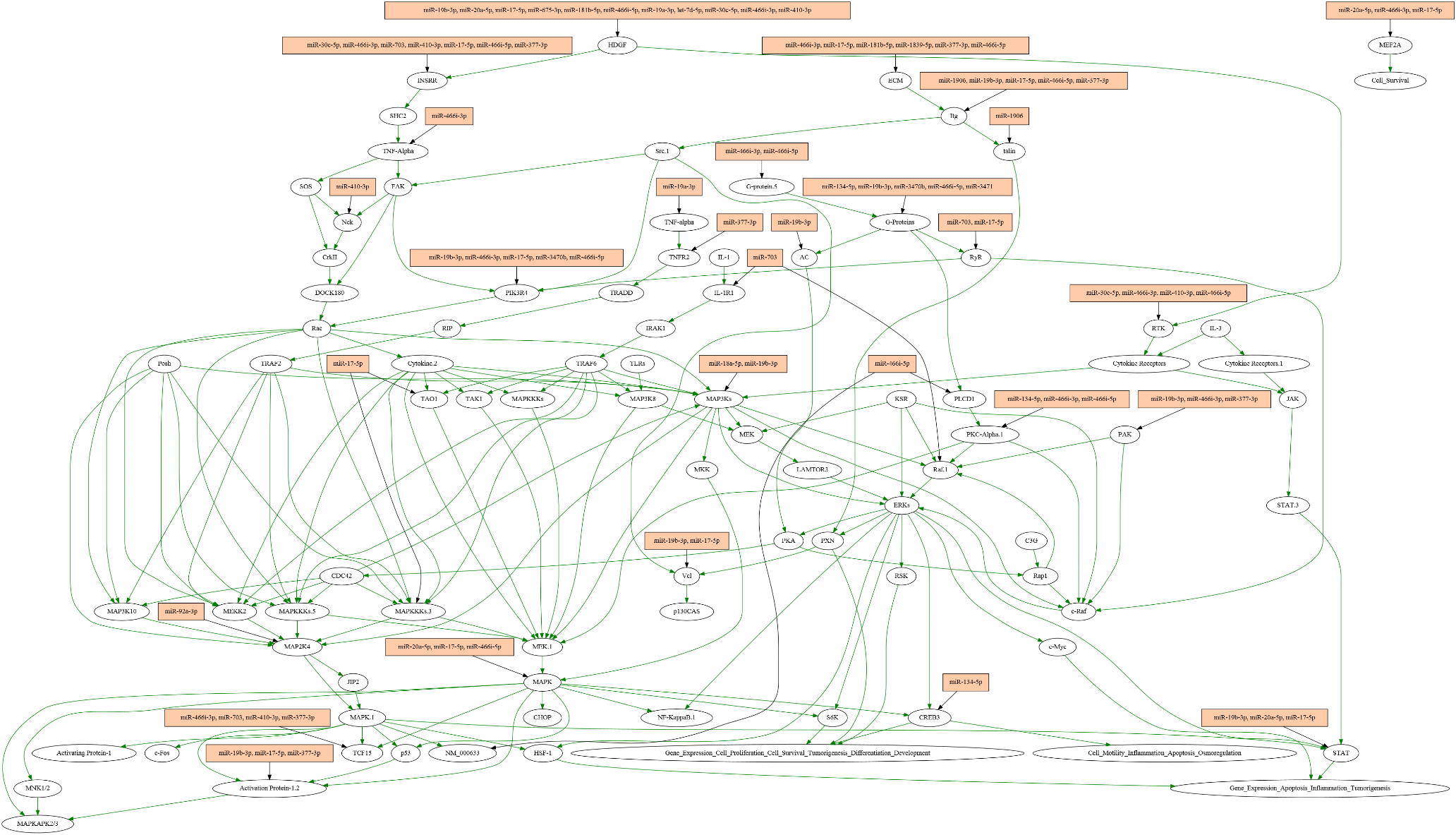
9) MAPK signaling pathway

10) MAPK family pathway


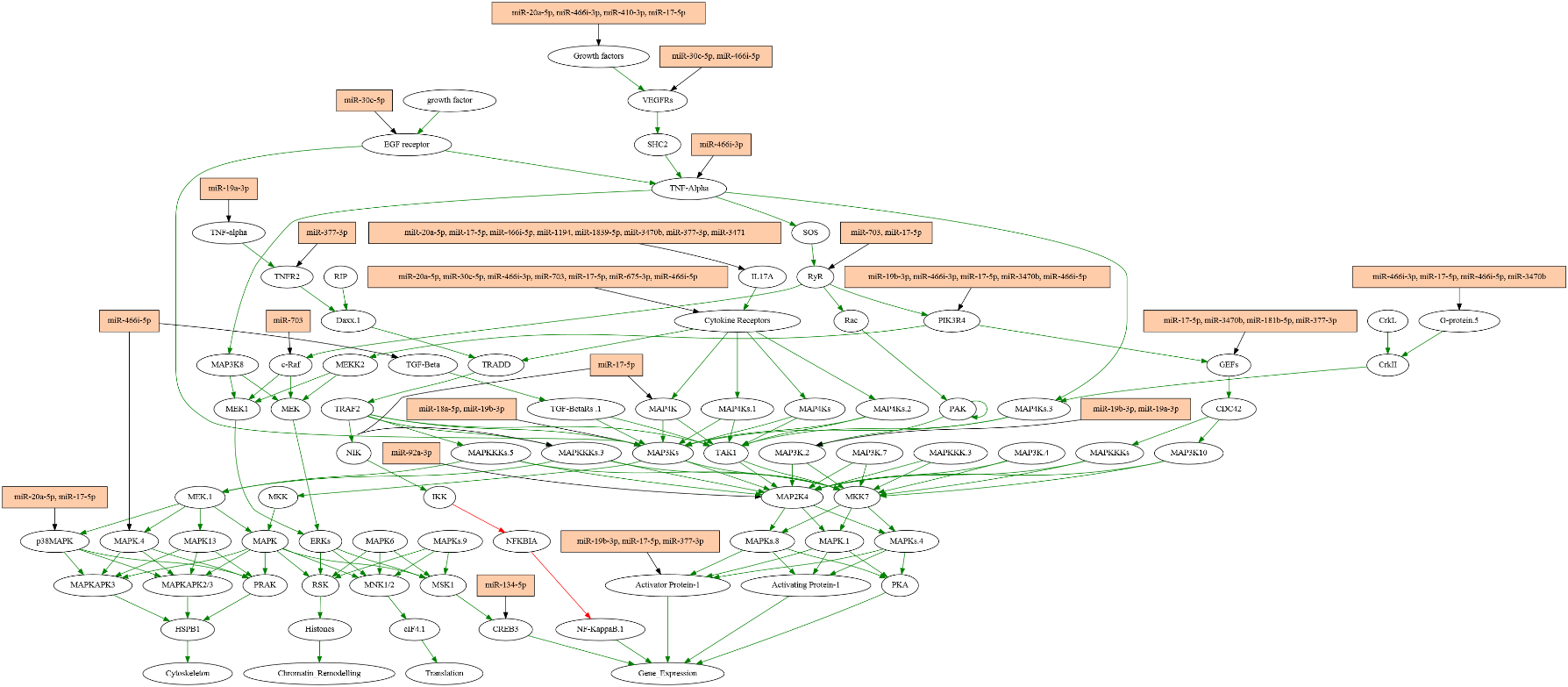


11) Jnk pathway


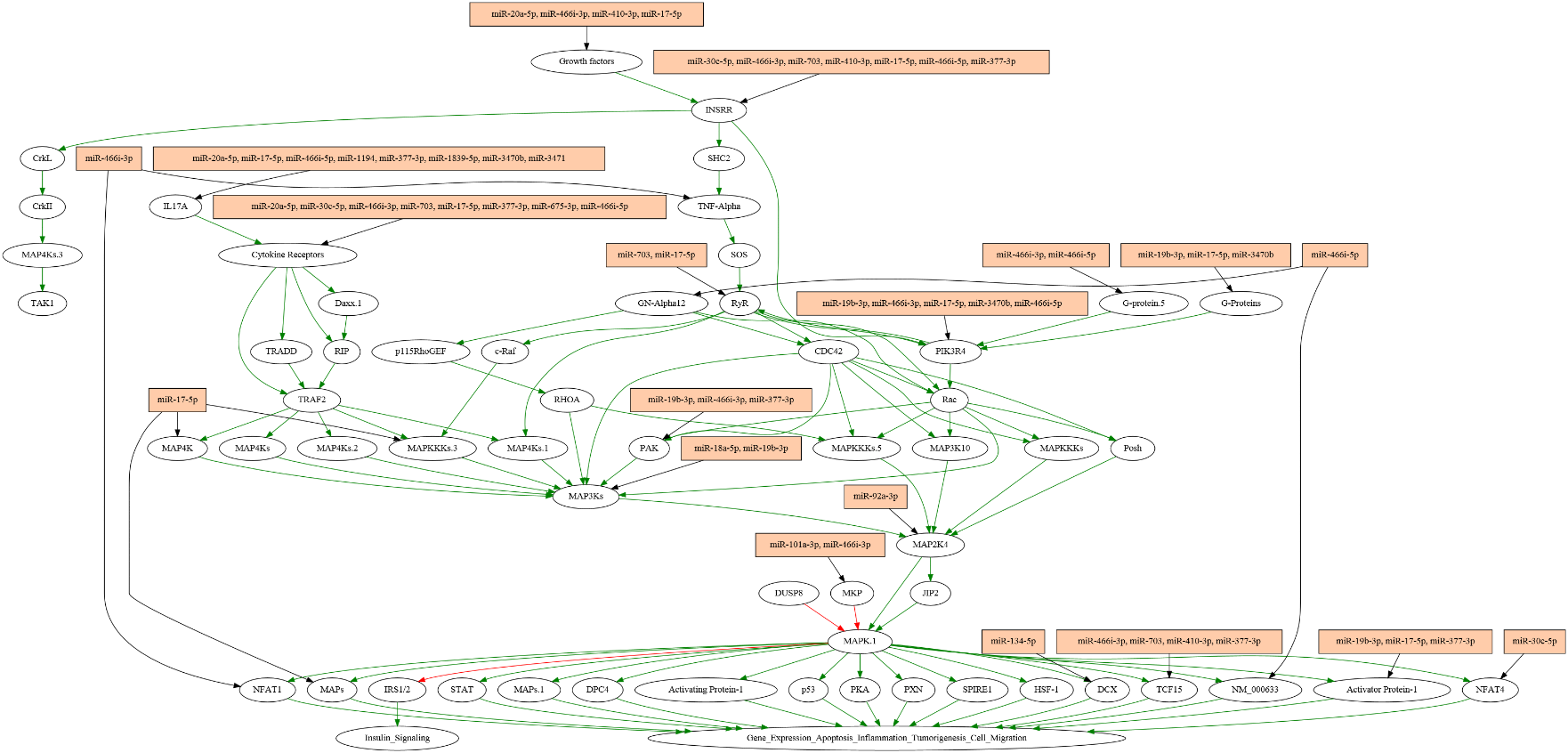


12) JAK-STAT pathway (Nml_SOCS_BCL-XL_p21_Myc_Nos2_Gene_Expression_via_STAT2)


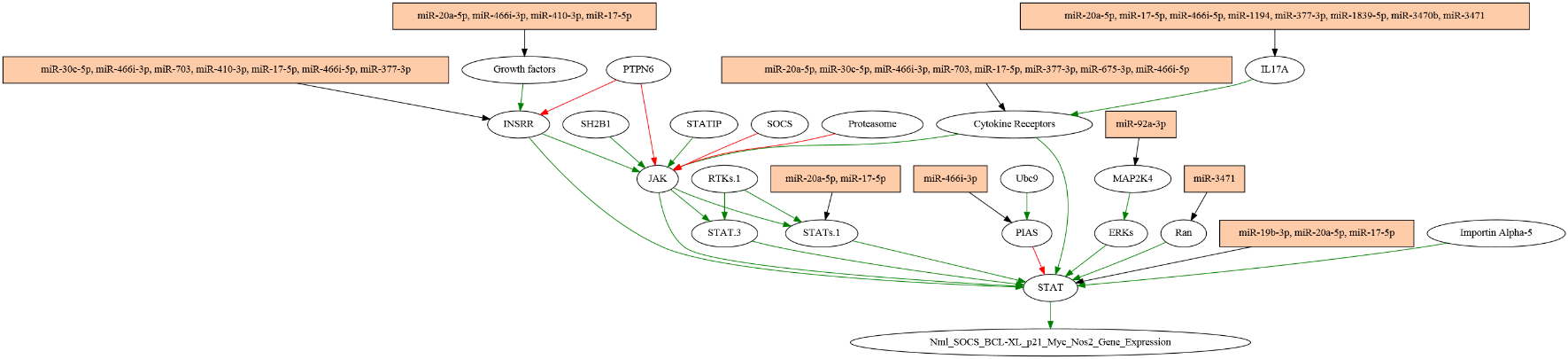


13) Jak-Stat pathway


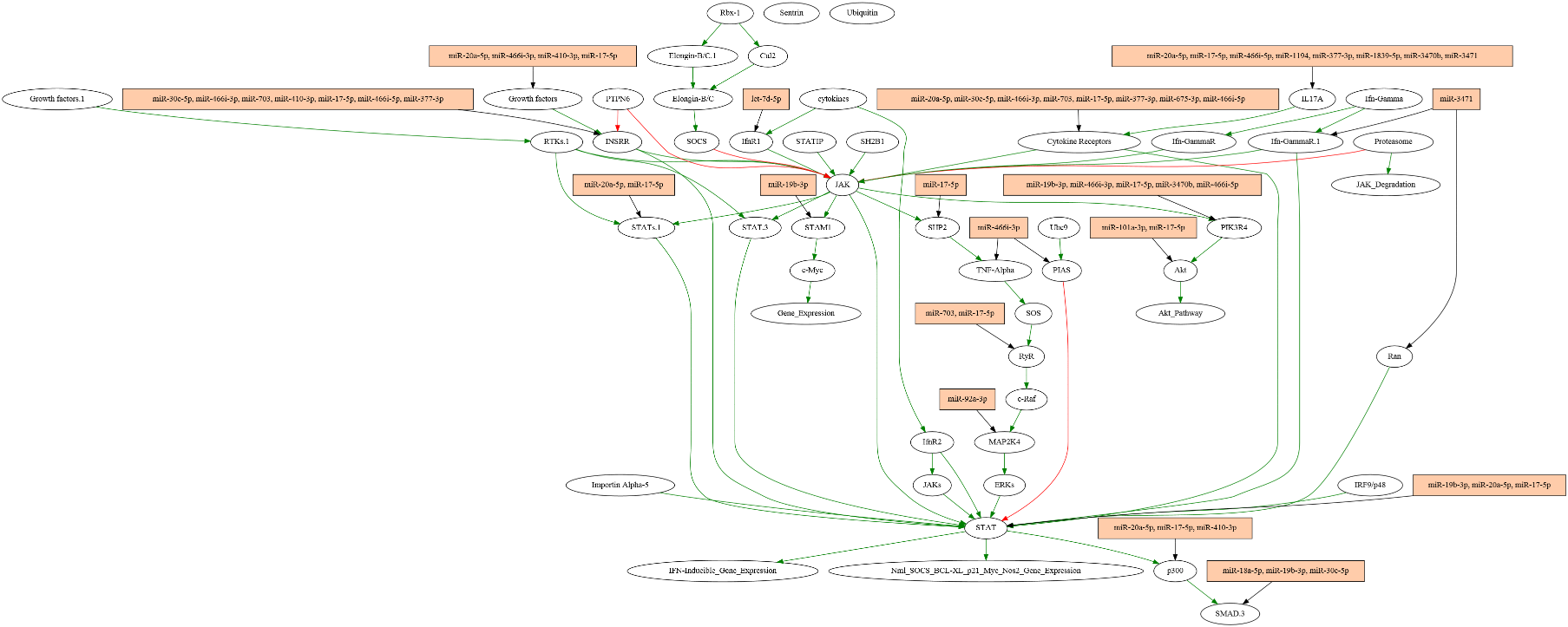


14) Estrogen pathway


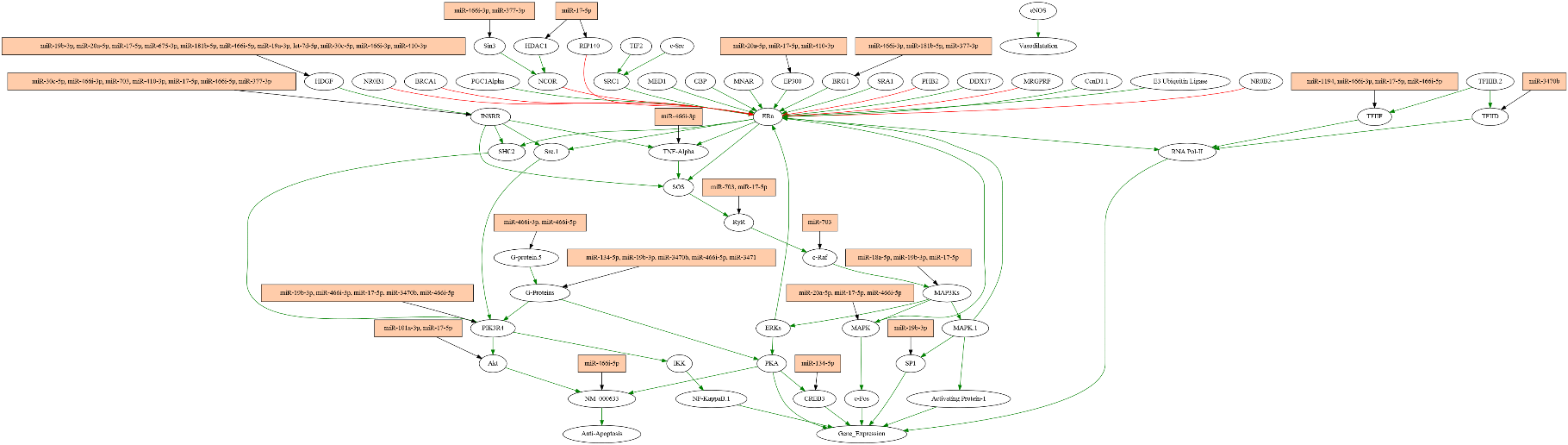


15) ERK pathway


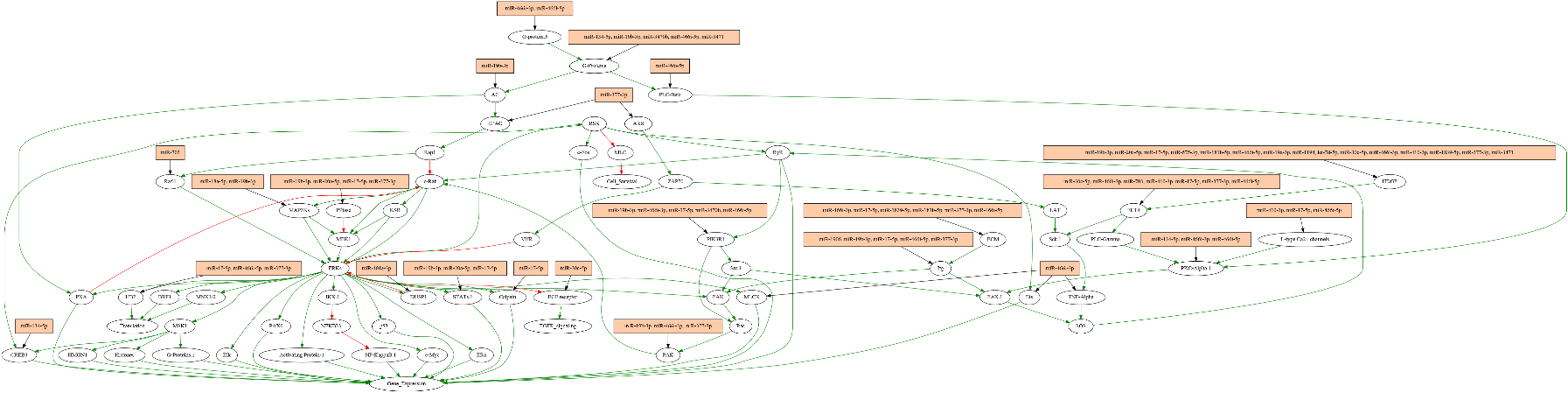

Supplement: Supplementary file 10 [file Data_Sheet_10.DOCX]
