# Supplementary material for: Exploration of the Transcriptional Landscape of ALPPS Reveals the Pathways of Accelerated Liver Regeneration
Source: Front Oncol. 2019 Nov 19;9:1206. doi: 10.3389/fonc.2019.01206 (PMC6882302; doi:10.3389/fonc.2019.01206)
Supplement: Supplementary file 11 [file Data_Sheet_11.DOCX]

Table: Patients Characteristics

| **Patient** | **Age** | **Gender** | **Weight** | **Indication** | **Co-morbidity** | **Histology** | **Increase in FLR volume*** | **Postoperative complications** |
| --- | --- | --- | --- | --- | --- | --- | --- | --- |
| 1 | 55 | F | 54 | synchronous liver metastases of adeno-carcinoma of the rectum | none | slight pericellular fibrosis,  no higher fibrosis,  no steatosis | 20.5% | wound dehiscence, pneumothory, ohylothorax, electrolyte disorder, renal insufficiency |
| 2 | 57 | M | 91 | synchronous liver metastases of adeno-carcinoma of the rectum | none | macro-steatosis  ca. 20%,  no fibrosis | 3.5% | VAC, antibiotics |
| 3 | 35 | F | 61 | synchronous liver metastases of adeno-carcinoma of the rectum | cancer of right breast | none | 29.3% | none |
| 4 | 37 | F | 65 | synchronous liver metastases of adeno-carcinoma of the sigmoid colon | none | minimal pericellular fibrosis without bridging | 18.2% | blood transfusion |
| 5 | 47 | M | 82 | metachronous liver metastases of adeno-carcinoma of the rectum | none | macro-steatosis ca. 30% | 22.5% | bed side wound opening, antipyretics |

*represents future liver remnant (FLR) volume before step 2 minus liver volume before step 1 of ALPPS
