# Supplementary material for: Exploration of the Transcriptional Landscape of ALPPS Reveals the Pathways of Accelerated Liver Regeneration
Source: Front Oncol. 2019 Nov 19;9:1206. doi: 10.3389/fonc.2019.01206 (PMC6882302; doi:10.3389/fonc.2019.01206)
Supplement: Supplementary file 12 [file Data_Sheet_12.DOCX]

**
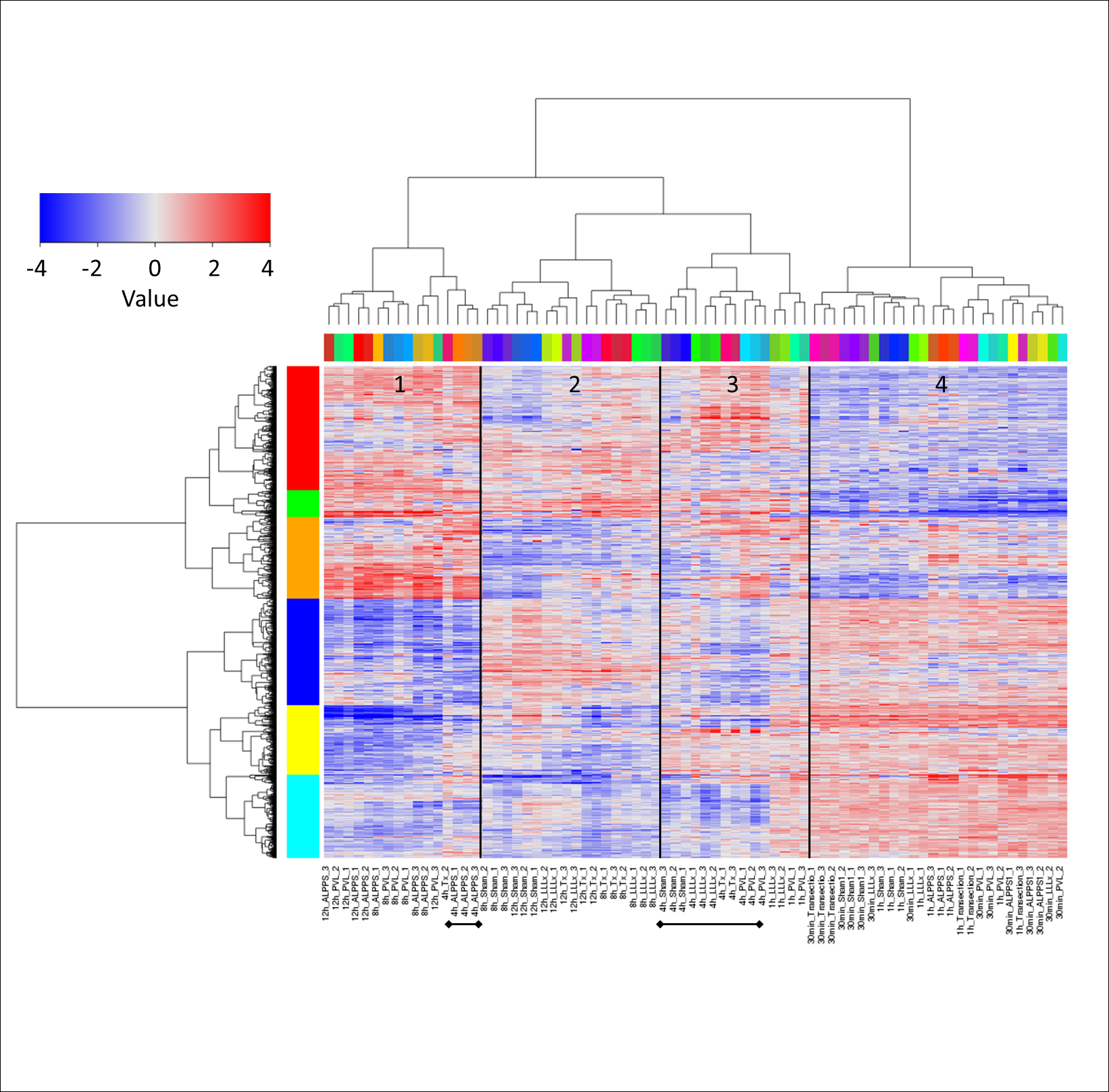
**

**Suppl. Data S-XII.** Hepatic gene expression profiles derived from RNA sequencing at 0.5, 1, 4, 8 and 12 h after surgery. Note that ALPPS samples isolated 4 h after surgery grouped together with all late samples of PVL and ALPPS (≥8 h post OP).
