# Supplementary material for: Exploration of the Transcriptional Landscape of ALPPS Reveals the Pathways of Accelerated Liver Regeneration
Source: Front Oncol. 2019 Nov 19;9:1206. doi: 10.3389/fonc.2019.01206 (PMC6882302; doi:10.3389/fonc.2019.01206)
Supplement: Supplementary file 12 [file Data_Sheet_9.DOCX]

Supplementary Data S-IX

Complete list of all affected intracellular signaling pathways, including pathway activation strength (PAS) values after ALPPS in humans.

Intracellular Signaling Pathway	10_1L_Norm	10_3L_FLR	12_1L_Norm	12_3L_FLR	13_1L_Norm	13_3L_FLR	15_1L_Norm	15_3L_FLR	16_1R_Norm	16_3R_FLR
AHR_Pathway	-0.160522872	0.024456813	0.027845751	0.024105282	0.027492848	0.145884532	0.082382948	0.025522678	0.022801306	0.318767708
AHR_Pathway_C-myc_expression_via_RELA	-0.210269453	-0.022568804	0.039940107	-0.025939218	0.05563677	0.150710483	0.086437501	-0.0094054	0.028255047	0.357850161
AHR_Pathway_Cath_D_expression_via_SP1	-0.199723527	-0.069278642	0.034396066	-0.035324138	0.067315209	0.132935821	0.075743026	-0.029694602	0.022269193	0.306102595
AHR_Pathway_CYP1A1_CYP1B1_CYP1A2_AHRR_gene_expression_via_POLR2B	-0.120224229	0.20314731	0.056725253	0.107628044	-0.022902789	0.179118985	0.085490358	0.100157325	0.000911388	0.344872122
AHR_Pathway_PS2_Gene_expression_via_ESR1	-0.21989321	-0.059662163	0.048534706	-0.034421303	0.0687351	0.150014842	0.082415828	-0.023392112	0.020207545	0.339141636
Akt_Signaling_Pathway	0.088604199	-0.127428275	0.011236939	0.120641654	-0.002000616	0.066029929	-0.10779793	-0.038413424	0.00995741	0.189039303
Akt_Signaling_Pathway_Acetylation_of_proteins	0.01580292	0.008007711	-0.100413218	0.054927755	-0.038843581	-0.017961257	0.062981221	0.064810704	0.06047265	0.131761618
Akt_Signaling_Pathway_Aggregation_and_Neurodegeneration	-0.01195984	0.016997615	-0.085308935	0.077314806	-0.030255463	0.059734093	0.078450626	0.113782775	0.049073612	0.160396261
Akt_Signaling_Pathway_Apoptosis	-0.019699988	0.001960979	-0.106389048	0.066774986	-0.018766827	0.043683289	0.065070406	0.098067563	0.079785449	0.182940834
Akt_Signaling_Pathway_AR_mediated_apoptosis	0.007753486	0.036576022	-0.098774569	0.078454235	-0.030249428	0.044382227	0.058826582	0.119955105	0.062443909	0.212325457
Akt_Signaling_Pathway_Blocks_Apoptosis	-0.004412085	0.011692859	-0.106342899	0.046595243	-0.028223632	0.023743499	0.069295868	0.099530118	0.069682734	0.155950589
Akt_Signaling_Pathway_Blood_cell_differentiation	0.004595885	-0.001499591	-0.102943627	0.045129179	-0.029446927	-0.000238519	0.061051639	0.081871194	0.066743019	0.135425829
Akt_Signaling_Pathway_Cell_Cycle	0.061956733	0.069360375	-0.187808424	-0.07220858	-0.065342344	0.175641771	0.020019127	0.082870355	0.171174882	-0.162133114
Akt_Signaling_Pathway_Cell_Cycle_Progression	0.026660338	-0.072853601	-0.083092815	0.029741325	-0.021506215	-0.103575871	0.049493097	0.028403176	0.02844558	-0.027752025
Akt_Signaling_Pathway_Cell_Survival	-0.068649722	0.099117756	-0.093260916	0.069626072	0.041406874	0.173118324	0.040866537	0.199908052	0.079637212	0.297522183
Akt_Signaling_Pathway_Elevation_of_Glucose_Import	0.031415944	-0.027025753	-0.137136275	-0.059206103	-0.036892335	-0.099602104	0.04685573	0.050785442	0.095756924	0.097809825
Akt_Signaling_Pathway_Enhancement_of_Breast_Epithelial	0.004595885	-0.001499591	-0.102943627	0.045129179	-0.029446927	-0.000238519	0.061051639	0.081871194	0.066743019	0.135425829
Akt_Signaling_Pathway_ERK_mediated_apoptosis	0.002365981	-0.007316334	-0.105931167	0.059907526	-0.030491621	0.015843827	0.06627188	0.087785826	0.067784908	0.13754939
Akt_Signaling_Pathway_Genetic_Stability	-0.007525395	-0.024576486	-0.098661443	0.049266424	-0.045865094	-0.025324607	0.066559149	0.072090686	0.085492776	0.13597408
Akt_Signaling_Pathway_Glucose_Uptake	0.117932782	0.514366354	-0.234429181	0.16692512	-0.214992928	-0.218171786	0.383316291	0.017625007	-0.05182695	0.47283984
Akt_Signaling_Pathway_Glycogen_Synthesis_and_Apoptosis	0.02455109	-0.032577388	-0.101537232	0.030792682	-0.032051362	-0.022314684	0.044050618	0.057377033	0.064986871	0.094720851
Akt_Signaling_Pathway_Increased_GLUT4_translocation	0.003933523	-0.028804002	-0.097325746	0.015405475	-0.026373504	-0.047705579	0.061998626	0.06552994	0.057767093	0.090053545
Akt_Signaling_Pathway_Induction_of_Chromatin_Condensation	-0.001653426	-0.014642494	-0.084063566	0.031035881	-0.030406785	-0.011120737	0.054144145	0.068353823	0.061979622	0.11540176
Akt_Signaling_Pathway_JNK_mediated_apoptosis	-0.032533769	-0.066554076	-0.090855635	0.082899273	-0.01812148	0.048285059	0.053022978	0.062228389	0.088487889	0.144597822
Akt_Signaling_Pathway_Neuroprotection	-0.005202817	0.039148034	-0.084048842	0.061315868	-0.02358323	0.068875872	0.05005992	0.106626552	0.062774962	0.226131675
Akt_Signaling_Pathway_NF-kB_dependent_transciption	0.012010051	0.034891502	-0.116138186	0.049244219	-0.025134439	0.035712532	0.060255911	0.102369061	0.069006652	0.202043192
Akt_Signaling_Pathway_NF-kB_pathway	0.004055291	0.066966417	-0.119467821	0.100699783	-0.033379408	0.096779276	0.087175723	0.163393811	0.061616206	0.249129311
Akt_Signaling_Pathway_NFAT_degradation	0.006926459	-0.131353068	-0.015529666	0.024501359	-0.02911441	-0.060655224	0.013798502	0.011380804	0.023919099	0.007000703
Akt_Signaling_Pathway_NO_production	0.000694985	0.035112215	-0.082408661	0.086878676	-0.047473532	0.046482757	0.053956719	0.11218815	0.075230477	0.187662628
Akt_Signaling_Pathway_p73_mediated_apoptosis	0.007480719	-0.0397988	-0.087772256	0.025480163	-0.019255482	-0.046467779	0.036550083	0.048854539	0.062996929	0.063253664
Akt_Signaling_Pathway_Promotes_Adipogenesis	-0.014503228	-0.009028472	-0.121621312	-0.031187734	-0.039073741	-0.033471976	0.086553863	0.047247283	0.088644409	0.057992131
Akt_Signaling_Pathway_Protein_Synthesis	-0.018520548	0.024354476	-0.072618418	0.019271293	-0.020246481	0.035715663	0.04117386	0.070330215	0.070211572	0.15117965
Akt_Signaling_Pathway_Proto-Oncogenic_and_RTK-signaling	-0.007944771	-0.002068901	-0.085471645	0.030559497	-0.068988682	-0.003582638	0.076930568	0.0792021	0.085474522	0.108438144
Akt_Signaling_Pathway_Regulation_of_Cyclic_Nucleotide	0.001004682	-0.00858152	-0.092814903	-0.001028983	-0.026655677	-0.034390024	0.050438677	0.0467533	0.068027204	0.086637464
Akt_Signaling_Pathway_Regulation_of_Na+_Transport	0.032414665	0.706996058	-0.125727194	0.390322935	-0.034474726	0.744790417	0.358644118	0.589761997	-0.230856897	0.884729346
Akt_Signaling_Pathway_Splicing_Regulation	-0.010346794	0.077694727	-0.127938241	0.203894936	-0.090921045	0.280795129	0.179697015	0.316293904	0.049509044	0.392360181
Akt_Signaling_Pathway_Survival_Genes	-0.037782914	0.05581173	-0.072540909	0.065340085	-0.021204242	0.075551084	0.078039738	0.127231751	0.053488321	0.213941505
Akt_Signaling_Pathway_Synaptic_Transmission	0.067230604	-0.014025866	-0.088343173	0.08925254	-0.039156218	0.030527816	0.022280368	0.071714009	0.037988406	0.142349902
Akt_Signaling_Pathway_Translation	-0.05249424	0.132749705	-0.036895887	0.011278166	-0.032194228	0.082870413	0.114771179	0.187003387	0.00681317	0.1922146
Akt_Signaling_Pathway_Tumor_Supression	-0.002387633	0.062157898	-0.074782591	0.09163918	-0.062385288	0.029815633	0.057431739	0.115722894	0.082123762	0.22270846
ATM_Pathway	0.043392269	0.122724638	-0.073886056	0.119941817	0.009578212	0.201004849	0.018680881	0.132123258	0.002234692	0.337284317
ATM_Pathway_Apoptosis	-0.021418547	-0.026785331	-0.011552297	-0.026231257	0.033835802	0.108561122	-0.047435101	0.014854881	0.046570131	0.040898665
ATM_Pathway_Apoptosis_and_Senescence	-0.114841166	-0.196891241	-0.030835383	0.176986988	0.123474453	0.25612267	0.004741179	0.146395015	0.017460871	0.209940022
ATM_Pathway_Cell_Cycle_Checkpoint_Control	-0.067291629	-0.185958311	0.04246296	-0.012297408	-0.048597062	0.006746662	0.055697248	-0.018945785	0.017728459	0.094953941

Supplementary Data S-IX

Complete list of all affected intracellular signaling pathways, including pathway activation strength (PAS) values after ALPPS in humans.

ATM_Pathway_Cell_Survival	0.041507958	0.186361017	-0.040078403	0.116666669	-0.021794294	0.235377066	-0.009431041	0.269406639	0.029795797	0.53163544
ATM_Pathway_Checkpoint_Activation	0.034816634	-0.048983525	-0.081004929	0.114598936	-0.023772383	0.054963013	0.084168374	0.156914486	-0.014207688	0.102127094
ATM_Pathway_DNA_repair	-0.050813155	-0.21409481	0.016039744	-0.036997824	0.030446443	0.063220781	0.117580518	0.087235308	-0.113253565	-0.006413059
ATM_Pathway_G2-Mitosis_progression	0.07336502	0.210529144	-0.213960892	0.290322081	0.046330084	0.327920661	0.039163919	0.109352339	0.055101848	0.67496648
ATM_Pathway_G2_M_Checkpoint_Arrest	0.07336502	0.210529144	-0.213960892	0.290322081	0.046330084	0.327920661	0.039163919	0.109352339	0.055101848	0.67496648
ATM_Pathway_NF-kB_Pathway	-0.070032556	-0.214207459	-0.103989881	-0.086875894	0.052824849	0.036977781	-0.008930833	0.074337879	0.130128394	-0.11780896
ATM_Pathway_Repair_and_Recombination	-0.021418547	-0.026785331	-0.011552297	-0.026231257	0.033835802	0.108561122	-0.047435101	0.014854881	0.046570131	0.040898665
ATM_Pathway_S-phase_arrest	0.073479477	-0.117510682	-0.109510906	0.024312448	-0.014895486	-0.047142702	-0.028823816	-0.129786441	0.079750735	-0.028899431
ATM_Pathway_S-phase_progression	0.151278642	0.081312262	-0.150764202	0.047119595	0.190700764	0.348738161	0.014885245	0.1741929	-0.206100469	-0.117547242
ATM_Pathway_Synaptic_Vesicle_Transport	-0.024709556	-0.136890654	-0.051354386	0.004575773	0.015110625	0.069366043	0.043042741	0.055344364	0.017910558	0.086242355
BRCA1_Pathway	-0.034359381	-0.154854306	-0.007381389	-0.044588622	0.041622359	-0.006026379	-0.012942523	-0.093284421	0.013060928	0.011872011
BRCA1_Pathway_Base_Excission_Repair	-0.090727441	-0.525919555	0.171906559	-0.142007723	0.135714159	-0.337727793	0.007528592	-0.0965579	-0.224421913	-0.507138256
BRCA1_Pathway_Cell_Cycle_Arrest_DNA_Repair_Genes_p21_WAF_CIP1_14-3-3_(	-0.163736753	-0.214459335	-0.149621295	0.095674196	0.341515957	0.469210327	0.052333383	0.342433901	-0.080491349	0.295320729
BRCA1_Pathway_Chromatin_Remodeling	-0.000558481	-0.182461146	-0.073073609	-0.063057049	0.030792211	-0.047817597	0.01344615	-0.048187645	0.029393716	-0.056009206
BRCA1_Pathway_E2_Responsive_Genes	-0.191306261	-0.492042772	-0.065221825	-0.091870853	0.229762613	-0.171944995	-0.041840333	-0.406294435	0.068605813	-0.409645756
BRCA1_Pathway_G1_S_arrest	-0.207637586	0.142906666	-0.252194446	0.140406491	0.302018696	0.758279949	-0.014660818	0.418740542	0.172474131	0.757335523
BRCA1_Pathway_Growth_Promoting_Genes_hTert_S100A7	0.03543248	0.261125743	-0.273187716	0.519819262	0.015399909	0.706930608	0.328763446	0.74396506	-0.106408127	0.905413726
BRCA1_Pathway_Homologous_Recombination_Repair	-0.072946687	-0.266263623	-0.020022178	-0.226626568	0.180026542	0.050637803	-0.079220568	-0.214731757	-0.00783713	0.102746604
BRCA1_Pathway_Mismatch_Repair	-0.105256559	-0.332533172	0.002461946	-0.095530723	0.15799659	0.173688817	0.034113124	0.115170304	-0.089315135	0.090629386
BRCA1_Pathway_NHEJ_DSB_Repair	-0.110772599	-0.193198058	-0.087103682	-0.030926879	0.241294952	0.321699343	-0.002438437	0.14295797	-0.040980268	0.180309412
BRCA1_Pathway_Nucleotide_Excission_Repair	0.05453271	-0.265647201	-0.294620963	-0.186148746	0.227305084	-0.109044346	0.011819526	-0.212805878	0.000963625	-0.266573248
BRCA1_Pathway_Transciption_Coupled_Repair	-0.114747824	-0.05042769	-0.197615754	0.151788639	0.302627737	0.365221806	0.020617904	0.219260619	-0.010882087	0.270311723
cAMP_Pathway	0.056278718	-0.10096258	0.012794442	0.08458832	-0.014306722	0.006296447	-0.051446886	-0.034095919	-0.003319554	0.101832061
cAMP_Pathway_Axonal_Growth	0.055451959	-0.074229699	0.073222908	0.008850967	0.0445609	0.050160814	-0.075197152	0.001072074	-0.098038627	0.101208461
cAMP_Pathway_Cell_Growth	0.02739037	-0.101654418	0.031562154	0.074839953	-0.064317166	-0.067893287	-0.004943538	-0.090421167	0.010308176	-0.017003285
cAMP_Pathway_Cell_Proliferation	-0.048833075	-0.161160732	-0.071774315	-0.109599138	0.000455984	0.004260143	0.140255851	0.250453553	-0.020104453	-0.062725198
cAMP_Pathway_Cell_Survival	0.026707681	-0.113362087	0.050843913	0.051140539	-0.029487015	-0.035656295	-0.029399574	-0.068631426	-0.018665011	0.01832503
cAMP_Pathway_Chemotaxis	0.029776897	-0.100432683	0.036453689	0.072651212	-0.062758877	-0.063055693	-0.010861616	-0.091674634	0.007389908	-0.005401839
cAMP_Pathway_Cytokine_Production	0.042382233	-0.091143556	0.022245841	0.074992959	-0.073017274	-0.071217814	-0.014922848	-0.077033274	0.023312048	0.012841747
cAMP_Pathway_Degradation_of_Cell_Cycle_Regulators	0.060240651	-0.087771656	-0.064034273	-0.01119795	-0.021468588	-0.106727677	0.048124815	0.067622174	-0.022862619	-0.138326963
cAMP_Pathway_Endothelial_Cell_Regulation	-0.044965745	-0.036386597	-0.029902771	0.006804671	-0.008468795	-0.085431656	0.050522601	-0.070159487	0.032814713	-0.066029516
cAMP_Pathway_eNOS_Signaling_Cardiovascular_Homeostasis	-0.060143222	-0.038240363	-0.036374788	0.012368063	-0.040257767	0.094590484	0.154227468	0.345273939	-0.017451699	0.070158689
cAMP_Pathway_Fatty_Acid_Metabolism	-0.04926693	-0.103730894	0.030215399	0.070528953	-0.048054326	-0.034952037	0.055722415	-0.03348968	0.011383436	0.064030874
cAMP_Pathway_Gene_Expression_via_NFKB2_CREBBP_ELK1	0.028772286	0.054610731	-0.034009882	-0.001823172	-0.023345507	-0.056854542	0.060398396	0.087027496	-0.031815288	0.07880769
cAMP_Pathway_Glycogen_Synthesis	-0.061158161	-0.076774979	-0.062447244	-0.00185968	0.002900591	0.104278426	0.148431743	0.330209683	-0.027726943	0.077961664
cAMP_Pathway_Glycolisis	-0.078328752	0.088380884	-0.034547257	0.130922458	-0.010860146	0.121822964	0.060751084	0.110866875	0.062985032	0.196948848
cAMP_Pathway_Lipolysis	-0.031487175	-0.197853858	-0.04173639	-0.08025034	-0.038409559	-0.017657455	0.102177543	0.25093729	0.009455572	-0.078644625
cAMP_Pathway_Metabolic_Energy	-0.008684355	-0.301002586	0.070446662	-0.060672007	-0.029109588	-0.082096546	-0.040299275	-0.148719231	0.00764654	-0.111118138
cAMP_Pathway_Myocardial_Contraction	0.040602401	-0.052273128	-0.012923586	0.075772106	-0.011932781	-0.020176435	-0.011869483	-0.007200501	-0.003876552	0.013476247
cAMP_Pathway_Oncogenesis	-0.020596745	-0.131528815	-0.067872997	-0.063872188	-0.01827273	-0.009660217	0.132036186	0.247235707	-0.025293726	-0.056640528
cAMP_Pathway_Protein_Retention	-0.101730772	-0.121075988	-0.065694128	-0.103611506	-0.016312205	0.087645512	0.191679898	0.302469995	-0.007942802	0.003793201
cAMP_Pathway_Regulation_of_Cytoskeleton	-0.052138628	0.078668027	-0.097030836	-0.069378007	0.003689461	0.122598498	0.182000055	0.337718093	-0.03652006	0.042877141
Caspase_Cascade_Pathway	-0.054695005	0.123683024	-0.026701698	-0.056927153	0.000203148	-0.026614392	0.080818363	0.059658304	0.000375187	-0.092309604
Caspase_Cascade_Pathway_Activated_Tissue_Trans-glutaminase	0.007719295	-0.080457227	0.011198164	0.041293895	0.011949557	-0.013507464	-0.016788668	-0.055045579	-0.014078351	-0.096185046
Caspase_Cascade_Pathway_DNA_Fragmentation	-0.082996004	0.274159731	-0.102147228	0.082548862	0.16320182	0.445229885	-0.009505095	0.178660037	0.031446523	0.314280137

Supplementary Data S-IX

Complete list of all affected intracellular signaling pathways, including pathway activation strength (PAS) values after ALPPS in humans.

CD40_Pathway	0.072353108	0.114742936	-0.059854367	0.034530749	-0.036904479	0.037081302	-0.031077154	0.03028881	0.055482909	CD40_Pathway
CD40_Pathway_Cell_Survival	0.038937289	-0.078595975	0.044744189	-0.290549695	0.034725239	-0.208662897	0.010918816	-0.31361635	-0.129325484	CD40_Pathway_
CD40_Pathway_Gene_Expression_Cell_Adhesion_Molecule_via_NFKB2	0.068661754	0.159023777	-0.095209711	0.049576889	-0.005517041	0.080554012	-0.011653497	0.128509896	0.043718512	CD40_Pathway_
CD40_Pathway_Gene_Expression_Cell_Survival_via_NFKB2	0.068661754	0.159023777	-0.095209711	0.049576889	-0.005517041	0.080554012	-0.011653497	0.128509896	0.043718512	CD40_Pathway_
CD40_Pathway_Gene_Expression_Co-Stimulatory_Molecules_via_NFKB2	0.068661754	0.159023777	-0.095209711	0.049576889	-0.005517041	0.080554012	-0.011653497	0.128509896	0.043718512	CD40_Pathway_
CD40_Pathway_Gene_Expression_COX2_and_Prostaglandins_via_NFKB2	0.068661754	0.159023777	-0.095209711	0.049576889	-0.005517041	0.080554012	-0.011653497	0.128509896	0.043718512	CD40_Pathway_
CD40_Pathway_Gene_Expression_Immunoglobulin_Class_Switch_via_NFKB2	0.068661754	0.159023777	-0.095209711	0.049576889	-0.005517041	0.080554012	-0.011653497	0.128509896	0.043718512	CD40_Pathway_
CD40_Pathway_Gene_Expression_Pro-Inflamatory_Cytokines_via_NFKB2	0.068661754	0.159023777	-0.095209711	0.049576889	-0.005517041	0.080554012	-0.011653497	0.128509896	0.043718512	CD40_Pathway_
CD40_Pathway_Gene_Expression_Procoagulant_Activity_via_NFKB2	0.068661754	0.159023777	-0.095209711	0.049576889	-0.005517041	0.080554012	-0.011653497	0.128509896	0.043718512	CD40_Pathway_
Cellular_Apoptosis_Pathway	-0.072310033	0.200222549	-0.031925048	-0.078213718	-0.002494393	0.007158274	0.107176256	0.103357771	-0.00044679	Cellular_Apopto
Cellular_Apoptosis_Pathway_Depolarization	-0.146520002	-0.247945493	0.115269711	0.24561652	0.205163114	0.262290955	-0.027201606	0.20756852	-0.146711266	Cellular_Apopto
Cellular_Apoptosis_Pathway_DNA_Fragmentation	-0.022515598	0.204077648	-0.140025698	-0.113229633	0.09442755	0.016667176	-0.005988249	-0.037032873	0.074102006	Cellular_Apopto
Cellular_Apoptosis_Pathway_Gene_Expression_BAX_BID_BAK_Ras_Noxa_PUMA_	-0.132533181	-0.231085946	0.080746375	0.198260459	0.188570346	0.264501393	-0.019939317	0.188730303	-0.116844275	Cellular_Apopto
Cellular_Apoptosis_Pathway_Gene_Expression_FLIP_CIAP2_BFL1_BCL2_via_NFK	0.079640267	0.164826407	-0.12387007	0.064552786	-0.006799261	0.103310132	0.025306259	0.175082414	0.025722821	Cellular_Apopto
Chemokine_Pathway	0.022047663	-0.073438858	0.034114493	0.047767731	-0.00265271	0.081990117	-0.045437822	-0.019606361	-0.008071626	Chemokine_Path
Chemokine_Pathway_Cell_Activation	-0.202815976	-0.011441571	0.059670074	0.14207253	-0.059657741	0.09915261	0.230538287	0.18910432	-0.027734643	Chemokine_Path
Chemokine_Pathway_Gene_Expression_and_Apoptosis_via_ELK1	-0.094216828	0.104998081	0.102217643	0.194928913	-0.109966742	0.151626602	0.169436775	0.191672308	-0.067470825	Chemokine_Path
Chemokine_Pathway_Internalization_Degradation_Recycling	-0.010047108	0.029872261	0.132214631	0.082184588	-0.06871161	0.047819693	-0.027151791	-0.100670947	-0.026304142	Chemokine_Path
Chromatin_Pathway	0.013156629	-0.195343066	-0.02260117	-0.105028681	-0.029099704	-0.062339571	0.000943809	-0.119453125	0.037600424	Chromatin_Path
Circadian_Pathway	-0.14380971	0.211902904	-0.294910815	0.170701115	0.044258717	0.368781581	0.165551016	0.122599453	0.228910798	Circadian_Pathw
CREB_Pathway	0.080929624	-0.184672025	0.031259159	0.079344307	0.01537994	0.006599032	-0.119198752	-0.132693796	-0.008369969	CREB_Pathway
Cytokine_Network_Pathway	0.00270477	0.033250781	0.016452303	0.038371883	0.039134567	0.111040758	-0.035895183	-0.013825879	-0.022396454	Cytokine_Netwo
EGF_Pathway	0.051586824	0.004081274	0.00642941	0.048371374	-0.029593936	0.065441206	-0.035044099	0.019227275	0.00662181	EGF_Pathway
EGF_Pathway_Actin_Cytoskeletal_Rearrangement	-0.007037815	0.030803433	0.030658233	0.052489278	-0.051230806	0.197127467	0.005574043	-0.027998308	0.022036376	EGF_Pathway_A
EGF_Pathway_Cell_Motility	0.046044204	0.09947288	-0.029407379	-0.219351338	0.125094096	-0.2044628	-0.11468072	-0.325258951	-0.027050206	EGF_Pathway_C
EGF_Pathway_Cell_Survival	-0.014495491	-0.118580209	0.025714172	-0.006330866	-0.043976035	-0.054553748	-0.001791095	0.020740949	0.034548439	EGF_Pathway_C
EGF_Pathway_Cytoskeleton_Regulation	0.011055452	-0.008001055	0.021411569	-0.111191879	0.060666021	-0.087315186	-0.092927001	-0.094258429	-0.000206014	EGF_Pathway_C
EGF_Pathway_EGFR_Endocytosis	0.034873165	0.227721941	-0.035294269	-0.16884727	0.109673214	-0.044016052	-0.11380681	-0.255742771	0.004554733	EGF_Pathway_E
EGF_Pathway_Gene_Expression_via_FOS_NFKB2_MYC_STAT1_ELK1_STAT3_JUN	0.149728932	0.135897711	-0.041595381	0.091155789	-0.000410186	0.193116792	-0.09497896	0.087134008	-0.012744393	EGF_Pathway_G
EGF_Pathway_IP3_Signaling	0.107013926	0.13576299	-0.069371327	-0.076253694	0.063097849	-0.199968975	-0.154691446	-0.289136013	0.053951	EGF_Pathway_IP
EGF_Pathway_Rab5_Regulation_Pathway	0.133352735	-0.459394236	0.037581578	-0.110821452	-0.178846429	-0.515043418	-0.016430769	-0.423773557	0.024342864	EGF_Pathway_R
ErbB_Family_Pathway	0.063117409	-0.082322744	-0.016996706	-0.062256011	0.001142707	-0.010128697	-0.042569713	0.012514816	-0.004693699	ErbB_Family_Pa
ErbB_Family_Pathway_Anti-Apoptosis	0.00440765	-0.018030178	-0.193100588	-0.123552889	0.12940832	-0.036194652	-0.133965099	0.181484879	0.193249678	ErbB_Family_Pa
ErbB_Family_Pathway_Gene_Expression_via_JUN_FOS_ELK1	0.168359965	-0.035898397	-0.002803366	0.00553769	-0.014449467	0.009843406	-0.180713173	-0.049108322	0.02960605	ErbB_Family_Pa
ErbB_Family_Pathway_Translation	-0.083743197	0.32064037	-0.177408762	0.149302947	-0.044671553	0.269663535	0.255382409	0.370615572	0.05044112	ErbB_Family_Pa
ERK_Signaling_Pathway	0.096216971	-0.158567372	0.045317875	0.117036145	0.000684314	0.028603345	-0.138310183	-0.077377869	-0.003908976	ERK_Signaling_P
ERK_Signaling_Pathway_Cell_Survival	-0.031994354	-0.205737927	0.07045074	-0.012745054	-0.004864349	-0.180209039	-0.040356894	-0.025401532	0.006764877	ERK_Signaling_P
ERK_Signaling_Pathway_EGFR_signaling	-0.017052301	-0.07820911	0.02303549	-0.017652044	0.014880429	-0.096392889	-0.037357706	-0.0655764	0.016494102	ERK_Signaling_P
ERK_Signaling_Pathway_Gene_Expression_via_CAPN6_TP53_FOS_ATF1_MYC_ELK	0.006091053	0.06685703	0.123277704	0.057410761	-0.049081357	0.078518379	-0.033468423	0.053082534	-0.046818975	ERK_Signaling_P
ERK_Signaling_Pathway_Translation	0.015537735	-7.63899E-05	-0.008334323	0.034692522	-0.008475304	-0.144938174	-0.030759463	-0.052036117	0.03203138	ERK_Signaling_P
Erythropoietin_Pathway	0.039326502	-0.068295167	0.008602283	-0.044976816	0.007411738	0.018890465	-0.07718516	-0.016645799	0.021844635	Erythropoietin_
Erythropoietin_Pathway_Anti-Apoptosis	-0.019452569	0.173032694	-0.079199184	0.065448483	0.003978688	0.207808857	0.049705131	0.26830464	0.044967938	Erythropoietin_
Erythropoietin_Pathway_BCLXL_Gene_Expression_via_STAT5B	0.254582678	-0.164051566	-0.09435795	-0.201183068	-0.019994741	-0.000471662	-0.166298948	-0.129994695	0.026068937	Erythropoietin_
Erythropoietin_Pathway_Cell_Cycle_Progression	-0.016922887	-0.049725364	0.017540549	-0.193752166	-0.029049402	-0.030270124	-0.094491678	-0.223746655	0.122923413	Erythropoietin_
Erythropoietin_Pathway_Cell_Survival	0.049378677	-0.02533851	0.03982696	-0.052839585	-0.01761601	0.024290237	-0.06764112	0.072469116	-0.00394851	Erythropoietin_

Supplementary Data S-IX

Complete list of all affected intracellular signaling pathways, including pathway activation strength (PAS) values after ALPPS in humans.

Erythropoietin_Pathway_Gene_Expression_Neuroprotection_via_NFKB2	0.212141222	0.349999585	-0.182350873	0.065037245	-0.058906686	0.194773021	-0.02178931	0.272520722	0.050905662	0.395771767
Erythropoietin_Pathway_GPI_Hidrolysis_and_Ca2+_influx	0.351448011	0.423137608	-0.275334818	0.168687545	-0.152370051	0.083773702	0.007238884	0.30533838	0.069017969	0.090072678
Estrogen_Pathway	0.060990984	-0.112329076	0.043905067	0.102498694	0.00235306	0.065018807	-0.098534588	-0.036514928	-0.008714521	0.168567988
Estrogen_Pathway_Anti-Apoptosis	0.014297375	-0.210518429	0.022284662	-0.082336477	0.004646921	-0.043198609	-0.004671326	-0.102336029	-0.036557637	-0.063016037
Estrogen_Pathway_Gene_Expression_via_FOS_JUN_ELK1_SP1_POLR2B_CREB3_NI	0.01105256	0.054371689	0.013153506	0.047799754	-0.006250861	0.116359467	-0.000458286	0.088087998	-0.017496919	0.238714045
Estrogen_Pathway_Vasodilatation	-0.070216194	0.659012496	0.369629383	0.751490951	-0.324478894	0.840982974	-0.127708554	0.545705199	0.152774259	0.940262377
FLT3_Signaling_Pathway	-0.027969382	-0.057585339	-0.014217307	-0.016166113	-0.015570926	0.026229891	0.023990585	0.019121533	0.033767033	0.106397886
FLT3_Signaling_Pathway_Transcription_via_ELK3_MAPK12_CREB3_STAT2	-0.049198916	-0.017666948	-0.0061642	-0.049795211	-0.010304162	0.043420144	0.025573433	0.041951798	0.040093851	0.182262023
FLT3_Signaling_Pathway_Translation	-0.014675449	-0.164657407	-0.022984263	-0.116421674	-0.012292285	-0.163702446	-0.01705936	0.015755695	0.067011394	-0.23686824
Glucocorticoid_Receptor_Signaling_Pathway	-0.045515553	0.007590379	0.000970093	-0.082415713	-0.006781725	-0.093692156	0.064406237	-0.044315755	-0.013079056	-0.317520508
Glucocorticoid_Receptor_Signaling_Pathway_Cell_Cycle_Arrest	0.035304434	-0.830948725	0.188524023	-1.350042671	0.220637143	-0.88602981	-0.555727094	-0.713791013	0.111261522	-1.019287094
Glucocorticoid_Receptor_Signaling_Pathway_Cell_Cycle_Progression	0.006492595	-0.205932219	-0.041660055	-0.294587072	0.021439251	-0.110785617	-0.006876877	-0.154023345	0.020605065	-0.472181334
Glucocorticoid_Receptor_Signaling_Pathway_Gene_Expression_via_CREB3_STAT	-0.014680912	-0.177235847	-0.03067184	-0.25326695	0.016763337	-0.065664939	-0.005308075	-0.118575002	0.033897483	-0.332951875
Glucocorticoid_Receptor_Signaling_Pathway_Histone_Deacetylation	-0.062248738	-0.279098235	0.028000329	-0.52509566	-0.008902561	-0.454694331	-0.029348786	-0.359013796	0.072499734	-0.581290349
Glucocorticoid_Receptor_Signaling_Pathway_Inflammatory_Cytokines	-0.065127361	-0.150999458	0.056498966	-0.182182011	0.039696897	-0.095753649	0.004648066	-0.150073435	-0.035716578	-0.553340042
GPCR_Pathway	0.05862814	-0.10322766	0.020152092	0.050418962	-0.011457413	-0.004983587	-0.079477927	-0.063806768	0.012155109	0.116857957
GPCR_Pathway_Gene_Expression_via_JUN_NFKB2_ELK1_SRF_FOS_CREB3	0.055132244	0.111409743	0.010119792	0.064480078	-0.02515488	0.042796063	-0.020164149	0.038650094	-0.019933002	0.269554958
Growth_Hormone_Signaling_Pathway	0.02556977	-0.171495222	0.003901223	-0.087882189	0.017736372	-0.094446158	-0.039812713	-0.081444929	-0.007394649	-0.092052218
Growth_Hormone_Signaling_Pathway_Cell_Survival	0.056015021	-0.28274124	0.038275156	-0.192735055	-0.039986241	-0.177704884	-0.016775417	-0.141247425	-0.037528531	-0.243134508
Growth_Hormone_Signaling_Pathway_Gene_Expression_via_SRF_ELK1_STAT5B_	0.009316442	-0.186323153	0.017092885	-0.072671548	0.031008768	-0.070586681	-0.041895303	-0.079200671	-0.015522788	-0.0841255
Growth_Hormone_Signaling_Pathway_Glucose_Uptake	0.055742048	-0.372837926	0.015455758	-0.369089567	-0.023053529	-0.3052906	-0.042123092	-0.193766401	-0.006021196	-0.303855622
Growth_Hormone_Signaling_Pathway_Protein_Synthesis	0.192669577	-0.061351147	-0.112461316	-0.271148823	-0.00466999	-0.044931365	-0.008874041	-0.27538222	-0.066664231	-0.227869654
GSK3_Signaling_Pathway	-0.098138819	0.126347736	-0.047454828	-0.107208009	0.011279162	0.015704391	0.137510898	0.096624695	-0.003196414	-0.050777087
GSK3_Signaling_Pathway_b-CTNN_Degradation	-0.29460572	-0.055327009	0.081924424	-0.104826275	0.056510997	0.318764121	0.21234623	0.142204655	-0.056175926	0.305816562
GSK3_Signaling_Pathway_Gene_Expression_via_CTNNB1	-0.099735241	0.008068965	-0.004428756	-0.062262957	0.030715505	0.112632028	0.103865089	0.064267061	-0.030416597	0.131926165
GSK3_Signaling_Pathway_Glycogen_Synthesis	0.018812864	-0.04358991	-0.015801995	-0.080883372	0.064830295	0.047283186	-0.083774259	-0.05180672	0.015933089	-0.012376369
GSK3_Signaling_Pathway_Protein_Synthesis	0.038650374	-0.081815299	-0.003174881	-0.156205142	0.051012566	-0.058096205	-0.090193217	-0.13089219	0.003705157	-0.137946123
HA_syntesis_pathway	0.139403933	-0.038939611	0.056347326	-0.043384217	0.00731652	0.058642562	-0.2136629	-0.047263546	0.010595121	0.312910203
Hedgehog_Signaling_in_Mammals_no_Tubulin_Pathway	0.023276687	-0.088653583	0.03989374	-0.042808553	-0.014041729	-0.023703469	-0.05868234	-0.089702094	0.009553643	-0.032129202
Hedgehog_Signaling_in_Mammals_Pathway	0.04429051	-0.184187266	0.026901401	-0.052549565	-0.00212545	-0.181836615	-0.061402644	-0.201197638	-0.007663818	-0.172486869
HGF_Pathway	0.046605566	-0.069109577	0.05305388	0.051012477	-0.011559302	0.009753194	-0.038495017	0.018866635	-0.04960514	0.098351864
HGF_Pathway_Anoikis	0.090203653	-0.107081186	0.050399354	0.050722044	-0.050445961	-0.016158679	-0.050704827	0.052405419	-0.039452219	0.186137458
HGF_Pathway_Cell_Adhesion_and_Cell_Migration	0.092626562	-0.058821514	0.065785112	0.18037309	-0.012090881	0.019897642	-0.061045056	0.052550505	-0.08527575	0.05705436
HGF_Pathway_Cell_Cycle_Progression	0.214723237	0.157046183	0.06626592	0.225722467	-0.063681206	0.183329454	-0.13410722	0.176390753	-0.083200699	0.691709618
HGF_Pathway_Cell_Scattering	-0.014345918	-0.277714534	0.06010952	-0.091876556	-0.042066075	-0.160889982	0.027847479	-0.042937319	-0.031545027	-0.175009701
HGF_Pathway_Cell_Survival	-0.014345918	-0.277714534	0.06010952	-0.091876556	-0.042066075	-0.160889982	0.027847479	-0.042937319	-0.031545027	-0.175009701
HGF_Pathway_Gene_Expression_via_JUN_STAT3_FOS_ELK1_ETS2	0.162272671	0.166946491	0.095241899	0.150019775	-0.048305347	0.12680919	-0.122204568	0.090277792	-0.08700464	0.519264639
HGF_Pathway_IP3_Pathway	0.013878562	-0.22602777	0.181106435	-0.11617614	0.046792455	-0.272148274	-0.229437761	-0.47075555	-0.012339722	-0.181097056
HGF_Pathway_PKC_Pathway	0.075808986	-0.384764437	0.079486967	-0.119513372	0.053888299	-0.365054578	-0.153955333	-0.465446116	-0.055228954	-0.30976344
HGF_Pathway_Regulation_of_Cytoskeleton_Cell_Polarity_and_Cell_Motility	-0.132039497	-0.084885427	0.024070371	-0.14573889	0.063143944	0.139160954	0.071776456	0.069740692	-0.026951309	0.120086473
HIF1Alpha_Pathway	0.037267379	0.021349346	0.047536452	-0.098996237	0.004937176	0.020510728	-0.07863531	7.70896E-05	-0.011105692	-0.181712623
HIF1Alpha_Pathway_Gene_Expression_via_JUN_CREB3	0.013184026	0.155605351	0.09358468	0.064968839	-0.017480217	0.111914693	-0.042265048	0.076671087	-0.047023429	0.174869127
HIF1Alpha_Pathway_HIF1a_Degradation	0.265202552	-0.119093478	-0.251914114	-0.088802464	0.051182199	-0.16745472	-0.097910136	-0.091857255	0.033439483	-0.342459172
HIF1Alpha_Pathway_NOS_Pathway	0.013194708	0.063331309	0.108521733	0.000550171	-0.032070517	-0.005368585	-0.059591848	-0.050597765	-0.030054065	-0.051261535
HIF1Alpha_Pathway_p53_Hypoxia_Pathway	0.041158683	-0.059836489	0.049746127	-0.167002648	-0.000860213	-0.069898478	-0.095680063	-0.094385718	0.005635468	-0.385116558

Supplementary Data S-IX

Complete list of all affected intracellular signaling pathways, including pathway activation strength (PAS) values after ALPPS in humans.

HIF1Alpha_Pathway_VEGF_Pathway	0.013194708	0.063331309	0.108521733	0.000550171	-0.032070517	-0.005368585	-0.059591848	-0.050597765	-0.030054065	HIF1Alpha_Pathw
Hypoxia_induced_EMT_in_cancer_and_fibrosis_3_copy	0.086084977	-0.142891924	0.191442038	0.32026232	-0.047948878	0.223836499	-0.236649999	0.027886921	0.007071894	Hypoxia_induce
IGF1R_Signaling_Pathway	0.056683432	0.0574462	0.001206552	0.105068639	-0.01945079	0.09159558	-0.024992951	0.092355498	-0.013446244	IGF1R_Signaling_
IGF1R_Signaling_Pathway_Apoptosis	-0.087220372	-0.117473655	0.002903316	-0.095700078	0.123153093	0.049343787	-0.067975571	-0.244880095	0.029139543	IGF1R_Signaling_
IGF1R_Signaling_Pathway_Cell_Migration	0.111945851	-0.06504087	0.033232027	0.216348151	-0.01701346	0.084781974	-0.057729131	0.080332413	-0.070435292	IGF1R_Signaling_
IGF1R_Signaling_Pathway_Cell_Proliferation_Diferentiation_and_Apoptosis	0.021606446	-0.052332586	-0.006602294	-0.109450569	-0.018338006	0.002691671	-0.067384451	0.000817399	0.07071829	IGF1R_Signaling_
IGF1R_Signaling_Pathway_Cell_Survival	-0.071042877	0.106761353	-0.063063773	0.043967426	0.060645855	0.245006405	0.022089344	0.266184463	0.05137143	IGF1R_Signaling_
IGF1R_Signaling_Pathway_Gene_Expression_Proliferation_Growth_Survival_via_	-0.026652006	0.008021453	0.061358542	-0.043946107	0.081364299	-0.00832283	-0.103884986	-0.118326676	-0.012185853	IGF1R_Signaling_
IGF1R_Signaling_Pathway_Glucose_Uptake	0.209301118	-0.243459279	-0.183103032	-0.547744632	-0.152403464	-1.091629614	-0.052756682	-0.214589509	0.178962027	IGF1R_Signaling_
IGF1R_Signaling_Pathway_Glycogen_Synthesis	0.054974526	-0.081149932	-0.076947397	0.010549319	-0.0644246	-0.109866371	0.045460442	0.008013796	0.040937005	IGF1R_Signaling_
IGF1R_Signaling_Pathway_Protein_Synthesis	-0.035996601	0.117371017	-0.081350707	0.186122107	0.001041926	0.262664174	0.069466242	0.206551671	0.046839141	IGF1R_Signaling_
IL-10_Pathway	0.03455809	-0.088101358	-0.014316345	-0.078558398	-0.041725825	-0.054925104	-0.018595094	-0.02075206	0.040079183	IL-10_Pathway
IL-10_Pathway_IL-10_Responsive_Genes_Transcription_of_BCLXL_Cyclin-D1_D2_I	0.024412169	-0.352109124	-0.01486887	-0.17812591	-0.015893975	-0.205390214	-0.049898443	-0.13167575	0.056249125	IL-10_Pathway_I
IL-10_Pathway_Inflammatory_Cytokine_Genes_Expression_via_STAT3	0.024412169	-0.352109124	-0.01486887	-0.17812591	-0.015893975	-0.205390214	-0.049898443	-0.13167575	0.056249125	IL-10_Pathway_I
IL-10_Pathway_Stability_Determination	0.008567902	0.611091177	0.024490522	0.322414716	-0.306310014	0.498061473	0.246087037	0.578346347	0.027164577	IL-10_Pathway_S
IL-10_Pathway_Translational_Modulation	0.064549343	-0.076356845	-0.101704116	-0.155682988	-0.055997743	-0.038243844	-0.082569372	0.032569478	0.175721915	IL-10_Pathway_T
IL-2_Pathway	0.080610063	0.040202212	-0.034257049	0.072077236	0.036924501	0.143836614	-0.12248163	0.040873573	0.039204112	IL-2_Pathway
IL-2_Pathway_Actin_Reorganization	-0.093851176	-0.130883852	-0.076870668	0.00440029	0.128042604	0.690004824	-0.106998933	0.187184077	0.149678177	IL-2_Pathway_A
IL-2_Pathway_Apoptosis	0.089553781	0.419949976	-0.142724126	0.263536299	-0.164533233	-0.109988352	0.408514827	0.270585964	-0.190811276	IL-2_Pathway_Ap
IL-2_Pathway_Apoptosis_Inhibition	-0.012062054	-0.265006495	0.034832093	-0.042376933	0.043478812	0.020720901	-0.01906766	0.010518035	-0.047181208	IL-2_Pathway_Ap
IL-2_Pathway_Gene_Expression_via_FYN_LYN_RPS6KB1_PTK2B	-0.015891001	-0.047423534	-0.168445381	-0.092356497	0.012673238	0.122051073	0.062883392	0.128047251	0.108779769	IL-2_Pathway_G
IL-2_Pathway_IL2_Gene_Expression_via_POLR1E_EGR1_HMGA1_ELF1_POU2F1_M	0.210505206	0.541174108	-0.085322973	0.308857853	-0.080337352	0.376812382	-0.042550531	0.53201572	-0.002294347	IL-2_Pathway_IL
IL-2_Pathway_Protein_Synthesis	-0.035181717	-0.070368558	-0.304439228	-0.128694956	0.059276767	0.214085818	0.099359864	0.235201275	0.180984342	IL-2_Pathway_Pr
IL-6_Pathway	-0.031299836	-0.047489647	-0.039003947	-0.011101676	0.01160409	-0.025352173	0.020976795	-0.024723071	0.037722893	IL-6_Pathway
ILK_Signaling_Pathway	0.125258028	-0.115419666	0.027522783	0.155883634	-0.012264261	0.025905917	-0.134823842	-0.03308248	-0.005692708	ILK_Signaling_Pa
ILK_Signaling_Pathway_Actin_Polymerization_Cytoskeletal_Reorganization	-0.067159646	-0.085697382	0.054058383	0.014167039	0.030875423	0.079700338	-0.046991308	-0.042195121	0.029217147	ILK_Signaling_Pa
ILK_Signaling_Pathway_Apoptosis	-0.035198355	0.036161888	-0.127633575	0.199966244	-0.007727761	0.126681014	0.110291344	0.181159268	0.060268331	ILK_Signaling_Pa
ILK_Signaling_Pathway_Cell_Adhesion	-0.267603479	-0.313324324	0.335145969	0.316454828	-0.025845965	-0.35289225	0.020072166	-0.888782352	-0.061768709	ILK_Signaling_Pa
ILK_Signaling_Pathway_Cell_Cycle_Proliferation	0.065234068	-0.116619756	0.062353222	0.130091359	-0.019215445	-0.002235907	-0.033053133	0.040968386	-0.075318722	ILK_Signaling_Pa
ILK_Signaling_Pathway_Cell_Migration_Retraction	0.065817937	-0.111059715	0.067753574	0.131293066	-0.027858871	-0.000885533	-0.030653345	0.047922015	-0.075059307	ILK_Signaling_Pa
ILK_Signaling_Pathway_Cell_Motility	-0.090214509	-0.112333398	0.090731181	0.051890641	0.025297857	0.013086274	-0.04833351	-0.180628377	0.022518979	ILK_Signaling_Pa
ILK_Signaling_Pathway_Cytoskeletal_Adhesion_Complexes	0.222457641	-0.237980316	0.028224157	0.310394022	-0.045294781	0.026090233	-0.219827552	-0.061207131	0.014440536	ILK_Signaling_Pa
ILK_Signaling_Pathway_Epithelial_Mesenchymal_Transition	-0.114848092	0.124569739	-0.023622196	-0.180170973	-0.010252737	-0.045974008	0.182511416	-0.014230594	-0.033788395	ILK_Signaling_Pa
ILK_Signaling_Pathway_Epithelial_Mesenchymal_Transition_Tubulo-Interstitial_	0.199021487	-0.230572168	0.031765783	0.2546774	-0.051505829	-0.011988916	-0.188266701	-0.051360129	0.008985261	ILK_Signaling_Pa
ILK_Signaling_Pathway_G2_Phase_Arrest	0.065234068	-0.116619756	0.062353222	0.130091359	-0.019215445	-0.002235907	-0.033053133	0.040968386	-0.075318722	ILK_Signaling_Pa
ILK_Signaling_Pathway_Induced_Cell_Proliferation	-0.139864256	0.028481642	0.107000921	-0.155876651	0.140623959	-0.040431478	-0.11755755	-0.101043036	0.009796915	ILK_Signaling_Pa
ILK_Signaling_Pathway_Loss_of_Occludin_Barrier_Dysfunction	0.062559884	-0.030092984	0.071882163	0.208050032	-0.011128418	0.045973266	-0.059705923	0.033693419	-0.063607709	ILK_Signaling_Pa
ILK_Signaling_Pathway_Migration_Vasculogenesis	0.199021487	-0.230572168	0.031765783	0.2546774	-0.051505829	-0.011988916	-0.188266701	-0.051360129	0.008985261	ILK_Signaling_Pa
ILK_Signaling_Pathway_MMP2_MMP9_Gene_Expression_Tissue_Invasion_via_FC	0.358127735	0.753673491	-0.075672346	0.408261071	-0.063372094	0.478677449	-0.139759656	0.71212316	-0.079323651	ILK_Signaling_Pa
ILK_Signaling_Pathway_Opsonization	-0.200877856	-0.240186279	0.266760617	0.232963932	-0.001474461	-0.306661233	-0.054776081	-0.845108008	-0.009632225	ILK_Signaling_Pa
ILK_Signaling_Pathway_Regulation_of_Intermediate_Filaments	0	0	0	0	0	0	0	0	0	ILK_Signaling_Pa
ILK_Signaling_Pathway_Regulation_of_Junction_Assembly_at_Desmosomes	-0.267603479	-0.313324324	0.335145969	0.316454828	-0.025845965	-0.35289225	0.020072166	-0.888782352	-0.061768709	ILK_Signaling_Pa
ILK_Signaling_Pathway_Tissue_Morphogenesis	0.358127735	0.753673491	-0.075672346	0.408261071	-0.063372094	0.478677449	-0.139759656	0.71212316	-0.079323651	ILK_Signaling_Pa
ILK_Signaling_Pathway_Tumor_Angiogenesis	0.128801014	0.067322128	0.240385622	0.302063052	-0.214795678	-0.053487226	-0.236057	0.124319501	0.081666101	ILK_Signaling_Pa
ILK_Signaling_Pathway_Wound_Healing	-0.267603479	-0.313324324	0.335145969	0.316454828	-0.025845965	-0.35289225	0.020072166	-0.888782352	-0.061768709	ILK_Signaling_Pa

Supplementary Data S-IX

Complete list of all affected intracellular signaling pathways, including pathway activation strength (PAS) values after ALPPS in humans.

Interferon_Pathway	-0.017366396	-0.056929249	0.101474094	0.000796704	-0.058059235	-0.038469509	-0.031269815	-0.049868301	0.005221353	0.066776048
Interferon_Pathway_Gene_Expression_via_HIST1H3B_CREB3	-0.057679476	0.015464904	0.342579896	-0.030212715	-0.115921373	-0.044062499	-0.058319407	-0.160824482	-0.110659647	0.054463473
Interferon_Pathway_Transciption	-0.052075823	-0.088840443	0.015531359	-0.002168068	-0.028438241	0.074134081	0.050462427	0.085714878	0.01452028	0.107351301
Interferon_Pathway_Translation	-0.141639168	-0.010724068	0.214851743	0.054649129	-0.040261837	0.239028172	0.002401772	0.174735516	-0.035352535	0.46200431
IP3_Pathway	0.081802734	-0.122779091	0.014982712	0.035522033	0.021260914	-0.022043343	-0.114514325	-0.17840328	-0.003532036	0.002026223
IP3_Pathway_Gene_Expression_via_CREB3_NFATC2_MEF2D	-0.039958002	-0.018357413	0.072660343	0.004897419	0.062774659	0.002461916	-0.064535351	-0.075632997	-0.030941646	0.006371663
JAK-STAT_Pathway	0.075929303	-0.105446113	-0.02851397	0.014656625	0.021140173	-0.002952128	-0.084721511	-0.048338386	0.016166004	0.098351252
JAK-STAT_Pathway_Akt_Pathway	0.012242065	-0.224232946	0.026748188	-0.051704132	-0.043040154	-0.051850525	0.01652193	-0.0067864	-0.012472036	-0.008523911
JAK-STAT_Pathway_Gene_Expression_via_MYC	0.196129527	0.516273837	-0.072358761	0.422572187	-0.286943351	0.623967789	0.222979096	0.524162926	-0.059806446	1.225381762
JAK-STAT_Pathway_IFN-Inducible_Gene_Expression_via_STAT2_STAT1	-0.033250984	0.04815731	-0.06304326	-0.047469667	-0.000942456	0.055167329	0.075230402	0.042627481	0.022006297	0.073656099
JAK-STAT_Pathway_JAK_Degradation	0.29460572	0.055327009	-0.081924424	0.104826275	-0.056510997	-0.318764121	-0.21234623	-0.142204655	0.056175926	-0.305816562
JAK-STAT_Pathway_Nml_SOCS_BCL-XL_p21_Myc_Nos2_Gene_Expression_via_ST.	0.088942192	-0.158208759	-0.0299193	0.010024199	0.039620425	-0.032960917	-0.123406599	-0.105178473	0.024763281	0.053092002
JNK_Pathway	0.039688098	-0.124598038	0.013064243	0.028549216	0.009485768	0.007468807	-0.076136721	-0.058302243	0.013898611	0.097969255
JNK_Pathway_Gene_Expression_Apoptosis_Inflammation_Tumorigenesis_Cell_I	0.026076779	-0.004356055	0.038949304	0.023946374	-0.026439819	-0.091288617	-0.051440532	-0.069054262	0.012854269	-0.009418235
JNK_Pathway_Insulin_Signaling	-0.055961591	-0.173360907	0.054251433	-0.041665247	-0.001834783	-0.204069928	-0.019403503	-0.278231736	0.022948452	-0.408540397
MAPK_Family_Pathway	0.011511382	-0.037881102	0.043871735	0.022902227	-0.007182056	0.060725934	-0.047695381	-0.019214108	-0.00050568	0.168665789
MAPK_Family_Pathway_Chromatin_Remodelling	-0.171785478	-0.02800655	0.474899702	0.03674803	-0.152388223	0.004834639	0.075153048	-0.145263253	-0.225879049	0.024799069
MAPK_Family_Pathway_Cytoskeleton	0.098515152	0.240123544	-0.072789918	0.182710146	-0.126108723	0.098492662	-0.004917632	0.186463098	0.105301125	0.548892722
MAPK_Family_Pathway_Gene_Expression_via_ATF2_JUN_ELK1_NFKB2_CREB3	-0.029482852	0.229087995	0.048162322	0.185557149	-0.038977275	0.209933727	0.096478498	0.248649419	-0.076180682	0.449861308
MAPK_Family_Pathway_Translation	-0.303960064	0.306843193	0.210131873	0.442527602	-0.170678897	0.357100895	0.454869816	0.483551153	-0.190362714	0.486353204
MAPK_Signaling_Pathway	0.106596844	-0.122452954	0.018818675	0.143430386	-0.015950391	0.035716443	-0.108774607	-0.033434117	-0.000690516	0.176377632
MAPK_Signaling_Pathway_Cell_Motility_Inflammation_Apoptosis_Osmoregulati	0.029232653	0.006046935	-0.037549583	-0.052343623	-0.004259455	0.0134325	-0.037491634	0.015937182	0.050068039	0.212182876
MAPK_Signaling_Pathway_Cell_Survival	0.05770265	-0.287351409	0.195795323	0.066299795	-0.064546957	-0.185357359	-0.101293833	-0.172468781	-0.087657182	-0.057227268
MAPK_Signaling_Pathway_Gene_Expression_Apoptosis_Inflammation_Tumorige	0.106610529	0.135147954	-0.094388459	0.07784424	-0.038810413	0.06552578	0.059257042	0.059641287	-0.032668677	0.295417058
MAPK_Signaling_Pathway_Gene_Expression_Cell_Proliferation_Cell_Survival_Tu	0.063493234	-0.034738594	0.034136573	0.108477499	-0.015467276	0.009442734	-0.052859023	0.059738014	-0.029303506	0.116809093
Mismatch_Repair_in_Eukaryotes_Pathway	-0.033226701	-0.217402035	0.021181195	-0.172813609	0.1063777	0.033171955	-0.052730034	-0.239301017	-0.04160217	-0.084098071
Mitochondrial_Apoptosis_Pathway	-0.003604849	0.038700648	0.016212674	-0.038028177	-0.012305204	-0.071364408	0.008518061	-0.051214416	-0.008820687	-0.010183882
Mitochondrial_Apoptosis_Pathway_Apoptosis	-0.100387251	0.013911841	0.034151836	-0.123495007	0.03294799	-0.02235955	0.065484486	-0.053327588	-0.032197057	0.083678464
Mitochondrial_Apoptosis_Pathway_Depolarization	-0.020699402	0.195267133	-0.050355051	0.167828206	0.008658878	0.193441237	0.114833867	0.226475665	-0.052438332	0.309478817
Mitochondrial_Apoptosis_Pathway_DNA_Fragmentation	-0.078319111	0.143654518	0.019959063	-0.167102104	0.069789334	-0.140921733	0.037139837	-0.113363814	-0.048569092	-0.245450835
Mitochondrial_Apoptosis_Pathway_Gene_Expression_via_TP53	-0.024149302	0.227811656	-0.05874756	0.195799574	0.010102025	0.225681443	0.133972844	0.26422161	-0.061178054	0.36105862
mTOR_Pathway	0.063180268	-0.087998319	0.056068672	0.094917942	-0.016482155	0.024860718	-0.085072176	-0.040945453	-0.017694605	0.163298891
mTOR_Pathway_Actin_Organization	0.082445589	-0.194423498	0.062623954	0.101293008	-0.023933026	0.026002965	-0.118040888	-0.040758763	-0.003095623	0.227700422
mTOR_Pathway_Autophagy	-0.142947908	-0.066282766	-0.00353081	0.158963595	0.011918795	0.542948321	0.027653091	0.412626564	0.106906829	0.481595088
mTOR_Pathway_Cap-dependent_Translation	-0.139477673	0.111638374	0.078502571	0.115605325	-0.014824646	0.345463637	0.0503177	0.253531556	0.02548205	0.524212444
mTOR_Pathway_Inflammation_Stress_Resistance	0.125852365	0.138143279	-0.089660022	-0.011416006	-0.005415857	-0.02611734	-0.051194182	0.084262038	0.020417706	0.299175999
mTOR_Pathway_Lipid_Synthesis	0.094164651	-0.144953892	0.062594534	0.140550524	-0.041270635	0.026873018	-0.091055062	-0.009434115	-0.024433478	0.224106961
mTOR_Pathway_Metabolism_Stress_Response_and_Apoptosis	-0.024627041	0.490179715	-0.047472011	0.418726824	-0.138065949	0.308085974	0.463013211	0.261128976	-0.252848216	0.456237286
mTOR_Pathway_Microtubule_Organization	0.097726496	-0.15814951	0.05943776	0.13548689	-0.035521423	0.050446849	-0.107073644	-0.010209624	-0.014569181	0.253836223
mTOR_Pathway_Mitochondria_Proliferation_and_Function	0.06601155	0.127836563	-0.148140842	-0.004021695	-0.093514275	-0.492823146	0.29135231	0.083242824	-0.1157087	0.130012725
mTOR_Pathway_mRNA_Biogenesis	-0.149705368	0.171515467	-0.061978344	0.125850998	0.021367852	0.323365043	0.185089387	0.377385398	0.005226477	0.379208093
mTOR_Pathway_Ribosome_Biogenesis	-0.129589982	0.098288581	0.147487275	0.060978865	-0.019240944	0.241180605	-0.002295196	0.153659821	0.003638845	0.429274013
mTOR_Pathway_Translation_Elongation	-0.109493093	0.229886929	0.004692127	0.282863002	-0.00357463	0.415223538	0.081666926	0.359542566	0.02670867	0.527706238
mTOR_Pathway_VEGF_Pathway	-0.04810695	0.323974808	-0.20725739	0.157231783	-0.024771787	0.525693635	0.172166179	0.463846723	0.107969958	0.871559987
NGF_Pathway	-0.013684069	-0.005248889	0.009303665	-0.003014025	-0.036080696	-0.015747722	0.055236991	0.040526827	-0.014775892	0.089523374

Supplementary Data S-IX

Complete list of all affected intracellular signaling pathways, including pathway activation strength (PAS) values after ALPPS in humans.

NGF_Pathway_Actin_Polymerization_Neurite_Outgrowth_and_Differentiation	-0.084192991	-0.122634258	0.021660132	-0.052223656	0.046002824	0.053361813	-0.018462706	-0.037962105	0.034992745	0.1575014
NGF_Pathway_Apoptosis	0.05456539	0.031280593	-0.10858257	0.052392853	-0.02777652	0.065954311	0.1322344	0.105021395	-0.050440724	0.128130994
NGF_Pathway_Gene_Expression_via_MYC_ELK1_CREB3_NFKB2	0.032130401	0.258631956	-0.007569638	0.148346925	-0.081330888	0.140420922	0.083476768	0.161853493	-0.026706626	0.480061234
NGF_Pathway_Neurite_Outgrowth_and_Differentiation	0.007593185	-0.224902567	0.035608061	-0.085352097	-0.043752893	-0.232699152	0.013699388	-0.026568029	-0.01314775	-0.261931933
NGF_Pathway_Neuronal_Survival	0.006286898	-0.278163366	0.047203301	-0.100065169	-0.05191374	-0.240201312	0.019126317	-0.073043392	-0.02070279	-0.260776299
Notch_Signaling_Pathway	-0.00392043	-0.055990166	0.115169655	0.077764717	-0.034860797	0.043191186	-0.057081192	-0.020396878	-0.019307252	0.090123937
Notch_Signaling_Pathway_gamma_Secretase	-0.164010055	-0.150408395	-0.079272181	-0.250979823	0.059230211	-0.064447526	0.212305751	-0.038910599	-0.028253722	-0.037886175
Notch_Signaling_Pathway_Gene_Expression_Chromatin_Remodeling_via_RBPJ	0.031655043	-0.035008338	0.158378952	0.150819059	-0.055769909	0.067110899	-0.116944957	-0.016282717	-0.017319147	0.118570628
Notch_Signaling_Pathway_Transcription_of_Target_Genes_Hairy_E-Spl_via_RBPJ	0.031655043	-0.035008338	0.158378952	0.150819059	-0.055769909	0.067110899	-0.116944957	-0.016282717	-0.017319147	0.118570628
p38_Signaling_Pathway	0.074193131	-0.10077584	0.018147063	0.082597032	0.011440426	0.060740216	-0.11159729	-0.055912383	0.007816675	0.201534558
p38_Signaling_Pathway_Actin_Cytoskeleton_Reorganiztion	-0.009411438	0.375979064	-0.036073272	0.205042706	-0.047034953	0.244897727	0.074816398	0.1942088	0.017703253	0.66693265
p38_Signaling_Pathway_Apoptosis	0	0	0	0	0	0	0	0	0	0
p38_Signaling_Pathway_Gene_Expression_Cell_Motility_Inflammation_Apoptos	0.118694225	0.205261135	-0.049335992	0.041165996	-0.034525027	0.043814786	-0.064452513	0.157048097	0.029619315	0.42295513
p38_Signaling_Pathway_Translation	-0.001448817	0.405223984	-0.027188934	0.132649465	-0.109496149	-0.101521175	-0.001825405	0.175454924	0.139959341	0.329331115
p53_Signaling_Pathway	0.049032845	0.098758301	-0.009648518	0.054647714	0.005084361	0.076252581	-0.037608391	0.10092708	-0.006860311	0.09676166
p53_Signaling_Pathway_Apoptosis	0.046338361	0.04599811	0.004749023	0.039184467	0.034125591	0.079243341	-0.07957299	0.0091876	-0.005640002	-0.036176444
p53_Signaling_Pathway_Breast_Cancer	0.050401422	0.035910011	0.001955252	0.02022056	0.039377662	0.071237897	-0.085896519	0.002529774	-0.005837835	-0.067430536
p53_Signaling_Pathway_Cancer	0.046338361	0.04599811	0.004749023	0.039184467	0.034125591	0.079243341	-0.07957299	0.0091876	-0.005640002	-0.036176444
p53_Signaling_Pathway_Cell_Cycle_Arrest	0.050401422	0.035910011	0.001955252	0.02022056	0.039377662	0.071237897	-0.085896519	0.002529774	-0.005837835	-0.067430536
p53_Signaling_Pathway_Cell_Growth_Accumulation	0.050401422	0.035910011	0.001955252	0.02022056	0.039377662	0.071237897	-0.085896519	0.002529774	-0.005837835	-0.067430536
p53_Signaling_Pathway_DNA_Repair	0.050401422	0.035910011	0.001955252	0.02022056	0.039377662	0.071237897	-0.085896519	0.002529774	-0.005837835	-0.067430536
p53_Signaling_Pathway_Exosome_Mediated_Secretion	0.050401422	0.035910011	0.001955252	0.02022056	0.039377662	0.071237897	-0.085896519	0.002529774	-0.005837835	-0.067430536
p53_Signaling_Pathway_Gene_Expression_Anti-Apoptosis_via_TP53	0.046338361	0.04599811	0.004749023	0.039184467	0.034125591	0.079243341	-0.07957299	0.0091876	-0.005640002	-0.036176444
p53_Signaling_Pathway_Gene_Expression_Cell_Cycle_and_Cell_Growth_via_TP5	0.046338361	0.04599811	0.004749023	0.039184467	0.034125591	0.079243341	-0.07957299	0.0091876	-0.005640002	-0.036176444
p53_Signaling_Pathway_Gene_Expression_Cell_Fate_and_Development_via_TP5	0.046338361	0.04599811	0.004749023	0.039184467	0.034125591	0.079243341	-0.07957299	0.0091876	-0.005640002	-0.036176444
p53_Signaling_Pathway_Gene_Expression_Cell_Signaling_via_TP53	0.046338361	0.04599811	0.004749023	0.039184467	0.034125591	0.079243341	-0.07957299	0.0091876	-0.005640002	-0.036176444
p53_Signaling_Pathway_Gene_Expression_DNA_Replication_and_Repair_via_TP5	0.046338361	0.04599811	0.004749023	0.039184467	0.034125591	0.079243341	-0.07957299	0.0091876	-0.005640002	-0.036176444
p53_Signaling_Pathway_Gene_Expression_ECM_and_Adhesion_via_TP53	0.046338361	0.04599811	0.004749023	0.039184467	0.034125591	0.079243341	-0.07957299	0.0091876	-0.005640002	-0.036176444
p53_Signaling_Pathway_Gene_Expression_Infection_and_Immune_Response_vi	0.046338361	0.04599811	0.004749023	0.039184467	0.034125591	0.079243341	-0.07957299	0.0091876	-0.005640002	-0.036176444
p53_Signaling_Pathway_Inhibition_of_Angiogenesis_and_Metastasis	0.050401422	0.035910011	0.001955252	0.02022056	0.039377662	0.071237897	-0.085896519	0.002529774	-0.005837835	-0.067430536
p53_Signaling_Pathway_Inhibition_of_IGF1R_mTOR_Pathways	-0.219652712	0.104607441	0.115666606	0.39182058	0.00884685	0.626131415	0.244015008	0.690381527	-0.148875833	0.819065571
p53_Signaling_Pathway_Normal_Cell_Cycle_Progression	0.046338361	0.04599811	0.004749023	0.039184467	0.034125591	0.079243341	-0.07957299	0.0091876	-0.005640002	-0.036176444
PAK_Pathway	0.062764234	-0.138228278	0.03363058	0.076554383	0.018230634	0.08215033	-0.112846827	-0.058939986	-0.001778622	0.142207405
PAK_Pathway_Actin_Organization	0.060688274	-0.150402614	0.056637581	0.069561547	0.025344498	0.083517571	-0.130743666	-0.081828124	-0.011926685	0.139823249
PAK_Pathway_Cell_Survival	0.060688274	-0.150402614	0.056637581	0.069561547	0.025344498	0.083517571	-0.130743666	-0.081828124	-0.011926685	0.139823249
PAK_Pathway_Contractility_Stress_Fibres_and_Focal_Adhesion	0.065128368	-0.015011788	-0.043188545	0.082004919	-0.001912582	-0.006709261	-0.030211567	-0.005464853	0.010184323	-0.022723304
PAK_Pathway_Lamelliopodia_and_Filopodia_Outgrowth	0.054735353	-0.137941247	0.053359099	0.077780255	0.022670175	0.104763871	-0.1227212	-0.058594261	-0.008043426	0.160534539
PAK_Pathway_Paxillin_Disassembly	0.051239196	-0.05036635	0.140145704	-0.075853169	-0.095723361	-0.454627454	-0.130218506	-0.187378258	0.034556989	-0.050301351
PAK_Pathway_Transciptional_Activation_and_Filopodia_Formation	0.102820847	-0.127070544	0.142949112	-0.02566695	-0.020685673	-0.132081993	-0.185739312	-0.200349879	-0.039344977	-0.036626557
PPAR_Pathway	0.074813387	-0.033361173	0.009559073	0.059382522	-0.011807863	-0.047851341	-0.027398907	-0.005191963	-0.045165685	0.00605932
PPAR_Pathway_Adipocyte_Differentiation_Glucose_Homeostasis_and_Macroph	0.146600955	-0.031501948	-0.030810765	-0.133116638	-0.114415191	-0.564316432	0.096703824	-0.241605629	-0.09807878	-0.104795413
PPAR_Pathway_Fatty_Acid_Metabolism_Lipid_Homeostasis_and_Skin_Proliferat	0.146600955	-0.031501948	-0.030810765	-0.133116638	-0.114415191	-0.564316432	0.096703824	-0.241605629	-0.09807878	-0.104795413
PPAR_Pathway_Peroxisome_Proliferation_Hepatocarcinogenesis_Fatty_Acid_Me	0.146600955	-0.031501948	-0.030810765	-0.133116638	-0.114415191	-0.564316432	0.096703824	-0.241605629	-0.09807878	-0.104795413
PTEN_Pathway	0.031344549	0.046814196	-0.024330918	0.012046134	-0.020373339	-0.041497044	-0.007006661	0.011861515	0.020366369	-0.051944839
PTEN_Pathway_Adhesion_or_Migration	0.033023103	0.072288078	-0.040632283	0.000840082	-0.027282581	-0.044549584	0.008068478	0.024970118	0.02682328	-0.054355458

Supplementary Data S-IX

Complete list of all affected intracellular signaling pathways, including pathway activation strength (PAS) values after ALPPS in humans.

PTEN_Pathway_Angiogenesis_and_Tumorigenesis	0.175597977	-0.082960355	-0.026580928	0.163111736	-0.033347749	-0.052251756	-0.162025848	-0.06592253	0.04635655	0.037015399
PTEN_Pathway_Apoptosis	-0.102386039	-0.185821968	0.073446266	0.00609218	0.027714773	0.003193333	0.060665508	0.023978761	-0.059440522	-0.319580151
PTEN_Pathway_Ca2+_Signaling	0.175597977	-0.082960355	-0.026580928	0.163111736	-0.033347749	-0.052251756	-0.162025848	-0.06592253	0.04635655	0.037015399
PTEN_Pathway_Cell_Cycle	0.102465966	0.08531593	-0.061975801	0.009796731	-0.011671469	-0.110871896	-0.078724833	-0.05646635	0.049906137	-0.130115559
PTEN_Pathway_Cell_Survival	0.055156516	-0.081903957	0.129780965	-0.119117322	-0.06679343	-0.188274298	-0.019412528	-0.220739375	-0.098731514	-0.162732745
PTEN_Pathway_Differentiation	0.101068567	0.081614903	-0.062718597	0.009403983	-0.011262373	-0.111839739	-0.077184153	-0.054554758	0.050096554	-0.130924668
PTEN_Pathway_DNA_Repair	0.093955671	0.129789006	-0.065415569	0.008349414	-0.019674525	-0.129315082	-0.059522503	-0.042733435	0.050656925	-0.140560295
PTEN_Pathway_Genomic_Stability	0.102589882	0.131807744	-0.063527523	0.012443878	-0.022369536	-0.128703062	-0.064641738	-0.042193099	0.047948912	-0.147336461
PTEN_Pathway_Growth	0.102465966	0.08531593	-0.061975801	0.009796731	-0.011671469	-0.110871896	-0.078724833	-0.05646635	0.049906137	-0.130115559
PTEN_Pathway_Migration	0.07491691	0.171204302	-0.074758223	0.019518188	-0.019606033	-0.099980536	-0.012462132	0.028380749	0.031909477	-0.108493692
PTEN_Pathway_Neuronal_Survival	0.125166731	-0.17129128	-0.047089458	-0.079973796	-0.012107943	-0.227266588	-0.077129286	-0.171169326	0.011159943	-0.293586669
PTEN_Pathway_Proliferation	0.070003034	0.150938013	-0.068989615	0.015173177	-0.01226936	-0.08030546	-0.031909747	0.017751395	0.043165692	-0.075093433
PTEN_Pathway_Proliferation_CyclinA2_Geminin_AuroraA_PLK1	0.023893318	-0.093988803	0.077477004	0.152777308	0.011173118	0.097055034	-0.142973964	-0.00369988	0.030430529	0.123329012
PTEN_Pathway_Protein_Synthesis	0.150856148	-0.219289668	0.115085554	-0.0998036	-0.014512802	-0.36347352	-0.219805926	-0.398584992	-0.031622997	-0.463425562
PTEN_Pathway_Senescence_Ets2_p16	0.023893318	-0.093988803	0.077477004	0.152777308	0.011173118	0.097055034	-0.142973964	-0.00369988	0.030430529	0.123329012
PTEN_Pathway_Synaptic_Transmission	0.104409218	0.155777524	-0.062031425	0.027706802	-0.033718515	-0.119808075	-0.059153614	-0.03026808	0.050494335	-0.119560131
RANK_Signaling_in_Osteoclasts_Pathway	0.119335652	0.049721834	0.011547215	0.070657028	-0.044033833	0.050981504	-0.075654964	0.118904742	-0.011194067	0.272501471
RANK_Signaling_in_Osteoclasts_Pathway_Expression_of_Osteoclastic_Genes_via	0.179820801	0.155933069	-0.007531309	0.123330187	-0.070208219	0.010925525	-0.097526758	0.118534026	-0.004554514	0.45407727
RANK_Signaling_in_Osteoclasts_Pathway_Inhibition_of_Death	0.019999487	-0.070181193	0.010790765	-0.075249038	-0.01992197	-0.021353263	0.024383854	0.035762753	-0.03525213	0.163421022
RANK_Signaling_in_Osteoclasts_Pathway_Resorption	0.042552356	-0.05328654	-0.029854825	0.043570405	-0.024799854	0.105833115	-0.017083915	0.130846824	0.029186241	0.159269684
Ras_Pathway	0.080438206	-0.103173293	0.017395353	0.07886821	0.001398224	0.037704745	-0.097223712	-0.05044172	-0.002008065	0.131880926
Ras_Pathway_Apoptosis	0.006922717	-0.011288737	-0.005631943	-0.002577274	-0.036888761	0.038548428	0.049383035	0.107221359	-0.013785054	0.173585071
Ras_Pathway_CDC42_Pathway	-0.194488073	-0.098035146	0.011339563	-0.23330744	0.090945611	0.204058521	0.087886762	0.042737843	0.004316174	0.143299072
Ras_Pathway_Cell-Cell_Junctions	-0.044924106	0.001382281	-0.072352592	-0.014472317	0.031804333	0.128677461	0.042957105	0.177187929	0.042515257	0.239936649
Ras_Pathway_Gene_Expression_Cell_Proliferation_Cell_Survival_Differentiation	0.027954385	0.075230876	0.017163866	-0.018818573	-0.045204814	0.066642568	0.007281113	0.047624319	-0.007194544	0.191967301
Ras_Pathway_Golgi_Trafficking_and_Vesicle_Formation	-0.093904864	0.12792458	-0.005309079	-0.101452984	0.058114012	0.126205169	-0.020306719	-0.025984139	0.061406667	0.22038467
Ras_Pathway_Increased_T-cell_Adhesion	0.071630879	-0.059372104	0.077727348	0.223942918	-0.023900182	0.044912422	-0.028182214	0.070446467	-0.097275839	0.116082329
Ras_Pathway_Receptor_Endocytosis	-0.109558264	0.095361379	0.070475908	-0.05160977	0.001795531	0.309922698	0.046697131	0.175655796	-0.009410333	0.40972275
Ras_Pathway_RhoA_Pathway	-0.053777038	-0.255186682	-0.025642061	-0.025319098	0.065674909	0.069473518	0.003679233	-0.110865014	0.010064962	0.123400686
SMAD_Signaling_Network_Pathway	0.192877893	0.161404256	-0.037313718	0.143624589	-0.035257073	0.085661408	-0.142803713	0.114873693	0.022496604	0.186334439
STAT3_Pathway	0.032666701	-0.053813046	-0.010313448	0.037535816	0.035926323	0.114369137	-0.077847698	-0.023190066	0.019568124	0.234785681
STAT3_Pathway_Anti-Apoptosis	-0.09792758	0.95074906	-0.182518329	0.720799059	-0.002945894	1.029735426	0.201206356	0.814550435	0.082185434	1.513342212
STAT3_Pathway_G1_to_S_Cell_Cycle_Progression	0.232187162	1.265535394	-0.311535031	0.996071515	-0.317423515	1.321247419	0.698455135	1.326425989	-0.301683693	1.690113574
STAT3_Pathway_Growth_Arrest_and_Differentiation	0.017874693	-0.090358375	-0.05673771	0.072957531	0.026927816	0.061881274	-0.038841378	-0.025120489	0.050776582	0.277620846
Target_drugs_pathway	0.024736361	-0.037146144	0.046436433	0.063494653	-0.007412967	0.07380108	-0.059069448	-0.00889415	-0.004690378	0.15924314
TGF-Beta_Pathway	0.048441226	-0.075753503	0.011600829	0.027166419	0.013365078	0.079682156	-0.077910179	-0.020012994	0.004503045	0.186805349
TGF-Beta_Pathway_Epithelial_Mesenchymal_Transdifferentiation	-0.028939297	-0.305022603	0.073638344	-0.110875792	-0.049015082	-0.143385265	0.023908512	-0.041051357	-0.019592487	-0.198437565
TGF-Beta_Pathway_Post_Transciptional_G1_Arrest	-0.053228744	0.05826637	0.038665494	-0.008188592	-0.014803127	0.088388087	0.073351381	0.035095301	-0.04398499	0.154390081
TGF-Beta_Pathway_Transciption_Arrested_Growth_Apoptosis	0.09304166	0.072609127	0.035892817	0.090615675	-0.04442592	0.182434199	-0.086919602	0.204276685	0.002411052	0.473339197
TGF-Beta_Pathway_Transciption_Cell_Growth_and_Mobility_and_Angiogenesis	0.09304166	0.072609127	0.035892817	0.090615675	-0.04442592	0.182434199	-0.086919602	0.204276685	0.002411052	0.473339197
TNF_Signaling_Pathway	0.039401981	0.240178643	-0.028710311	0.085581415	-0.030557947	0.238245415	0.037672389	0.240507672	-0.017806107	0.491879351
TNF_Signaling_Pathway_Apoptosis	-0.113051532	0.142907363	0.076612392	0.037818308	0.016543832	0.260372766	0.060926875	0.076682194	-0.041031565	0.222372251
TNF_Signaling_Pathway_Gene_Expression_and_Cell_Survival_via_FOS_NFKB2_JU	0.091995858	0.220867576	-0.055847292	0.086539643	-0.053017188	0.164458912	0.012844905	0.289582265	0.004023719	0.54421864
TRAF_Pathway	0.072851685	0.036519034	-0.007722159	0.049810059	-0.019658515	0.126523401	-0.032566014	0.098825146	-0.012904987	0.293497549
TRAF_Pathway_Apoptosis	-0.015372341	0.324951832	-0.036611696	0.05054947	0.072209531	0.532629787	0.080834799	0.257710815	-0.101060269	0.58942183

Supplementary Data S-IX

Complete list of all affected intracellular signaling pathways, including pathway activation strength (PAS) values after ALPPS in humans.

TRAF_Pathway_Cell_Survival	-0.005183325	-0.365147329	0.070005137	-0.122243903	-0.018114403	-0.157785577	-0.030210526	-0.072184294	-0.016496895	-0.180664509
TRAF_Pathway_Direct_Antimicrobial_Response_and_Cell-Mediated_Immunity_a	0.129465114	0.154343386	-0.082328374	-0.032619786	-0.003311648	-0.010366343	-0.066772732	0.071258711	0.022947659	0.316625856
TRAF_Pathway_Gene_Expression_via_FOS_JUN	0.373756326	0.333934218	-0.047092709	0.086384619	-0.09912455	0.145700169	-0.301787116	0.28251338	0.074248051	0.819128983
Ubiquitin-Proteasome_Dependent_Proteolysis_Pathway	-0.132700458	0.041587951	0.030861174	-0.020683786	0.010412049	0.122082847	0.117072943	0.090763534	-0.025645704	0.182079864
VEGF_Pathway	0.023866219	-0.069881838	-0.007431677	0.032141203	-0.013929429	0.084466295	-0.049797441	0.02428056	0.047292329	0.163239925
VEGF_Pathway_Actin_Reorganization	0.020594317	0.303547903	-0.038665518	0.287619918	-0.01090203	0.576048988	-0.049842164	0.352417889	0.078815393	0.709306225
VEGF_Pathway_Cell_Migration	0.020594317	0.303547903	-0.038665518	0.287619918	-0.01090203	0.576048988	-0.049842164	0.352417889	0.078815393	0.709306225
VEGF_Pathway_Cell_Survival	-0.031013927	-0.240576876	0.015328024	-0.005612968	-0.031061377	-0.032407375	0.050472816	0.040484892	-0.003725546	-0.062280879
VEGF_Pathway_Focal_Adhesion_Turnover	0.009720489	-0.504210846	0.015711665	-0.125211422	0.091112836	-0.251389373	-0.205961979	-0.250300472	0.089416989	-0.212796704
VEGF_Pathway_Gene_Expression_and_Cell_Proliferation_via_MAPK7	0.023112289	-0.022438087	0.002013824	-0.071337523	-0.046758279	-0.097179671	-0.022628039	-0.15614258	0.044260205	0.120388505
VEGF_Pathway_Nitric_Oxide_Production	-0.127763708	0.451682642	0.168880055	0.610438789	-0.206408327	0.475137842	0.150790592	0.389724642	0.014501403	1.051780979
VEGF_Pathway_Prostaglandin_Production	0.058511581	-0.113985962	-0.00170381	0.037182097	-0.032439038	-0.069218286	-0.075547961	-0.111088807	0.051179238	0.121259269
WNT_Pathway	0.101588858	-0.021535563	0.02640542	0.068683541	-0.021055249	-0.07089586	-0.117443891	-0.10804021	0.01050486	-0.027070436
WNT_Pathway_Cell_Fate_Proliferation_Differentiation_Adhesion_and_Survival	0.160203842	0.055876488	-0.008722461	0.080539997	-0.044043594	-0.159590703	-0.137014303	-0.108112744	0.029576516	-0.148394781
WNT_Pathway_Cell_Survival	0.023631904	0.169996283	0.028156953	0.092578052	-0.102783667	-0.02627131	0.014968705	0.094701632	0.03602609	0.161488275
WNT_Pathway_Cytoskeletal_Rearrangement	-0.07048134	-0.430580543	0.000984534	0.02496866	-0.044141774	-0.124416608	0.058308131	-0.092802955	0.055330399	0.124595715
WNT_Pathway_Gene_Expression_via_CREB3	0.045302751	-0.128551637	0.013582987	-0.028222908	0.000407449	-0.061224999	-0.058969941	-0.181915647	-0.000323246	-0.037114542
WNT_Pathway_NFAT_Pathway	0.139076045	-0.073043386	-0.006435422	-0.020667337	-0.004452592	-0.290127693	-0.114881028	-0.168651444	-0.013307	-0.047167131
WNT_Pathway_PKC_Pathway	0.078690455	-0.19457435	0.002293466	-0.03202734	0.009054975	-0.058516511	-0.102711548	-0.210172833	0.012672656	-0.049742705
